# Synthesis and Characterisation of Phosphino-Aryloxide Rare Earth Complexes

**DOI:** 10.3390/molecules29235757

**Published:** 2024-12-05

**Authors:** Elias Alexopoulos, Yu Liu, Alex W. J. Bowles, Benjamin L. L. Réant, Fabrizio Ortu

**Affiliations:** 1 School of Chemistry, University of Leicester, University Road, Leicester LE1 7RH, UK; ia248@leicester.ac.uk (E.A.); yl762@leicester.ac.uk (Y.L.); awjbowles@gmail.com (A.W.J.B.); 2 Department of Chemistry, The University of Manchester, Oxford Road, Manchester M13 9PL, UK

**Keywords:** lanthanide, rare earth, coordination chemistry, crystallography, NMR

## Abstract

A series of homoleptic rare earth (RE) complexes bearing phosphino-aryloxide ligands (**1-RE**, **2-La**) has been prepared. The complexes have been characterised using multinuclear NMR and IR spectroscopy, X-ray crystallography and elemental analysis. Structural characterisation highlighted the different RE–P interactions as a result of differing Lewis acidity and ionic size across the series, hinting at the possibility of FLP-type activity. The potential reactivity of these complexes has been tested by reacting them with small molecules (H_2_, CO, CO_2_). A series of side-products (**3-RE**) has also been observed, isolated and characterised, featuring the incorporation of a phosphonium-aryloxide ligand.

## 1. Introduction

Since the seminal report by Stephan and co-workers in 2006 [1], research on Frustrated Lewis Pairs (FLPs) has been extensive. The interest in FLP reactivity arises from the great appeal of utilising cheap, earth-abundant main group elements (e.g., B, N, Al, P) in reactions classically performed using expensive and scarce precious metals (e.g., Rh, Ir, Pd, Pt) [2,3,4,5,6]. Such remarkable reactivity derives from the simultaneous presence of a Lewis acid and a Lewis base, which are unable to form an adduct, thus leaving unquenched reactivity which can be exploited to activate small molecules such as H_2_ [7,8,9,10], CO [11], CO_2_ [12,13] and N_2_O [14,15], as well as larger organic molecules [16,17,18,19]. Investigations into this type of reactivity have led to developments within the field of homogeneous catalysis [4], polymer chemistry [20] and material science [21].

**Figure 1:**
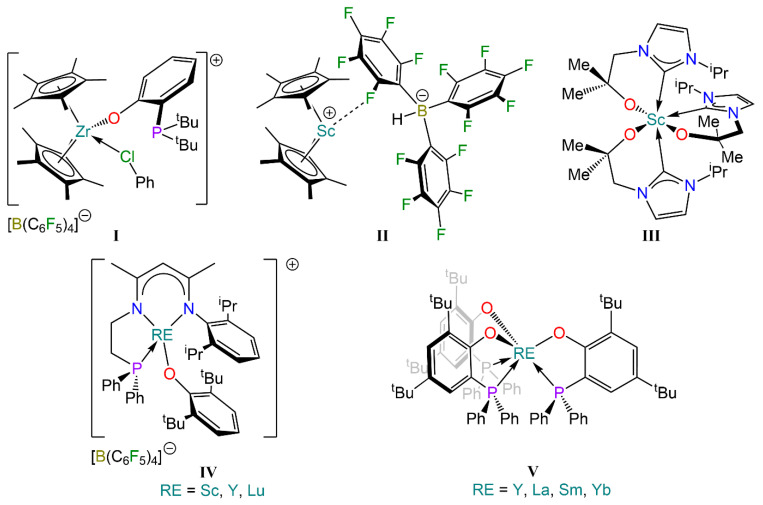
Examples of Zr (**I**) and RE complexes (**I**–**IV**) that display FLP-like reactivity, and selected phosphino-aryloxide complexes (**V**) [22,24,26,27,41].

Wass and co-workers extended this approach to closed shell metals La^3+^ and Zr^4+^, such as the cationic zirconocene complex incorporating a phosphino-aryloxide ligand, [Zr(Cp*)_2_{^t^Bu_2_P(C_6_H_4_)O}(C_6_H_5_Cl)][B(C_6_F_5_)_4_] (**I,** Figure 1), which displayed reactivity towards H_2_, CO, CO_2_, THF, acetone, alkenes, alkynes and alkyl halides [22,23]. Piers and co-workers further expanded this approach to lighter rare earth (RE) elements, reporting an FLP system consisting of a scandocenium cation and a hydroborate anion, [Sc(Cp*)_2_{HB(C_6_F_5_)_3_}] (**II**, Figure 1), which is capable of activating CO and CO_2_ [24,25]. Arnold and co-workers also developed an NHC-based scandium complex, [Sc{C[N(^i^Pr)CHCHN(CH_2_CMe_2_O)]}_3_] (**III**, Figure 1), that activates CO_2_ and CS_2_ [26].

Over the last decade, Xu and co-workers have further expanded the scope of RE FLPs to include lanthanide metals. Examples of such compounds include complex [RE{N(Dipp)C(Me)CHC(Me)N(CH_2_CH_2_PPh_2_)}][B(C_6_F_5_)_4_] (**IV**, Figure 1; RE = Sc, Y, Lu), which has been shown to act as an intramolecular FLP due to the combined properties of the Lewis acidic metal centre with a weakly-coordinating, Lewis basic phosphine ligand [27,28,29,30,31,32]. Similarly, the homoleptic tris-aryloxide complex [RE{O(2-6-^t^Bu_2_C_6_H_3_)}_3_] (RE = Sc, Y, La, Sm) can act as a Lewis acid in collaboration with a bulky Lewis base [33,34,35,36,37,38]. These RE FLP systems have been reported to activate small molecules but can also participate in organic transformations, and even act as catalysts for the synthesis of polymers.

Despite this remarkable progress in transition metal FLPs, research on RE analogues is still in its infancy [39,40]. Crucially, no studies have been reported on the differing reactivity which can be unlocked owing to the variations in Lewis acidity of the metal centres across the RE and lanthanide (Ln) family. Taking inspiration from Wass’ work on group 3 and group 4 phosphino-aryloxide complexes (**I**), we were intrigued by the possibility of incorporating multiple Lewis acid/base pairings within the same complex. Recently, Hlina and co-workers reported the synthesis of homoleptic RE complexes stabilised with the 2,4-di-*tert*-butyl-6-(diphenylphosphanyl)phenolate (OAr^P^) ligand, [RE(OAr^P^)_3_] (**V**, RE = Y, La, Sm, Yb, Figure 1), which were employed for the preparation of bimetallic complexes with late transition metals binding the phosphorus donors [41]. Herein we present our attempts to synthesise homoleptic phosphino-aryloxide complexes [RE{R**_2_**P(C**_6_**H**_4_**)O}_3_] (R = ^t^Bu, ^i^Pr) and our investigation of their reactivity with H_2_, CO and CO_2_.

## 2. Results and Discussion

### 2.1. Synthesis and Spectroscopic Characterisation

**Scheme 1:**
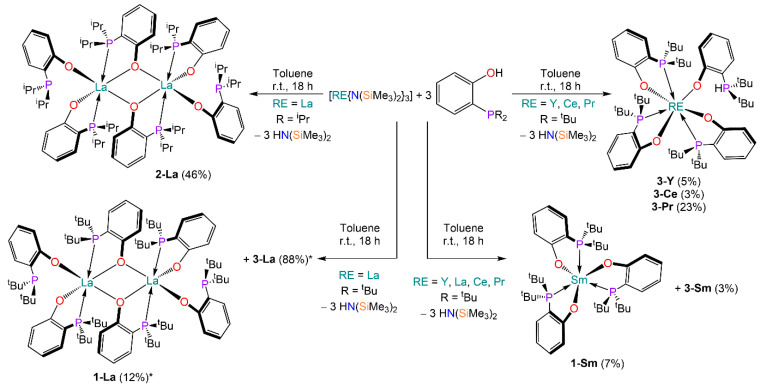
Synthesis of **1-RE**, **2-La** and **3-RE**. Crystalline yields are reported in brackets (* denotes spectroscopic yield).

The proligands 2-di-*tert*-butylphosphinophenol and 2-di-isopropyl-phosphinophenol were synthesised in good yields (62% and 64% respectively) by minor modification of previously reported methods [42]. In order to target [RE{R**_2_**P(C**_6_**H**_4_**)O}_3_] complexes, we reacted the proligands with selected RE *tris*-amides [RE{N(SiMe_3_)_2_}_3_] (RE = Y, La, Ce, Pr, Sm) in a 3:1 stoichiometric ratio. The outcome of the reactions varied significantly depending on the RE metal employed (Scheme 1). From the reaction between [La{N(SiMe_3_)_2_}_3_] and the proligands, i.e., 2-di-*tert*-butylphosphinophenol or 2-di-isopropyl-phosphinophenol, dimeric, homoleptic complexes [{La[R**_2_**P(C**_6_**H**_4_**)O]_2_[µ_2_-R**_2_**P(C**_6_**H**_4_**)O]}_2_] (**1-La**: R = ^t^Bu; **2-La**: R = ^i^Pr) were obtained. Conversely, the heaviest Ln metal studied, Sm, afforded the target homoleptic complex [Sm{^t^Bu**_2_**P(C**_6_**H**_4_**)O}_3_]. Interestingly, analogous species could not be obtained when performing the same reactivity with Y, Ce and Pr, as, in these cases, complexes of general formula [RE{^t^Bu**_2_**P(C**_6_**H**_4_**)O}_3_{^t^Bu**_2_**PH(C**_6_**H**_4_**)O}] (**3-RE**; RE = Y, Ce, Pr) were isolated, which incorporate the phosphonium-aryloxide ligand {^t^Bu**_2_**P^+^H(C**_6_**H**_4_**)O^−^}. Complex **3-Ce** could not be isolated in pure form, as it could not be separated from co-crystallised free ligand and [Ce{N(SiMe_3_)_2_}_3_]. Additionally, when reacting [La{N(SiMe_3_)_2_}_3_] with 2-di-*tert*-butylphosphinophenol in an attempt to synthesise **1-La**, a ^1^H and ^31^P{^1^H} NMR analysis of the crude reaction mixture showed that more than one species was present (Figure S46). Therefore, from the crude reaction mixture, compound [La{^t^Bu**_2_**P(C**_6_**H**_4_**)O}_3_{^t^Bu**_2_**PH(C**_6_**H**_4_**)O}] (**3-La**) was recrystallised from toluene, as confirmed by X-ray crystallography. Similarly, [Sm{^t^Bu**_2_**P(C**_6_**H**_4_**)O}_3_{^t^Bu**_2_**PH(C**_6_**H**_4_**)O}] (**3-Sm**) was obtained from fractional crystallisation during the synthesis of **1-Sm**. All complexes were authenticated using single crystal X-ray studies (*vide infra*) and further analysed via multinuclear NMR spectroscopy, IR spectroscopy and elemental analysis.

The ^1^H NMR spectra of compounds **1-La** and **2-La** each contained four characteristic peaks of equal integration in the aromatic region (**1-La**: δ_H_ = 6.65, 6.91, 7.28 and 7.39 ppm; **2-La**: δ_H_ = 6.68, 6.80, 7.12 and 7.25 ppm), each corresponding to an individual proton in the aromatic ring. The ortho-substitution pattern of the aromatic ring made the multiplicity of each peak difficult to analyse due to the presence of multiple coupling patterns and second order effects. Besides the aromatic peaks, the aliphatic region of **1-La** contained a doublet corresponding to the CH_3_ protons coupling to the phosphorus atom (δ_H_ = 1.27, ^3^*J*_HP_ = 12 Hz). The aliphatic region of the ^1^H NMR spectrum of **2-La** showed a complex multiplet corresponding to the tertiary proton (δ_H_ = 2.08), as well as two doublets of doublets (δ_H_ = 1.04, 1.18, ^3^*J*_HP_ = 12.5, 15.5 Hz, ^3^*J*_HH_ = 7 Hz), corresponding to two pairs of CH_3_ protons coupling to the tertiary proton (^3^*J*_HH_) and the phosphorus atom (^3^*J*_HP_). Interestingly, in the aryl region of the ^1^H spectrum of **1-La**, some additional smaller signals were observed (Figure S1A) which we attributed to different states of aggregation of the compound in solution, and possibly an interconversion between terminal and bridging ligands of the dimeric structure. The ^1^H spectrum recorded for a sample of **1-La** in THF-d_8_ showed a more simplified aryl region with no additional signals (Figure S1B); it is therefore reasonable to assume that the dimeric structure of **1-La** was broken in coordinating solvents, likely leading in this case to a THF-adduct of monomeric ‘La{^t^Bu**_2_**P(C**_6_**H**_4_**)O}_3_’. It is also very likely that **2-La** could display similar behaviour in the presence of coordinating solvents, and it is worth pointing out that both **1-La** and **2-La** are only sparingly soluble in benzene-d_6_, requiring use of a few drops of THF-d_8_ to prepare concentrated samples for ^13^C NMR analysis.

Notably, ^31^P NMR (*I* = ½, 100% abundant) spectroscopy is of particular diagnostic value for these systems; it can be used to offer a qualitative estimate of the interaction between the phosphorus atom and the metal centre. In principle, the more downfield the chemical shift of the ^31^P peak for a metal complex relative to that for a proligand (assuming that the metal centre is the only entity that interacts with the phosphorus atom upon complexation), the stronger the interaction. This is particularly notable, as the extent of interaction between a Lewis acid and a Lewis base tends to have an impact on FLP-type reactivity [5]. The ^31^P{^1^H} NMR spectra of the proligands 2-di-*tert*-butylphosphinophenol and 2-di-isopropyl-phosphinophenol showed a single peak at −6.62 and −24.42 ppm respectively, whereas the ^31^P{^1^H} NMR spectra of **1-La** and **2-La** in C_6_D_6_ showed one peak at 30.11 ppm and 1.21 ppm respectively; these resonances were significantly shifted compared to corresponding signals of the proligands (Δδ_P_ = 36.81 ppm and 25.63 ppm respectively). Analogous differences in the ^31^P NMR chemical shift between free proligand and metal-bound phosphino-aryloxide were reported by Wass and co-workers; notably the largest difference (Δδ_P_ = 71.06 ppm) was measured for complex [Zr(Cp)_2_{^t^Bu_2_P(C_6_H_4_)O}(C**_6_**H**_5_**Cl)][B(C**_6_**F**_5_**)**_4_**] and the corresponding proligand [22], and that complex was shown to activate CO_2_ via FLP-type reactivity [23]. Both **1-La** and **2-La** exhibited a higher chemical shift compared to compound **V**, as reported by Hlina and co-workers (δ (C_6_D_6_) = −0.3 ppm) [41]. The ^31^P{^1^H} NMR spectrum of **1-Sm** showed a peak at −56.71 ppm (Figure S7), likely broadened due to paramagnetic effects.

The ^1^H NMR spectra of **3-La** showed some notable changes compared to **1-La** (Figure S25). The four peaks within the aromatic region corresponding to the four hydrogen atoms of the aryl moiety were still present, but three of them were broadened. A similar broadening was also observed for the signals of the methyl groups of the *tert*-butyl substituents. Furthermore, an additional broad signal could be observed at 8.40 ppm, which corresponded to the phosphonium hydrogen. This signal was likely to be a doublet; however, the signal partially overlapped with other peaks in the aromatic region. This line-broadening was also observed with all signals in the ^13^C{^1^H} NMR spectrum of **3-La** to varying extent (Figure S15). The ^1^H NMR spectrum of **3-Y** was very similar to that of **3-La** (Figure S10), with two key differences. Firstly, two broad signals could be observed for the methyl groups of the *tert*-butyl substituents with a 3:1 integration ratio. Secondly, the phosphonium signal resonated with a broad signal at 7.58 ppm (^1^*J*_PH_ = 276 Hz). The ^31^P NMR spectra of **3-Y** displayed two signals resonating at 10.55 ppm and 18.25 ppm (Figure S13), which we ascribed to the phosphine and phosphonium groups, respectively.

Due to the paramagnetic nature of **3-Ce** and **3-Pr**, it was difficult to extract any information from the ^1^H NMR spectra of these complexes. Nonetheless, in the case of **3-Pr**, a broad doublet was observed at 96.78 ppm (Figure S19B), which could be tentatively assigned to the phosphonium proton (^1^*J*_PH_ = 474 Hz). Similar to what was observed with **1-Sm**, the ^1^H NMR spectrum of **3-Sm** was decipherable (Figure S20B), displaying four broad peaks in the aromatic region and, like in the case of **3-Pr**, a broad doublet (^1^*J*_PH_ = 488 Hz) at 15.32 ppm corresponding to the phosphonium proton. There were also two broad CH_3_ alkyl signals with a relative integration of 1:3. In this case, the CH_3_ alkyl peak of the *tert*-butyl group bound to the phosphonium P atom had been shifted downfield relative to the other *tert*-butyl group. For **3-Ce** and **3-Sm**, we were also able to acquire ^31^P NMR data. The spectrum of **3-Ce** showed a single weak resonance at 40.54 ppm (Figure S18), whilst in the case of **3-Sm**, two broad signals were detected at −44.34 and −21.95 ppm (Figure S21).

**Scheme 2:**
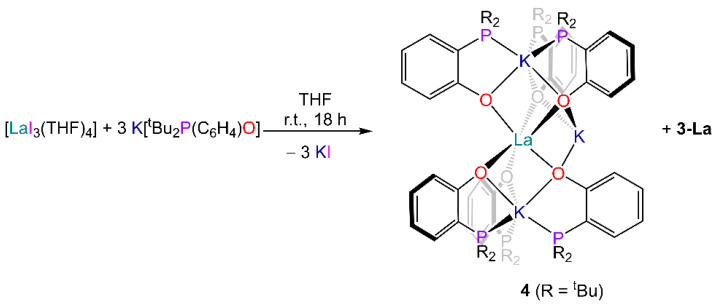
Synthesis of **4**.

The problems encountered when targeting **1-RE** complexes related to the formation of unexpected side-products **3-RE** may have arisen from the choice of starting materials; it is likely that the OH proton of the phosphinophenol provided a pathway for the formation of the phosphonium-phenolate ligand via intra- or intermolecular protonation of the phosphine functionality. To prove this, we attempted an NMR-scale reaction between [La{N(SiMe_3_)_2_}] and four equivalents of di-*tert*-butylphosphinophenol, resulting in the formation of **3-La** as the major product and **1-La** as the minor product (8:1 ratio, Figure S29). Therefore, an alternative route was explored involving a salt metathesis reaction between the potassium salt K[^t^Bu**_2_**P(C**_6_**H**_4_**)O] and [LaI_3_(THF)_4_] (Scheme 2). However, a ^1^H and ^31^P NMR analysis of the crude mixture obtained from the reaction revealed the presence of traces of **3-La** (which was also confirmed via X-ray crystallography studies on recrystallised material), together with another species which was identified as the ‘ate’ complex [La{^t^Bu**_2_**P(C**_6_**H**_4_**)O}_6_K_3_] (**4**) via X-ray crystallography (see SI).

### 2.2. Structural Characterisation

**Figure 2:**
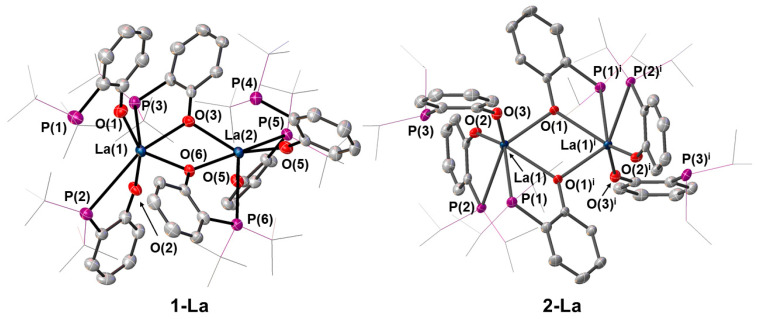
Crystal structures of **1-La** and **2-La**. Ellipsoids are shown at 30% probability level, hydrogen atoms are omitted, *tert*-butyl and *iso*-propyl groups are shown as wireframe for clarity. Symmetry operation used to generate equivalent atoms: i = 1 − *x*, 1 − *y*, 1 − *z*.

Compounds **1-La** and **2-La** both crystallised in the *P*-1 space group and displayed an analogous dimeric arrangement in the solid state, with two phosphinophenolate ligands bridging between the metal centres via the oxygen donor atoms (Figure 2). As a result, the La–O distances of bridging ligands [**1-La**: La–O 2.418(6)–2.526(5) Å; **2-La**: La–O 2.458(2)-2.477(2) Å] were elongated slightly with respect to those of terminal donors [**1-La**: La–O 2.235(6)–2.250(6) Å; **2-La**: La–O 2.255(2)–2.297(2) Å]. The distances between the metal centres and the coordinated phosphorus atoms for **1-La** were larger than those for **2-La** (Table 1), ranging from 3.223(2) Å to 3.459(3) Å for **1-La**, and 3.1653(10) Å to 3.3466(7) Å for **2-La**. Both metal centres in **1-La** exhibited a six-coordinate distorted trigonal prismatic geometry; in each half of the dimer, one of the ligands acts as a monodentate *O*-donor, with the phosphorus atom positioned away from the metal centre [La···P 3.662(2) Å and 4.701(2) Å]. A similar arrangement was also observed for **2-La**, but in this case, the geometry of each metal centre was trigonal prismatic, with an inversion centre position between the two La atoms. The distance between the non-coordinating P atom and the respective La centre for **2-La** was found to be 3.4732(7) Å.

**Figure 3:**
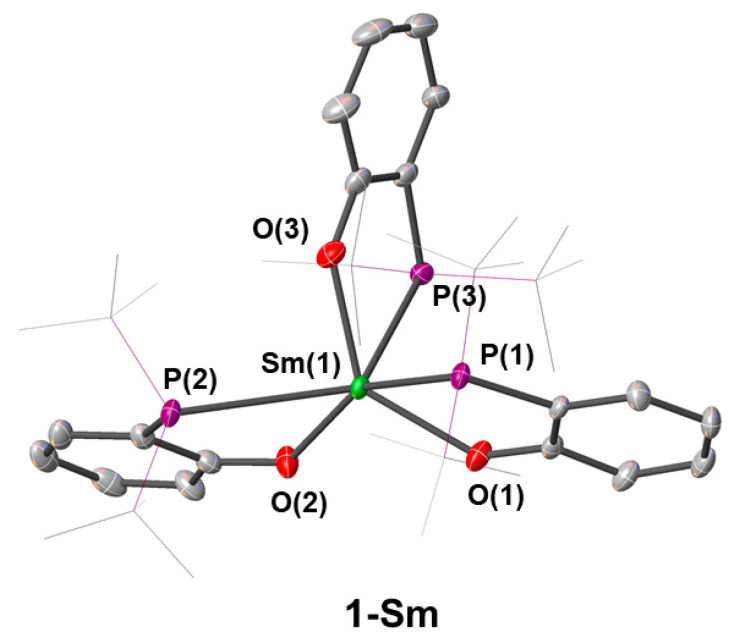
Crystal structure of **1-Sm**. Ellipsoids are shown at 30% probability level, hydrogen atoms are omitted and *tert*-butyl groups are shown as wireframe for clarity.

Complex **1-Sm** crystallised in the *P*2_1_/*n* space group and exhibited a six-coordinate distorted trigonal prismatic geometry (Figure 3). As expected, Sm was coordinated by three oxygen donors [Sm–O 2.184(2)–2.205(3) Å] and all phosphorus donors were also interacting with the metal centre [Sm–P 3.0552(8)–3.1503(8) Å]. This was reminiscent of the structure of [Sm(OAr^P^)_3_], as reported by Hlina and co-workers, where the Sm–P bond distance [3.1132(8) Å] was within the range of those measured for **1-Sm**. [Sm(OAr^P^)_3_] also features a distorted trigonal prismatic geometry, where the vertexes of each trigonal face are occupied by either three oxygen atoms or three phosphorus atoms [41]. The conformation of **1-Sm** is significantly different, where one of the ligands was flipped and the two vertexes of the trigonal prism were either *PPO* or *OOP*, likely to minimise the steric clash between the bulky *tert*-butyl substituents on the phosphine groups. This coordination motif was also reminiscent of the homoleptic phosphino-alkoxide complexes [RE{OC(^t^Bu)_2_CH_2_PMe_2_}_3_] (RE = Y, Nd), as reported by Lappert and co-workers [43].

**Figure 4:**
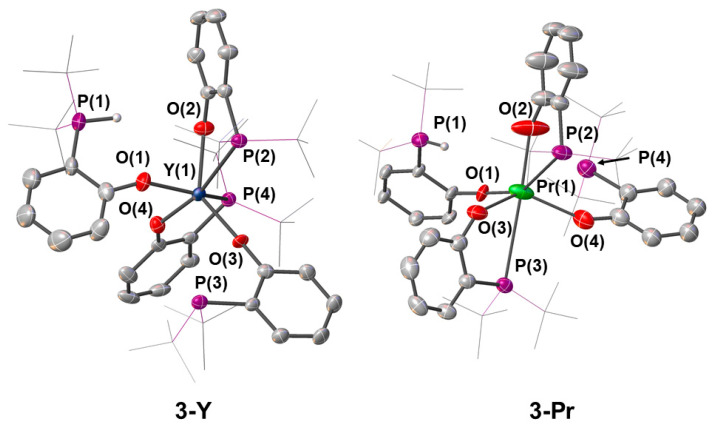
Crystal structures of **3-Y** and **3-Pr**. Ellipsoids are shown at 30% probability level, hydrogen atoms are omitted and *tert*-butyl groups are shown as wireframe for clarity, with the exception of phosphonium proton.

Complex **3-Y** crystallised in the *P*-1 space group, with two of the four phosphorus donors not coordinating to the metal centre, one of which was the protonated phosphorus atom (Figure 4). The Y–P distances were in the range between 3.0963(11) Å and 3.1926(10) Å, a phosphine group unbound [Y···P 3.627(3)–4.5242(9) Å] and the phosphonium centres were also positioned away from the metal centre [Y···PH 4.093(2)–4.2902(14)]. As a result, the overall coordination geometry of this compound was a distorted trigonal prismatic. This solid-state arrangement was similar to that exhibited by **3-Pr** (Figure 4), which features a phosphonium P–H bond far from the metal centre [Pr···P(1) 4.040(6)Å—Table 2] and a free phosphine group not-coordinating [Pr···P 3.451(5)]; this gave overall a coordination number of six for the complex and a very distorted octahedral geometry. The only other crystallographically authenticated Pr-phosphine complex was the phosphine-supported anilide [Pr{N(Mes)[C_6_H_3_(Me)(P^i^Pr_2_)]}_2_(OCP)] reported by Yu and co-workers [44], in which both Pr–P interactions were shorter [Pr–P 3.0647(7) Å and 3.1093(7) Å] than those measured in **3-Pr**.

**Figure 5:**
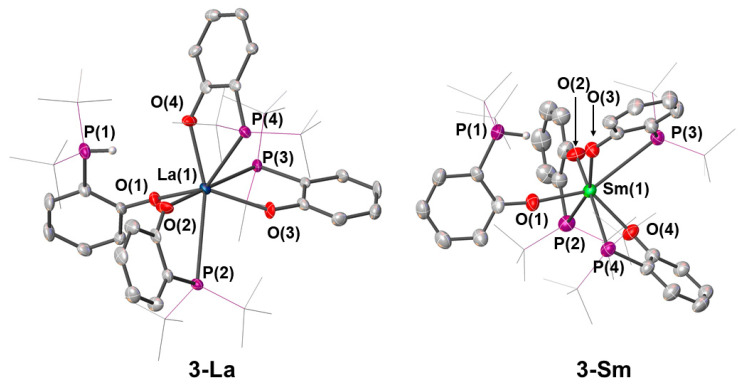
Crystal structures of **3-La** and **3-Sm**. Ellipsoids are shown at 30% probability level, hydrogen atoms are omitted and *tert*-butyl groups are shown as wireframe for clarity, with the exception of phosphonium proton.

Unlike **3-Y** and **3-Pr**, in **3-Sm**, the metal centre was coordinated by three phosphorus donors (Figure 5), with the phosphonium group exhibiting no interaction with the metal centre [Sm···P(3) 4.2693(13) Å]. This afforded a seven-coordinate distorted pentagonal bipyramidal geometry, with the Sm–P bond distances for the coordinating phosphorus atoms ranging from 3.2328(13) Å to 3.483(12) Å. Like in **3-Sm**, the phosphonium group in **3-La** was not interacting with the metal centre [La···P(4) 4.3034(8) Å]. As a result, the compound adopted a seven-coordinate distorted pentagonal bipyramidal geometry, with the La–P distances for the coordinating phosphorus atoms ranging from 3.2937(6) Å to 3.3646(8) Å. Unlike other **3-RE** analogues, **3-Ce** contains three non-coordinating phosphine groups. However, due the poor quality of the dataset, it was not possible to make meaningful comparisons with other complexes reported in this work.

## 3. Reactivity Studies

The original aim of our work was to test the reactivity of target compounds [RE{R_2_P(C_6_H_4_)O}_3_] with small molecules. However, only three of these compounds could be obtained, i.e., **1-La**, **2-La** and **1-Sm**, and amongst these, only **2-La** was isolated in reasonable quantities and purity for further reactivity studies. The issues with complex **1-La** became more evident when we tested its reactivity with CO and CO_2_, affording in all cases **1-La** and phosphonium complex **3-La** (one of the trace impurities present in the samples). Complex **2-La** was then chosen as a better candidate for more in-depth small molecule activation studies, and its reactivity was tested with H_2_, CO or CO_2_ gas. In all cases, ^1^H and ^31^P NMR analysis confirmed that no reaction had taken place, as no changes were observed in the chemical shifts of complex **2-La**. These observations were further evidenced by obtaining a crystalline solid from the reaction mixture with the same unit cell as the RE starting material, as confirmed by X-ray crystallography. We also decided to test the reactivity of one of the phosphonium derivatives and chose **3-Y** because of: (1) the diamagnetic nature of Y and possibility of performing reaction monitoring via NMR spectroscopy; (2) the highest Lewis acidity of the metal centre across the **3-RE** family; and (3) the potential for different reactivity and cooperativity due to the presence of phosphonium group. Complex **3-Y** was tested for the activation of H_2_, CO and CO_2_, but no reaction was observed in all cases, as evidenced by ^1^H and ^31^P NMR, as well as X-ray crystallography.

Providing an accurate explanation for the lack of reactivity in these species is not straightforward. Typically, FLP-type reactivity is more often observed when interactions between the Lewis acid and the Lewis base are minimised to allow the Lewis acid and Lewis base to react with a different molecule [5]. The complexes investigated herein displayed a variety of metal-phosphorus interactions, some of which were particularly elongated, which could be a desirable feature for FLP-type reactivity. Nonetheless, **1-La**, **2-La** and all **3-RE** compounds had very high coordination numbers and displayed a high degree of electronic and steric saturation of the metal centres, which likely posed a significant thermodynamic and kinetic barrier towards the association with small molecules and their activation. Should this be the reason for the lack of FLP-type reactivity, future RE complexes based on phosphino-aryloxides should either have different steric properties or be employed in conjunction with ligands that could reduce the electronic saturation of the RE metal, in a similar vein to what was reported by Wass and co-workers [23].

**Scheme 3:**
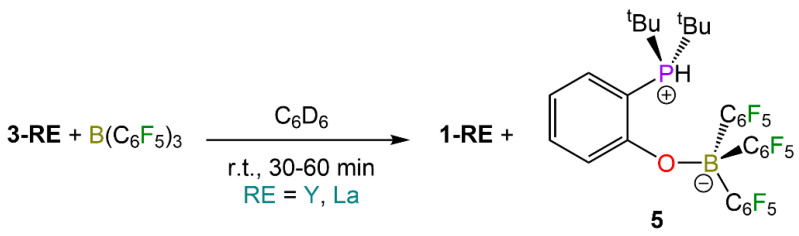
Reactivity of **3-RE** (RE = Y, La) with B(C_6_F_5_)_3_ and formation of **1-RE** and **5**.

We also investigated the possibility of abstracting the zwitterionic phosphonium ligands from **3-RE** with a secondary Lewis acid. The reactivity of **3-RE** (Ln = Y, La) with one equivalent of B(C_6_F_5_)_3_ was investigated on an NMR-scale (Scheme 3). In the case of the reaction between **3-La** and B(C_6_F_5_)_3_, ^1^H and ^31^P NMR analysis revealed the presence of **1-La** (Figures S31–S35). Similarly, **3-Y** also reacted with B(C_6_F_5_)_3_, forming what is likely to be **1-Y** (Figures S36–S40). In both reactions, the same side-product was also formed, as proven by multinuclear NMR spectroscopy. This side-product was found to be the zwitterionic adduct {^t^Bu**_2_**PH(C**_6_**H**_4_**)O}B(C_6_F_5_)_3_ (**5**) formed between B(C_6_F_5_)_3_ and ^t^Bu**_2_**P^+^H(C**_6_**H**_4_**)O^−^. This was further confirmed by reacting 2-*tert*-butylphosphinophenol and B(C_6_F_5_)_3_, which led to the quantitative formation of **5**. The presence of a phosphonium group was confirmed via ^1^H, ^31^P and ^31^P{^1^H} NMR spectroscopy (^1^*J*_PH_ = 480 Hz) (Figures S41, S44 and S45). These results further support the occurrence of proton transfer between the hydroxyl and phosphine group, likely facilitated by the presence of a Lewis acid. Therefore, the reactivity of **3-RE** with B(C_6_F_5_)_3_ could potentially offer an alternative approach to obtain the target homoleptic complexes **1-RE**.

## 4. Conclusions and Future Work

In summary, a series of RE complexes containing phosphino-aryloxide ligands has been synthesised and characterised by NMR spectroscopy, IR spectroscopy, CHN elemental analysis and X-ray crystallography. In some cases, the ligands acted as monodentate *O*-donors and the phosphine groups did not coordinate to the metal centre, likely as a result of steric congestion of the metal coordination sphere or electronic saturation. The synthesis of homoleptic phosphino-aryloxide RE complexes (**1-La**, **2-La** and **1-Sm**) tended to be low-yielding, which can be attributed to the additional formation of the heteroleptic side-products **3-RE**. Additionally, **1-La** and **1-Sm** exhibited similar solubility to their **3-RE** counterparts, making the isolation of either product through recrystallisation challenging. Although it could be predicted that weak RE–P interactions could promote FLP-type activity, experimental evidence suggests that the complexes reported herein do not show any reactivity with H_2_, CO or CO_2_, likely due to the high degree of saturation of the metal coordination sphere. Interestingly, reactivity of **3-Y** and **3-La** with B(C_6_F_5_)_3_ led to the formation of **1-Y** and **1-La,** respectively, together with the borate-phosphonium adduct **5**. Future work will aim to synthesise novel RE complexes with differing steric features and the incorporation of ancillary ligands, aimed at reducing the electronic saturation of the rare earth metal centres.

## 5. Experimental

### 5.1. General Methods

THF and toluene were passed through columns containing molecular sieves, then stored either over a potassium mirror (toluene) or over 4 Å molecular sieves (THF) and thoroughly degassed prior to use. Hexane and diethyl ether were purchased anhydrous from Tokyo Chemical Industry (TCI-UK, Oxford, UK), dried over activated molecular sieves for 7 days, then stored over a potassium mirror. For NMR spectroscopy, C_6_D_6_ and C_4_D_8_O were dried by refluxing over potassium and then vacuum transferred and degassed by three freeze-pump-thaw cycles before use. ^n^BuLi was purchased from Merck (Rahway, NJ, USA) and used as received. [RE{N(SiMe_3_)_2_}_3_] complexes (RE = Y, La, Ce, Pr, Sm) were prepared according to literature procedures [45]. LaI_3_(THF)_4_ was synthesised according to a procedure described in the literature [46]. NMR spectra were recorded on either a Bruker Avance III HD 400 or Bruker Avance III 500 spectrometer (Bruker, Karlsruhe, Germany) operating at 400.07/500.13/800.19 (^1^H), 100.60/125.78/201.21 (^13^C{^1^H}), 79.48 (^29^Si{^1^H}) and 161.98/202.45 (^31^P{^1^H}) MHz. NMR spectra were recorded at 298 K unless otherwise stated and were referenced to residual solvent signals. FTIR spectra were recorded on a Bruker Alpha II spectrometer (Bruker, Karlsruhe, Germany) with Platinum-ATR module. Elemental microanalyses were carried out by the Elemental Analysis Service at London Metropolitan University.

### 5.2. Synthesis of 2-Di-tert-butylphosphinophenol

First, 2-Bromoanisole (2.2 mL, 17.5 mmol) was added to a flame-dried Schlenk flask and dissolved in diethyl ether (50 mL). The flask was cooled to 0 °C and, while stirring, ^n^BuLi (8 mL, 2.5 M in hexanes, 20 mmol) was added dropwise. The reaction mixture was stirred for a further 2 h at room temperature. The flask was cooled again to 0 °C and di-*tert*-butylchlorophosphine (3.4 mL, 17.9 mmol) was added. The resulting mixture was allowed to warm to room temperature and stirred for a further 2 h. The resulting suspension was filtered through celite and the volatiles were removed *in vacuo*, affording an orange oily residue which was identified by ^1^H and ^31^P{^1^H} NMR spectroscopy as di-*tert*-butyl(2-methoxyphenyl)phosphane. The oil was dissolved in dichloromethane (50 mL) and the solution was cooled to 0 °C. BBr_3_ (3.6 mL, 37.9 mmol) was added dropwise, and the resulting mixture was warmed to room temperature and stirred for 18 h. The volatiles were removed *in vacuo* and methanol (20 mL) was added. The resulting solution was transferred to a flame-dried ampoule fitted with a Rotaflo valve and stirred under reflux for 5 h. The solution was transferred to a flame-dried Schlenk flask and dried *in vacuo*. The residue was dissolved with diethyl ether (70 mL), and triethylamine (5 mL) was added. The resulting suspension was stirred at room temperature for 1 h, and the volatiles were removed *in vacuo* to afford a colourless solid and an oil. The oil was extracted with hexane (3 × 10 mL), and the filtrate was dried *in vacuo* to afford a yellow oil, which crystallised at room temperature upon standing to yield the product 2-di-*tert*-butylphosphinophenol as an off-white solid (2.522 g, 11.0 mmol, 62%). NMR spectroscopic data were previously recorded in the literature from samples in CD_2_Cl_2_ [42]. ^1^H NMR (C_6_D_6_, 298 K, 400 MHz): δ/ppm = 1.07 (18 H, d, ^3^*J*_PH_ = 13 Hz, C(C*H*_3_)_3_), 6.74 (1 H, m, Ar-C*H*), 7.11 (2 H, m, Ar-C*H*), 7.47 (1 H, m, Ar-C*H*), 8.10 (1 H, broad, O*H*). ^13^C{^1^H} NMR (C_6_D_6_, 298 K, 100 MHz): δ/ppm = 30.57 (d, ^2^*J*_PC_ = 13 Hz, C(*C*H_3_)_3_), 32.6 (d, ^1^*J*_PC_ = 14 Hz, *C*(CH_3_)_3_), 115.6 (d, ^3^*J*_PC_ = 1 Hz, Ar-*C*H), 119.4 (Ar-*C*H), 131.9 (Ar-*C*H), 134.6 (d, ^3^*J*_PC_ = 2 Hz, Ar-*C*H). ^31^P{^1^H} NMR (C_6_D_6_, 298 K, 162 MHz) δ/ppm = −6.62. FT-IR: $\tilde{v}$/cm^−1^ = 3261, 3075, 2958, 2894, 2960, 1601, 1571, 1469, 1446, 1386, 1359, 1280, 1230, 1208, 1170, 1155, 1125.

### 5.3. Synthesis of 2-Di-isopropylphosphinophenol

First, 2-Bromoanisole (2.2 mL, 17.5 mmol) was added to a flame-dried Schlenk flask and dissolved in diethyl ether (50 mL). The flask was cooled to 0 °C and, while stirring, ^n^BuLi in hexanes (7 mL, 2.5 M, 17.5 mmol) was added dropwise. The reaction mixture was stirred for a further 2 h at room temperature. The flask was cooled again to 0 °C and di-iso-propylchlorophosphine (2.8 mL, 17.6 mmol) was added. The resulting mixture was allowed to warm to room temperature and stirred for a further 2 h. The resulting suspension was filtered through celite and the volatiles were removed *in vacuo*, affording an orange oily residue which was identified by ^1^H and ^31^P{^1^H} NMR spectroscopy as di-iso-propyl(2-methoxyphenyl)phosphane. The oil was dissolved in dichloromethane (30 mL) and the resulting solution was cooled to −78 °C. Next, BBr_3_ (4 mL, 41.5 mmol) was added dropwise, and the resulting mixture was warmed to room temperature and stirred for 18 h. The volatiles were removed *in vacuo* and methanol (18 mL) was added. The resulting solution was transferred to a flame-dried ampoule fitted with a Rotaflo valve and stirred under reflux for 5 h. The solution was transferred to a flame-dried Schlenk flask and dried *in vacuo*. The residue was dissolved in diethyl ether (40 mL) and triethylamine (3.6 mL). The resulting suspension was stirred at room temperature for 1 h, and the volatiles were removed *in vacuo* to afford a colourless solid and an oil. The oil was extracted with hexane (3 × 10 mL), and the filtrate was dried *in vacuo* to afford 2-di-isopropyl-phosphinophenol as a dark orange oil (2.302 g, 11.0 mmol, 64%). NMR spectroscopic data were previously recorded in the literature from samples in CD_2_Cl_2_ [42]. ^1^H NMR (C_6_D_6_, 298 K, 400 MHz): δ/ppm = 0.80 (6 H, dd, ^3^*J*_PH_ = 12 Hz, ^3^*J*_CH_ = 7 Hz, CH(C*H*_3_)_2_), 0.96 (6 H, dd, ^3^*J*_PH_ = 16 Hz, ^3^*J*_CH_ = 7 Hz, CH(C*H*_3_)_2_), 1.86 (2 H, m, C*H*(CH_3_)_2_), 6.79–6.75 (1 H, m, Ar-C*H*), 7.10–7.07 (3 H, m, Ar-C*H*), 8.34 (1 H, broad, OH). ^13^C{^1^H} NMR (C_6_D_6_, 298 K, 100 MHz): δ/ppm = 19.1 (d, ^1^*J*_PC_ = 7 Hz, *C*H(CH_3_)_2_) 20.4 (d, ^2^*J*_PC_ = 18 Hz, CH(*C*H_3_)_2_), 23.4 (d, ^1^*J*_PC_ = 7 Hz, CH(*C*H_3_)_2_), 116.0 (d, ^2^*J*_PC_ = 1 Hz, Ar-*C*H), 120.4 (Ar-*C*H), 131.8 (Ar-*C*H), 133.2 (d, ^2^*J*_PC_ = 2 Hz, Ar-*C*H). ^31^P{^1^H} (C_6_D_6_, 298 K, 162 MHz): δ/ppm = −24.48; FT-IR: $\tilde{v}$/cm^−1^ = 3371, 3069, 3025, 2952, 2926, 2867, 1598, 1573, 1470, 1448, 1381, 1363, 1348, 1282, 1197, 1153.

### 5.4. Synthesis of [{La[^t^Bu_2_P(C_6_H_4_)O]_2_[µ-^t^Bu_2_P(C_6_H_4_)O]}_2_] (***1-La***)

A flame-dried Schlenk flask was charged with [La{N(SiMe_3_)_2_}_3_] (0.745 g, 1.2 mmol) and 2-(di-*tert*-butylphosphino)phenol (0.858 g, 3.6 mmol). Toluene (60 mL) was added, and the reaction mixture was stirred at room temperature for 18 h. The volatiles were removed *in vacuo* and the solid residue was washed with hexane (2 × 10 mL). The solid was recrystallised from toluene (10 mL, room temperature), affording **1-La** as a white crystalline solid, with an additional crystalline crop obtained from the hexane washings (0.096 g, 0.11 mmol, 9%). ^1^H NMR (C_6_D_6_, 298 K, 400 MHz): δ/ppm = 1.27 (54 H, d, ^3^*J*_PH_ = 12 Hz, C(C*H*_3_)_3_), 6.65 (1 H, m, Ar-C*H*), 6.91 (3 H, m, Ar-C*H*), 7.28 (3 H, t, ^3^*J*_HH_ = 8 Hz, Ar-C*H*), 7.39 (3H, m, Ar-C*H*). ^13^C{^1^H} NMR (C_6_D_6_/C_4_D_8_O, 298 K, 100 MHz): δ/ppm = 30.7 (broad, C(*C*H_3_)_3_), 33.7 (broad, *C*(CH_3_)_3_), 115.2 (Ar-*C*H), 119.0 (Ar-*C*H), 123.4 (Ar-*C*O), 131.5 (Ar-*C*H), 134.8 (Ar-*C*H), 173.6 (d, ^1^*J*_PC_ = 26 Hz, Ar-*C*P); ^31^P{^1^H} (C_6_D_6_, 298 K, 162 MHz): δ/ppm = 30.19. A satisfactory elemental analysis could not be achieved due the presence of by-products (**3-La**, *vide infra*), which could not be separated from the starting material. FT-IR: $\tilde{v}$/cm^−1^ = 2985, 2955, 2936, 2921, 2892, 2858, 1579, 1454, 1427, 1363, 1287, 1257, 1243, 1230, 1164, 1119, 1033, 853.

### 5.5. Synthesis of [{La[^i^Pr_2_P(C_6_H_4_)O]_2_[µ-^i^Pr_2_P(C_6_H_4_)O]}_2_] (***2-La***)

A flame-dried Schlenk flask was charged with [La{N(SiMe_3_)_2_}_3_] (1.797 g, 2.9 mmol) and dissolved in toluene (50 mL). In a separate Schlenk flask, 2-(di-isopropylphosphino)phenol (1.829 g, 8.7 mmol) was dissolved in toluene (15 mL), and the resulting solution was added to the toluene solution of [La{N(SiMe_3_)_2_}_3_]. The combined mixture was stirred at room temperature for 18 h. The volatiles were removed *in vacuo* and the residue was triturated with pentane (5 mL), causing the formation of a white precipitate. The solid was filtered and dried *in vacuo*, affording **2-La** as a white solid (0.88 g, 1.3 mmol, 46%). ^1^H NMR (C_6_D_6_, 298 K, 400 MHz): δ/ppm = 1.04 (6 H, dd, ^3^*J*_HP_ = 12 Hz, ^3^*J*_HH_ = 7 Hz, CH(C*H*_3_)_2_), 1.18 (6 H, dd, ^3^*J*_HP_ = 15.5 Hz, ^3^*J*_HH_ = 7 Hz, CH(C*H*_3_)_2_), 2.08 (2 H, m, C*H*(CH_3_)_2_), 6.68 (1 H, t, ^3^*J*_HH_ = 7 Hz, Ar-C*H*), 6.80 (1 H, dd, ^3^*J*_HH_ = 8 Hz, ^3^*J*_HH_ = 8 Hz, Ar-C*H*), 7.12 (1 H, dd, ^3^*J*_HH_ = 7 Hz, ^4^*J*_HH_ = 2 Hz, Ar-C*H*), 7.25 (1 H, td, ^3^*J*_HH_ = 7 Hz, ^3^*J*_HP_ = 2 Hz, Ar-C*H*). ^1^H NMR (C_4_D_8_O, 298 K, 800 MHz): δ/ppm = 0.87 (6 H, dd, ^3^*J*_HP_ = 12 Hz, ^3^*J*_HH_ = 7 Hz, CH(C*H*_3_)_2_), 1.03 (6 H, dd, ^3^*J*_HP_ = 15 Hz, ^3^*J*_HH_ = 7 Hz, CH(C*H*_3_)_2_), 2.04 (2 H, m, C*H*(CH_3_)_2_), 6.33 (1 H, td, ^3^*J*_HH_ = 7 Hz, ^4^*J*_HH_ = 1 Hz, Ar-C*H*), 6.40 (1 H, dd, ^3^*J*_HH_ = 8 Hz, ^3^*J*_HH_ = 4 Hz, Ar-C*H*), 6.87 (1 H, m, Ar-C*H*), 6.97 (1 H, m, Ar-C*H*). ^13^C{^1^H} NMR (C_4_D_8_O, 298 K, 201.21 MHz): δ/ppm = 18.9 (d, ^2^*J*_CP_ = 7 Hz, CH(*C*H_3_)_2_), 19.7 (d, ^2^*J*_CP_ = 14 Hz, CH(*C*H_3_)_2_), 23.8 (d, ^2^*J*_CP_ = 4 Hz, *C*H(CH_3_)_2_), 114.8 (Ar-*C*H), 118.7 (Ar-*C*H), 120.9 (d, ^2^*J*_CP_ = 6 Hz, Ar-*C*H), 130.8 (Ar-*C*H), 132.4 (Ar-*C*-O), 173.2 (d, ^1^*J*_CP_ = 23 Hz, Ar-*C*-P). ^31^P{^1^H} NMR (C_6_D_6_, 298 K, 162 MHz): δ/ppm = 1.21. Anal. Calcd. (%) for C_36_H_54_O_3_P_3_La·C_5_H_12_: C 58.71, H 7.93. Found (%): C 59.57, H 7.69. FT-IR: $\tilde{v}$/cm^−1^ = 2953, 2927, 2889, 2868, 1575, 1455, 1431, 1289, 1261, 1248, 1220, 1153, 1121, 1031, 854.

### 5.6. Synthesis of [Sm{^t^Bu_2_P(C_6_H_4_)O}_3_] (***1-Sm***)

A flame-dried Schlenk flask was charged with [Sm{N(SiMe_3_)_2_}_3_] (0.684 g, 1.2 mmol) and 2-(di-*tert*-butylphosphino)phenol (0.86 g, 3.61 mmol). Toluene (60 mL) was added, and the reaction mixture was stirred at room temperature for 18 h. The volatiles were removed *in vacuo* and the residual solid was recrystallised from hexane (10 mL, room temperature), affording **1-Sm** as a white crystalline solid (0.075 g, 0.09 mmol, 7%). ^1^H NMR (C_4_D_8_O, 298 K, 400 MHz): δ/ppm = 0.28 (18 H, broad, ν_1/2_ = 7.45 Hz, C(C*H*_3_)_3_), 7.06 (1 H, t, ^3^*J*_HH_ = 7 Hz, Ar-C*H*), 7.56 (1 H, d. ^3^*J*_HH_ = 6 Hz, Ar-C*H*), 7.93 (1 H, t, ^3^*J*_HH_ = 6 Hz, Ar-C*H*), 9.67 (1 H, d, ^3^*J*_H-H_ = 6 Hz, Ar-C*H*). ^13^C{^1^H} (C_4_D_8_O, 298 K, 125.78 MHz): δ/ppm = 29.3 (C(*C*H_3_)_3_), 30.6 (*C*(CH_3_)_3_), 116.0 (Ar-*C*H), 119.9 (Ar-*C*H), 124.1 (Ar-*C*H), 132.4 (Ar-*C*H), 134.4 (Ar-*C*H), 181.3 (Ar-*C*-O). ^31^P{^1^H} (C_4_D_8_O, 298 K, 202.46 MHz): δ/ppm = −56.71 (ν_1/2_ = 1765.42 Hz). Anal. Calcd. (%) for C_42_H_66_O_3_P_3_Sm: C 58.5, H 7.71. Found (%): C 55.3, H 7.89; elemental analyses consistently yielded low carbon values, which we ascribed to carbide formation [47]. FT-IR: $\tilde{v}$/cm^−1^ = 2989, 2961, 2939, 2918, 2892, 2858, 1579, 1550, 1453, 1429, 1390, 1361, 1337, 1289, 1257, 1245, 1174, 1121, 1029.

### 5.7. Synthesis of [Y{^t^Bu_2_P(C_6_H_4_)O}_3_{^t^Bu_2_PH(C_6_H_4_)O}] (***3-Y***)

A flame-dried Schlenk flask was charged with [Y{N(SiMe_3_)_2_}_3_] (0.684 g, 1.2 mmol) and 2-(di-*tert*-butylphosphino)phenol (0.86 g, 3.61 mmol). Toluene (60 mL) was added, and the resulting solution was stirred at room temperature for 18 h. The volatiles were removed *in vacuo* and the residual solid was recrystallised from hexane (10 mL, −30 °C) to obtain the product as a white crystalline solid (0.099 g, 0.095 mmol, 4.8%). ^1^H NMR (C_6_D_6_, 298 K, 400 MHz): δ/ppm = 0.73–1.11 (18 H, broad, CH_3_), 1.18–1.64 (54 H, broad, CH_3_), 6.59–6.76 (4 H, broad, Ar-C*H*), 6.76–6.93 (4 H, broad, Ar-C*H*), 7.27 (4 H, t, ^3^*J*_HH_ = 7 Hz, Ar-C*H*), 7.52–7.73 (4 H, broad, Ar-C*H*), 7.42–8.27 (1 H, d, ^1^*J*_PH_ = 276 Hz, P*H*). ^13^C{^1^H} NMR (C_6_D_6_/C_4_D_8_O, 298 K, 100 MHz): 27.8 (*C*(CH_3_)_3_), 30.8 (PC(*C*H_3_)_3_), 33.3 (d, 7 Hz, HPC(*C*H_3_)_3_), 114.5 (Ar-*C*H), 120.0 (Ar-*C*H), 123.7 (Ar-*C*O), 131.0 (Ar-*C*H), 134.8 (Ar-*C*H), 172.2 (Ar-*C*P). ^31^P{^1^H} (C_6_D_6_, 298 K, 162 MHz): δ/ppm = 10.55 (*P*), 18.25 (*P*H). Anal. Calcd. (%) for C_56_H_89_O_4_P_4_Y·0.5(C_6_H_14_): C 65.48, H 8.94. Found (%) C: 65.93, H: 8.56. FT-IR: $\tilde{v}$/cm^−1^ = 2961, 2895, 2862, 1580, 1454, 1429, 1391, 1364, 1295, 1259, 1175, 1093, 1018, 857, 799.

### 5.8. Synthesis of [Ce{^t^Bu_2_P(C_6_H_4_)O}_3_{^t^Bu_2_PH(C_6_H_4_)O}] (***3-Ce***)

UPTOHERE A flame-dried Schlenk flask was charged with [Ce{N(SiMe_3_)_2_}_3_] (0.745 g, 1.2 mmol) and 2-(di-*tert*-butylphosphaneyl)phenol (0.858 g, 3.6 mmol). Toluene (40 mL) was added, and the mixture was stirred at room temperature for 18 h whilst protected from UV light. The volatiles were removed *in vacuo* and the residue was dissolved in hexane (2 mL). A precipitate crashed out at −30 °C, and the resulting suspension was filtered. The filtrate was transferred to a small vial in the glove box, where dark red crystals grew at room temperature by slow evaporation. The crystals were analysed by X-ray crystallography to confirm the structure of **3-Ce** (0.04 g, 0.03 mmol, 3.1%). ^1^H NMR (C_6_D_6_, 298 K, 400 MHz): δ/ppm = −4.46 (broad), −3.98 (broad), −3.42 (broad with shoulder), −2.30 (broad), −1.69 (broad), 0.16 (s), 1.33 (s), 3.58 (broad), 3.82 (broad), 5.09 (broad), 5.24 (broad), 5.70 (broad), 6.79 (broad), 6.96 (broad with shoulder), 7.05 (broad), 7.46 (broad), 7.71 (broad), 8.73 (broad), 9.01 (broad), 10.13 (broad), 10.55 (broad), 17.32 (broad). No ^13^C NMR data could be obtained due to paramagnetism. ^31^P{^1^H} (C_6_D_6_, 298 K, 162 MHz) δ/ppm = 40.54.

### 5.9. Synthesis of [Pr{^t^Bu_2_P(C_6_H_4_)O}_3_{^t^Bu_2_PH(C_6_H_4_)O}] (***3-Pr***)

A flame-dried Schlenk flask was charged with [Pr{N(SiMe_3_)_2_}_3_] (0.632 g, 1.01 mmol) and 2-(di-*tert*-butylphosphaneyl)phenol (0.727 g, 3.04 mmol). Toluene (50 mL) was added, and the mixture were stirred at room temperature for 18 h. The volatiles were removed *in vacuo* and the residual solid was recrystallised in hexane, affording **3-Pr** as a beige crystalline solid (0.193 g, 0.2 mmol, 23%). ^1^H NMR (C_6_D_6_, 298 K, 400 MHz): δ/ppm = −33.30 (broad, ν_1/2_ = 239.81 Hz), −6.37 (broad with shoulder, ν_1/2_ = 446.91 Hz), −5.8 (broad), −0.12 (s), −0.01 (s), 0.10 (s) 0.21 (s), 0.29 (s), 0.87–0.97 (broad m), 1.20–1.40 (broad m), 6.23 (broad, ν_1/2_ = 73.94 Hz), 6.96 (s), 7.36 (s), 12.10–16.62 (broad with shoulder), 96.18 (broad d, ^1^*J*_HP_ = 473.68 Hz, P*H*). No ^13^C and ^31^P NMR data could be obtained due to paramagnetism. Anal. Calcd. (%) C_56_H_89_O_4_P_4_Pr: C 61.64, H 8.22. Found (%) C 60.57, H 8.33; elemental analyses afforded low carbon values consistently, which we ascribe to carbide formation [47]. FT-IR: $\tilde{v}$/cm^−1^ = 2989, 2954, 2938, 2891, 2859, 1579, 1453, 1425, 1361, 1336, 1292, 1247, 1178, 1120, 1031, 933, 848.

### 5.10. Synthesis of [Sm{^t^Bu_2_P(C_6_H_4_)O}_3_{^t^Bu_2_PH(C_6_H_4_)O}] (***3-Sm***)

A flame-dried Schlenk flask was charged with [Sm{N(SiMe_3_)_2_}_3_] (1.571 g, 2.49 mmol) and 2-(di-*tert*-butylphosphaneyl)phenol (1.787 g, 7.5 mmol). Toluene (40 mL) was added and the mixture stirred at room temperature for 18 h. The volatiles were removed *in vacuo*, and the residual solid was recrystallised in a mixture of hexane and toluene, affording obtain **3-Sm** as a white crystalline solid (0.096 g, 0.09 mmol, 3%). Notably, **1-Sm** was also isolated from the same reaction upon fractional crystallisation (*vide supra*). ^1^H NMR (C_6_D_6_, 298 K, 400 MHz): δ/ppm = 0.62 (54 H, broad, C(C*H*_3_)_3_), 1.96 (18 H, broad, C(C*H*_3_)_3_), 6.30 (4 H, broad, Ar-C*H*), 7.64 (4 H, broad, Ar-C*H*), 7.79 (4 H, broad, Ar-C*H*), 8.64 (4 H, broad, Ar-C*H*), 14.72 (1 H, broad, P*H*), 15.94 (1 H, broad s, P*H*). ^31^P{^1^H} (C_6_D_6_, 298 K, 162 MHz): δ/ppm = −44.34 (broad, ν_1/2_ 384.04 Hz), 21.95 (broad, ν_1/2_ 470.76 Hz). No ^13^C NMR data could be obtained due to paramagnetism. Anal. Calcd. (%) for C_56_H_89_O_4_P_4_Sm: C 61.11, H 8.15. Found (%) C 61.54, H 8.24. FT-IR: $\tilde{v}$/cm^−1^ = 2956, 2927, 2865, 1465, 1448, 1383, 1366, 1336, 1324, 1259, 1252, 1239, 1207, 1176, 1159, 1144, 1108, 1052, 1039, 976, 958, 939, 920, 897, 885, 876, 813, 806.

### 5.11. Synthesis of K[^t^Bu_2_P(C_6_H_4_)O]

2-di-*tert*-butylphosphinophenol (1.906 g, 8 mmol) and KH (0.326 g, 8.3 mmol) were added to a flame-dried Schlenk flask. THF (25 mL) was added, and the resulting suspension was stirred at room temperature for 18 h. The suspension was filtered and the filtrate was dried *in vacuo*, affording K[^t^Bu_2_P(C_6_H_4_)O] as a white powder (2.096 g, 7.6 mmol, 95%). ^1^H NMR (C_6_D_6_, 298 K, 400 MHz): δ/ppm = 1.30 (18 H, d, ^3^*J*_HP_ = 12 Hz, C(C*H*_3_)_3_), 6.29 (1 H, t, ^3^*J*_HH_ = 6 Hz, Ar-C*H*), 6.65 (1 H, m, Ar-C*H*), 7.26 (1 H, m, Ar-C*H*), 7.69 (1 H, m, Ar-C*H*). ^13^C{^1^H} NMR (C_6_D_6_/C_4_D_8_O, 298 K, 100 MHz): δ/ppm = 31.5 (d, ^2^*J*_PC_ = 15 Hz, C(*C*H_3_)_3_), 32.8 (d, ^1^*J*_PC_ = 18 Hz, *C*(CH_3_)_3_), 111.5 (Ar-*C*H), 118.8 (Ar-*C*-O), 123.1 (d, ^2^*J*_CP_ = 6 Hz, Ar-*C*H), 131.9 (Ar-*C*H), 137.4 (Ar-*C*H), 175.4 (d, ^1^*J*_CP_ = 22 Hz, Ar-*C*-P). ^31^P{^1^H} NMR (C_6_D_6_, 298 K, 162 MHz): δ/ppm = 9.03. Anal. Calcd. (%) for C_14_H_22_OPK: C 60.84, H 8.02. Found (%): C 60.15, H 8.30.

### 5.12. Synthesis of [La{^t^Bu_2_P(C_6_H_4_)O}_6_K_3_] (***4***)

A flame-dried Schlenk flask was charged with LaI_3_(THF)_4_ (0.664 g, 1 mmol) and K[^t^Bu_2_P(C_6_H_4_)O] (0.937 g, 3 mmol). THF (40 mL) was added, and the mixture was stirred at room temperature for 18 h. The volatiles were removed *in vacuo* and the residue was washed with hexane (3 × 5 mL). The filtrate was concentrated *in vacuo* to about 8 mL. From the filtrate, two crystalline solids precipitated out. The pale red crystals were identified as **3-La** via X-ray crystallography. The clear colourless crystals were identified as **4** via X-ray crystallography (0.015 g, 0.01 mmol, 1%). ^1^H NMR (C_6_D_6_/C_4_D_8_O, 298 K, 400 MHz): δ/ppm = 1.26 (108 H, broad, C(C*H*_3_)_3_), 6.62 (8 H, broad, Ar-C*H*), 7.03 (8 H, broad, Ar-C*H*), 7.20 (8 H, broad, Ar-C*H*), 7.53 (8 H, broad, Ar-C*H*). ^13^C{^1^H} NMR (C_6_D_6_/C_4_D_8_O, 298 K, 100 MHz): δ/ppm = 31.0 (d, ^2^*J*_PC_ = 12 Hz, C(*C*H_3_)_3_), 33.2 (d, ^1^*J*_PC_ = 13 Hz, *C*(CH_3_)_3_), 114.5 (Ar-*C*H), 120.9 (Ar-*C*H), 122.9 (Ar-*C*-O), 130.8 (Ar-*C*H), 135.5 (Ar-*C*H), 173.2 (Ar-*C*-P). ^31^P{^1^H} NMR (C_6_D_6_, 298 K, 162 MHz): δ/ppm = 13.36.

### 5.13. General Procedure for Gas Reactions—CO and CO_2_

The metal complex was added to a flame-dried Schlenk flask. Toluene was added, together with additional diethyl ether if needed to dissolve all solids. The solution was transferred to an ampoule fitted with a Rotaflo valve. The solution was degassed (freeze-pump-thaw) and then refilled with an atmosphere of the chosen gas (~0.5 bar, CO or CO_2_). The solution was stirred at room temperature (18–72 h). An aliquot of the solution was transferred to a flame-dried Schlenk flask and analysed by NMR spectroscopy to monitor reaction progress (^1^H, ^13^C, ^31^P, ^31^P{^1^H}).

### 5.14. Reaction Between a Mixture of ***1-La*** and ***3-La*** with CO_2_ (Isolation of ***3-La***)

A mixture of **1-La** and **3-La** (0.299 g) was added to a flame-dried Schlenk flask. The flask was charged with toluene (15 mL) and diethyl ether (1 mL) to dissolve all solids. The solution was transferred to a Rotaflo ampoule. The solution was frozen, degassed and then refilled with an atmosphere of CO. The solution was thawed and stirred (64 h). An aliquot of the solution was transferred to a flame-dried Schlenk flask and analysed by NMR spectroscopy (^1^H, ^13^C, ^31^P, ^31^P{^1^H}), proving that no reaction had taken place. The contents of the flask were filtered, and the filtrate was concentrated to around 10 mL of solvent. Compound **3-La** was recrystallised from the filtrate at −30 °C in toluene. The solid was filtered and dried in, to yield **3-La** as an off-white solid (0.074 g). ^1^H NMR (C_6_D_6_, 298 K, 400 MHz): δ/ppm = 0.9–1.7 (72 H, broad, C(C*H*_3_)_3_), 6.42–6.72 (4 H, broad, Ar-C*H*), 6.75–7.00 (4 H, broad, Ar-C*H*), 7.32 (4 H, t, ^3^*J*_HH_ = 8 Hz, Ar-C*H*), 7.28–7.76 (4 H, broad, Ar-C*H*), 8.40 (1 H, broad, P*H*). ^13^C{^1^H} NMR (C_6_D_6_, 298 K, 100 MHz): δ/ppm = 30.7 (broad with shoulder, PC(*C*H_3_)_3_), 33.7 (HPC(*C*H_3_)_3_), 114.3 (Ar-*C*H), 119.5 (Ar-*C*H), 123.8 (Ar-*C*P), 131.2 (s, Ar-*C*H), 134.8 (s, Ar-*C*H), 173.9 (s, Ar-*C*O). ^31^P{^1^H} (C_6_D_6_, 298K, 162 MHz): δ/ppm = 18.7. Anal. Calcd. (%) for C_56_H_89_O_4_P_4_La: C 61.76, H 8.24. Found (%) C 61.45, H 8.30. FT-IR: $\tilde{v}$/cm^−1^ = 2991, 2960, 2947, 2934, 2887, 2859, 1578, 1452, 1428, 1336, 1293, 1247, 1121, 1031, 848.

*Reaction between a mixture of* **1-La** *and* **3-La** *with CO:* In a similar procedure to the reactivity of a mixture of **1-La** and **3-La** with CO_2_, no reaction was observed, and **3-La** was isolated.

*Reaction between* **2-La** *and CO:* **2-La** (0.3 g) in toluene (8 mL) and diethyl ether (42 mL), CO (~0.5 bar). NMR spectroscopic analysis (C_6_D_6_, ^1^H, ^13^C, ^31^P, ^31^P{^1^H}) on an aliquot from the reaction after 64 h showed no reaction. The solution was concentrated *in vacuo* to 4 mL affording a crystalline solid upon standing at room temperature, which was analysed by X-ray crystallography to confirm the identity of **2-La**. The crystals were dried *in vacuo* to yield **2-La** (0.22 g recovered).

*Reaction between* **2-La** *and CO_2_:* (0.3 g) in toluene (8 mL) and diethyl ether (42 mL), CO_2_ (~0.5 bar) In a procedure similar to the reactivity of **2-La** with CO, no reaction was observed and **2-La** was retrieved.

### 5.15. General Procedure for Reactivity with H_2_

The metal complex was added to an oven-dried J. Young’s NMR tube, which was then charged with C_6_D_6_ (0.5 mL) and drops of THF-d_8_ as required. After obtaining NMR spectra of the starting material (^1^H, ^13^C, ^31^P, ^31^P{^1^H}), the solution was frozen using liquid N_2_ and the headspace was evacuated and backfilled with H_2_ gas. The solution was thawed and, after 3 h, analysed by NMR spectroscopy. The NMR tube was heated to 80 °C for 24 h and then analysed once again by NMR spectroscopy.

*Reaction between* **2-La** *and H_2_:* **2-La** (0.014 g, 0.0226 mmol) in C_6_D_6_ (0.5 mL) and 7 drops of THF-d_8_. No reaction observed.

*Reaction between* **3-Y** *and H_2_:* (0.014 g, 0.013 mmol) In a similar procedure to the previous one, no reactivity was observed.

*Reaction between LaN(SiMe_3_)_2_}_3_ and 2-di-tert-butylphosphinophenol:* La{N(SiMe_3_)_2_}_3_ (0.0102 g, 0.016 mmol) and 2-di-*tert*-butylphosphinophenol (0.0155 g, 0.065 mmol) was added to a J. Young’s NMR tube and dissolved in C_6_D_6_ (0.6 mL). NMR spectra were recorded after 30 min. NMR data analysis confirmed the formation of **3-La** as the major product, the formation of **1-La** as a minor product and the formation of other unknown minor impurities.

*Synthesis of {^t^Bu_2_PH(C_6_H_4_)O}B(C_6_F_5_)_3_* (**5**): 2-di-tert-butylphosphinophenol (0.005 g, 0.02 mmol) and B(C_6_F_5_) (0.010 g, 0.02 mmol) were added to a J. Young’s NMR tube and dissolved in C_6_D_6_ (0.6 mL). NMR spectra were recorded after 45 min. NMR data analysis confirmed quantitative formation of **5**.

**5:** ^1^H (C_6_D_6_, 298 K, 400 MHz): δ/ppm = 0.65 (18 H, d, ^2^*J*_PH_ = 17 Hz, P-C(C*H*_3_)_3_), 6.33 (1 H, td, ^3^*J*_HH_ = 7.4 Hz, ^4^*J*_HH_ = 3 Hz, Ar-C*H*), 6.39 (1 H, d, ^1^*J*_PH_ = 480 Hz, P*H*), 6.59 (1 H, m, Ar-C*H*), 6.92 (1 H, td, ^3^*J*_HH_ = 8 Hz, ^4^*J*_HH_ = 1.28 Hz, Ar-C*H*), 7.18 (1 H, m, Ar-C*H*). ^11^B (C_6_D_6_, 298 K, 128 MHz): δ/ppm = −2.78. ^19^F (C_6_D_6_, 298 K, 376 MHz): δ/ppm = −164.94 (*m*-*F*), −159.06 (*p*-*F*), −133.66 (*p*-*F*). ^31^P{^1^H} (C_6_D_6_, 298K, 162 MHz): δ/ppm = 23.63 (^1^*J*_PH_, *P*H).

*Reaction between* **3-Y** *and B(C_6_F_5_)_3_ and formation of* **1-Y** *and* **5**: **1-Y** (0.010 g, 0.01 mmol) and B(C_6_F_5_) (0.005 g, 0.01 mmol) were added to a J. Young’s NMR tube and dissolved in C_6_D_6_ (0.6 mL). NMR spectra were recorded after 45 min. NMR data analysis confirmed the formation of **1-Y** and **5**.

*Reaction between* **3-La** *and B(C_6_F_5_)_3_ and formation of* **5**: **1-La** (0.010 g, 0.01 mmol) and B(C_6_F_5_) (0.005 g, 0.01 mmol) were added to a J. Young’s NMR tube and dissolved in C_6_D_6_ (0.6 mL). NMR spectra were recorded after 60 min. NMR data analysis confirmed formation of **1-La** and **5**, together with unknown impurities.

## Figures and Tables

**Table 1:** Selected Bond Lengths for **1-RE** (RE = La, Sm) and **2-La**.

Complex	M–P	M···P	M–O	M–µO
**1-La**	3.223(2)–3.459(3)	3.662(2)–4.701(2)	2.235(6)–2.250(6)	2.418(6)–2.526(5)
**2-La**	3.1653(10)–3.3466(7)	3.4732(7)	2.255(2)–2.297(2)	2.458(2)–2.477(2)
1-Sm *	3.0552(8)–3.1503(8)	-	2.184(2)–2.205(3)	-

* Data extracted from one of the polymorphs isolated.

**Table 2:** Selected Bond Lengths for **3-RE** (RE = Y, La, Pr, Sm).

Complex	M–P	M···P	M···PH	M–O
3-Y *	3.0963(11)–3.1926(10)	3.627(3)–4.5242(9)	4.093(2)–4.2902(14)	2.122(2)–2.184(2)
**3-La**	3.2937(6)–3.3646(8)		4.3034(8)	2.303(2)–3.351(2)
**3-Pr ^§^**	3.2984(12)–3.3849(14)	3.451(5)	4.040(6)	2.232(5)–2.357(9)
**3-Sm ^#^**	3.2328(13)–3.3483(12)	-	4.2693(13)	2.206(3)–2.269(3)

* Two independent molecules present in the asymmetric unit. ^§^ Data extracted from major disorder component. ^#^ Data extracted from one of the polymorphs isolated.

## Data Availability

Additional research data supporting this publication are available from Figshare at 10.25392/leicester.data.27325713.

## Supplementary Materials

The following supporting information can be downloaded at: https://www.mdpi.com/article/10.3390/molecules29235757/s1. Additional experimental details and spectroscopic data (multinuclear NMR, IR), crystallographic data for all compounds [48,49,50,51].
