# Supplementary material for: Synthesis and Characterisation of Phosphino-Aryloxide Rare Earth Complexes
Source: Molecules. 2024 Dec 5;29(23):5757. doi: 10.3390/molecules29235757 (PMC11643060; doi:10.3390/molecules29235757)
Supplement: Supplementary file 1 [file molecules-29-05757-s001.zip › molecules-3327316-supplementary.pdf]

Synthesis and Characterisation of Phosphino-aryloxide Rare Earth Complexes

Elias Alexopoulos,[a] Yu Liu,[a] Alex W. J. Bowles,[a] Benjamin L. L. Réant[b]* and Fabrizio Ortu[a]*

[a]School of Chemistry, University of Leicester, University Road, Leicester, LE1 7RH, UK

[b]Department of Chemistry, The University of Manchester, Oxford Road, Manchester, M13 9PL, UK

*fabrizio.ortu@leicester.ac.uk; benjamin.reant@manchester.ac.uk

Table of Contents

S1. NMR data S2

S2. IR data S29

S3. Crystallography S34

S4. References S39

S1. NMR data

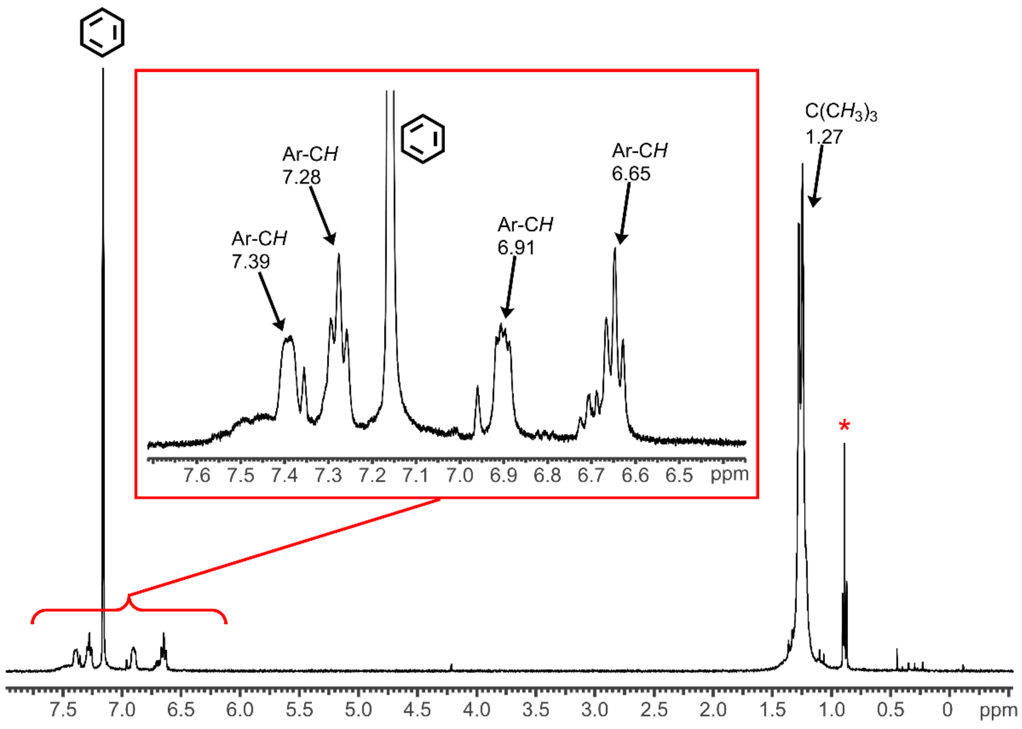

Figure S1A: ^{1}H NMR spectrum of **1-La** (C_6D_6, 298 K, 400 MHz). Inset shows aromatic region (6.5-7.6 ppm). * denotes solvent impurities (hexane, crystallisation solvent).

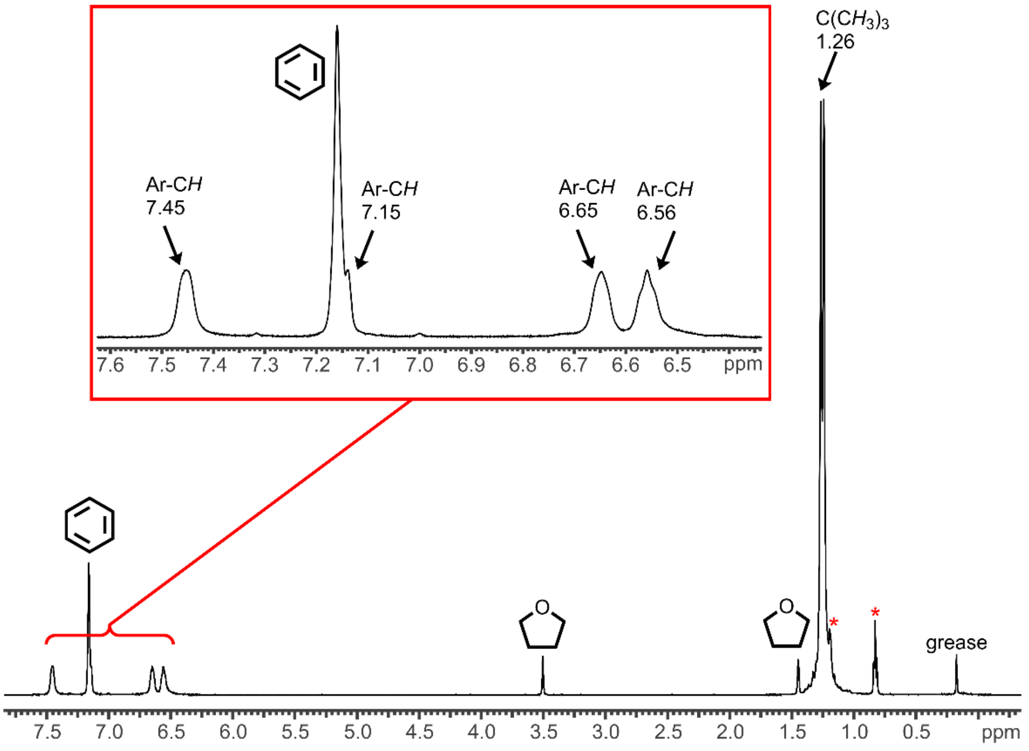

Figure S1B: ^{1}H NMR Spectrum of **1-La** (C_4D_8O, 298K, 500 MHz). Inset shows aromatic region (6-7.6 ppm). * denotes solvent impurities (hexane, crystallisation solvent).

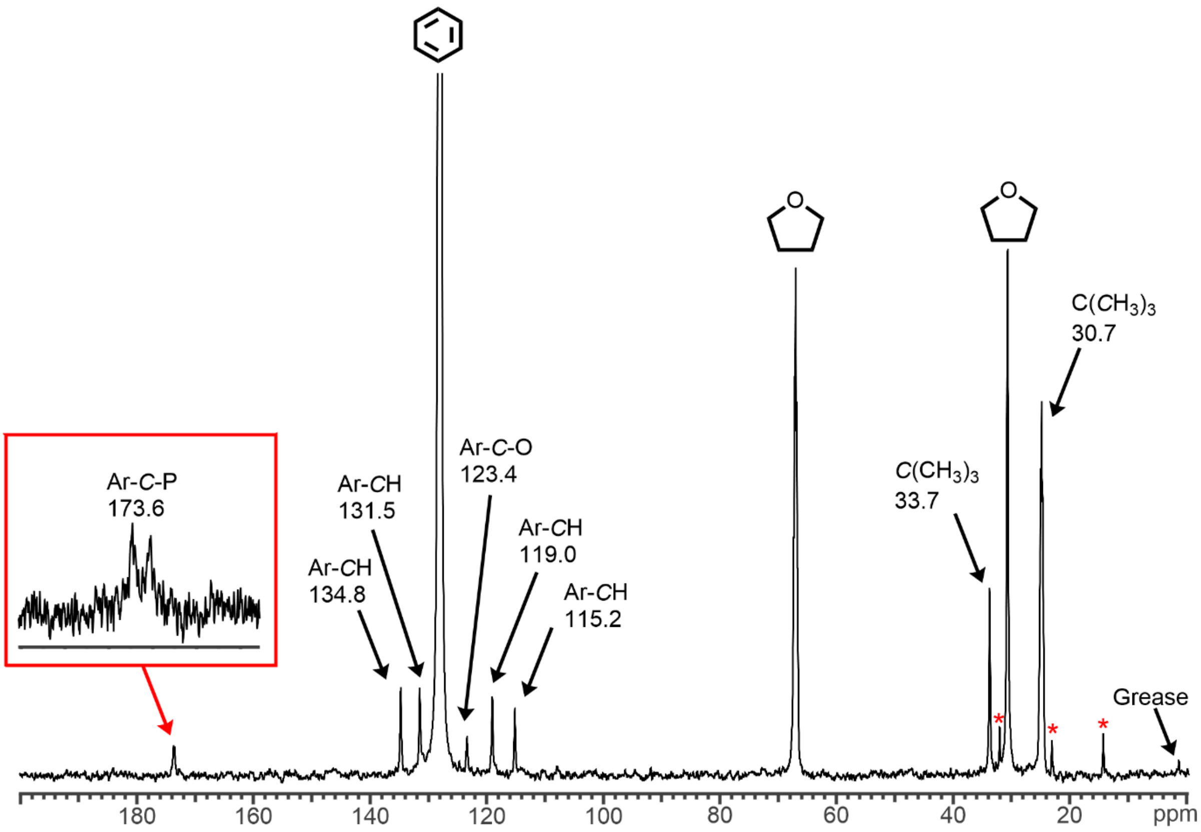

Figure S2 $^{13}C\{^1H\}$ NMR spectrum of **1-La** (C_6D_6/C_4D_8O, 298 K, 125.78 MHz). Inset shows doublet at 173.6 ppm. * denotes solvent impurities (hexane, crystallisation solvent).

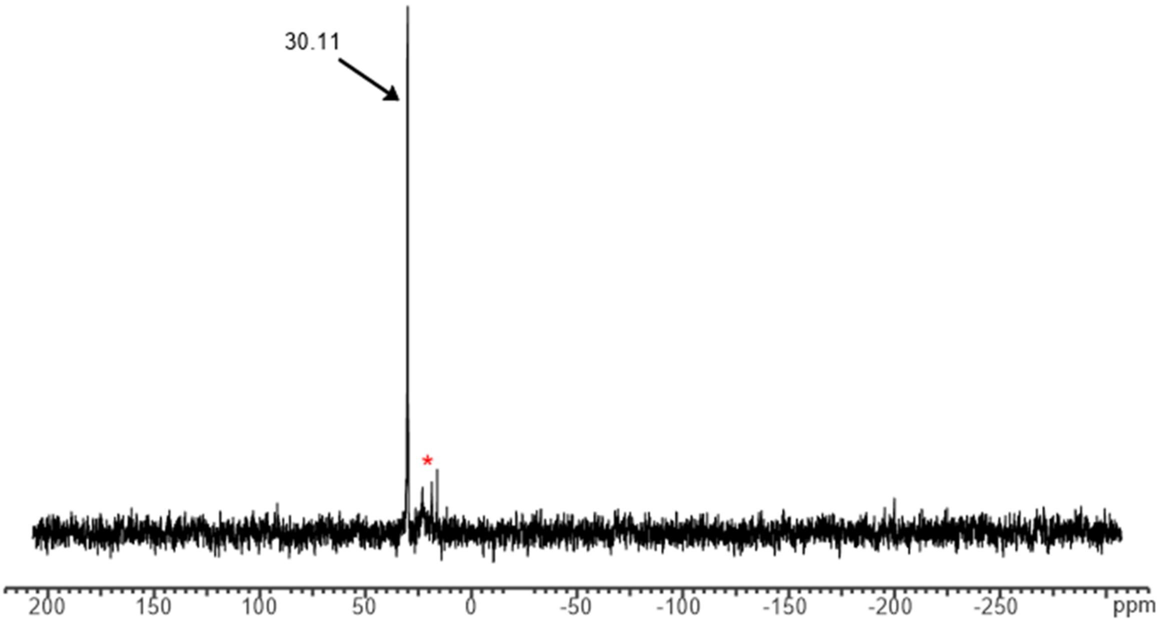

Figure S3A: $^{31}P\{^1H\}$ NMR spectrum of **1-La** (C_6D_6, 298 K, 162 MHz). * denotes **3-La** (minor impurity).

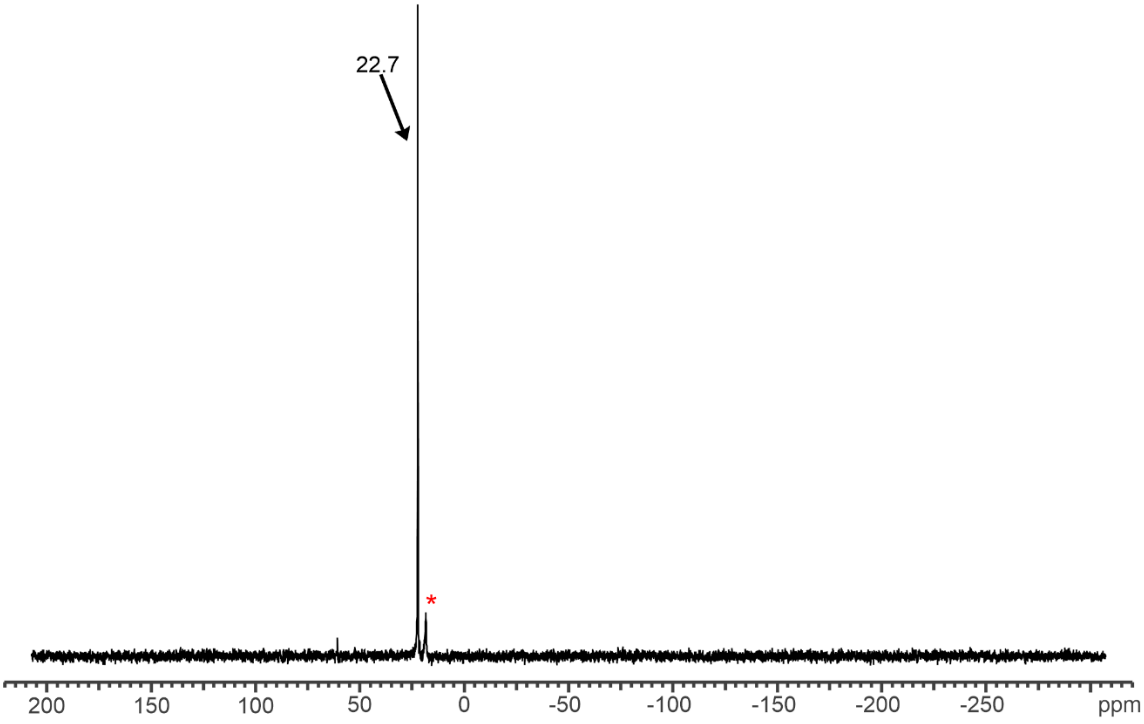

Figure S3B: $^{31}P\{H\}$ NMR Spectrum of **1-La** (C_6D_6/C_4D_8O, 298 K, 202.45 MHz). * denotes **3-La** (minor impurity).

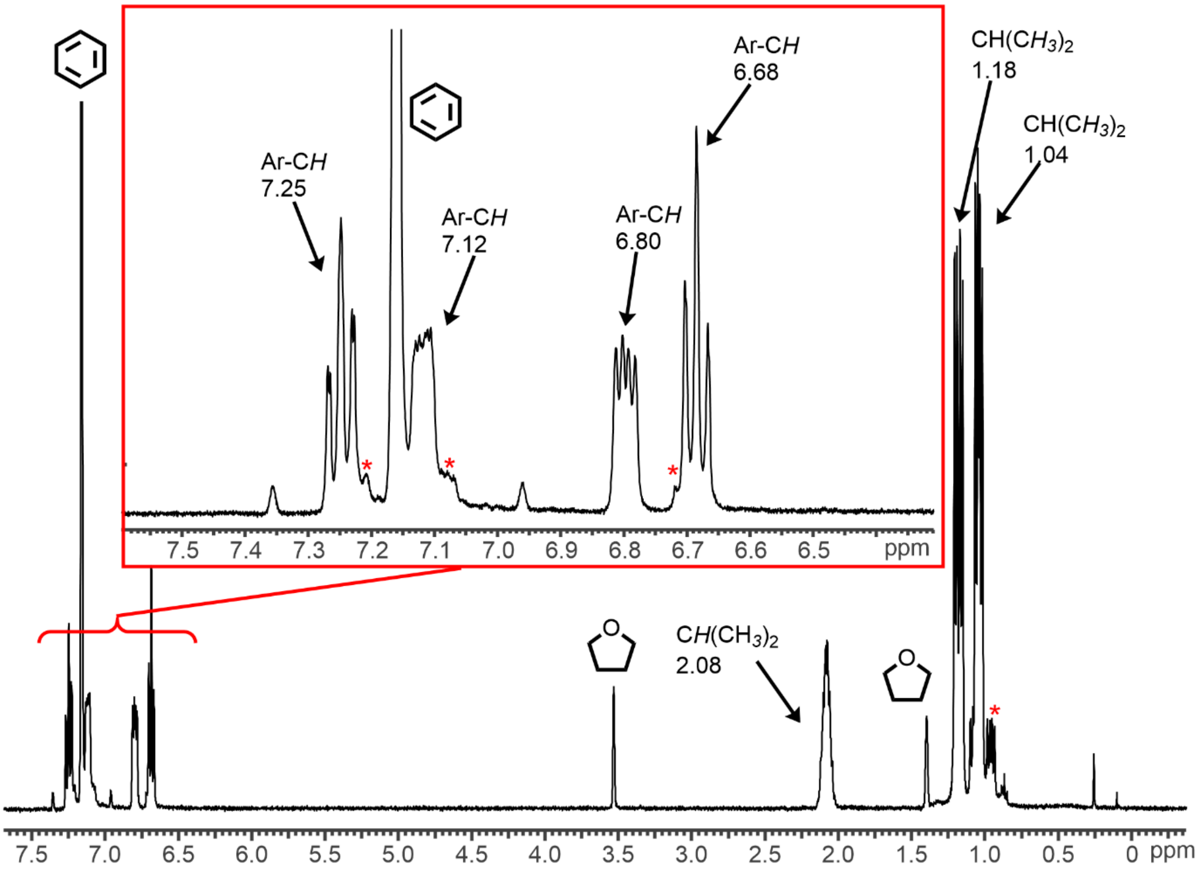

Figure S4A: ^{1}H NMR spectrum of **2-La** (C_6D_6/C_4D_8O, 298 K, 400 MHz).* denotes additional minor impurities which could not be identified.

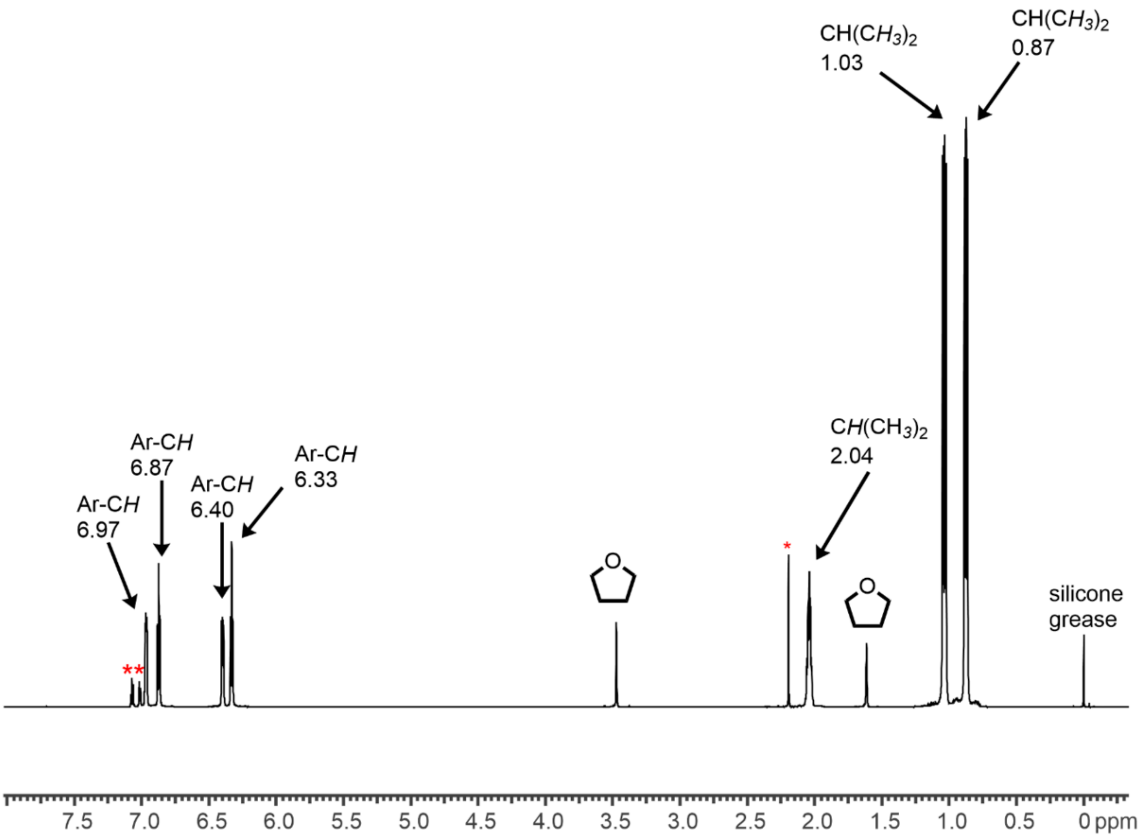

Figure S4B: ^{1}H NMR spectrum of **2-La** (C_4D_8O, 298 K, 800 MHz).* denotes solvent impurities (toluene).

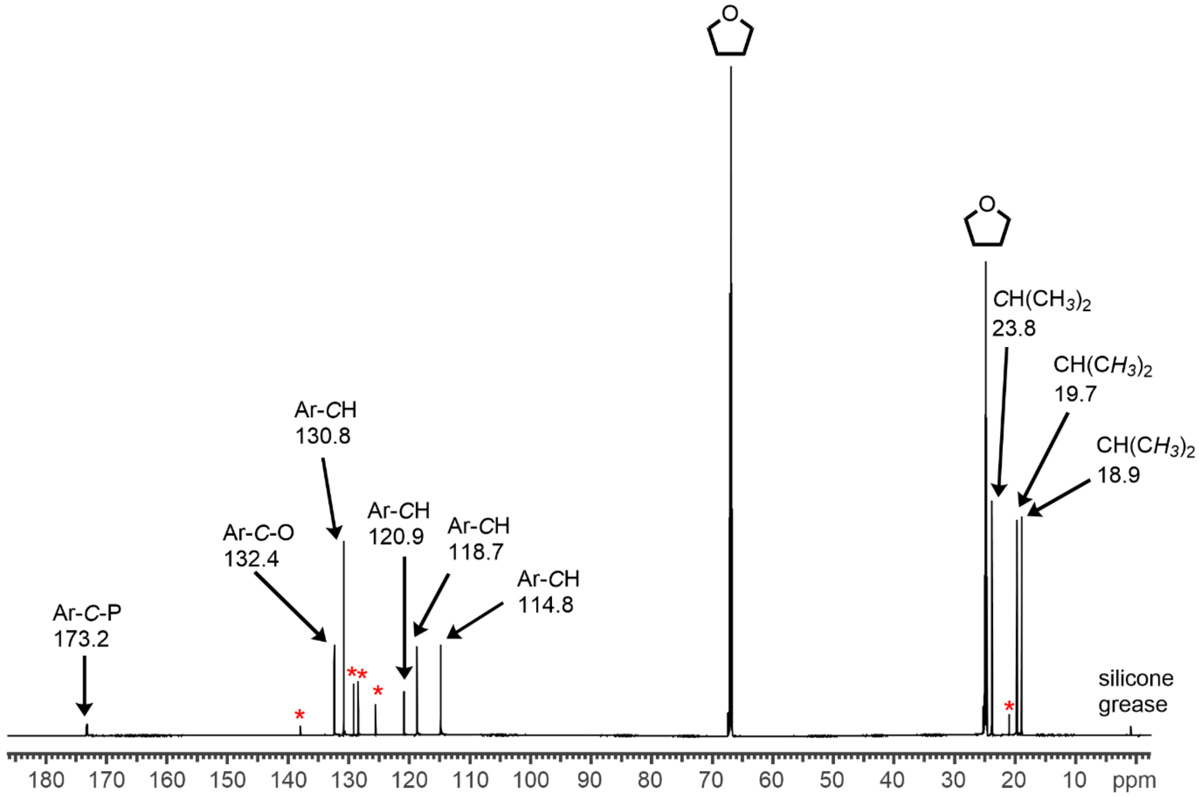

Figure S5: ^{13}C{^{1}H} NMR Spectrum of **2-La** (C_4D_8O, 298 K, 201 MHz). * denotes solvent impurities (toluene).

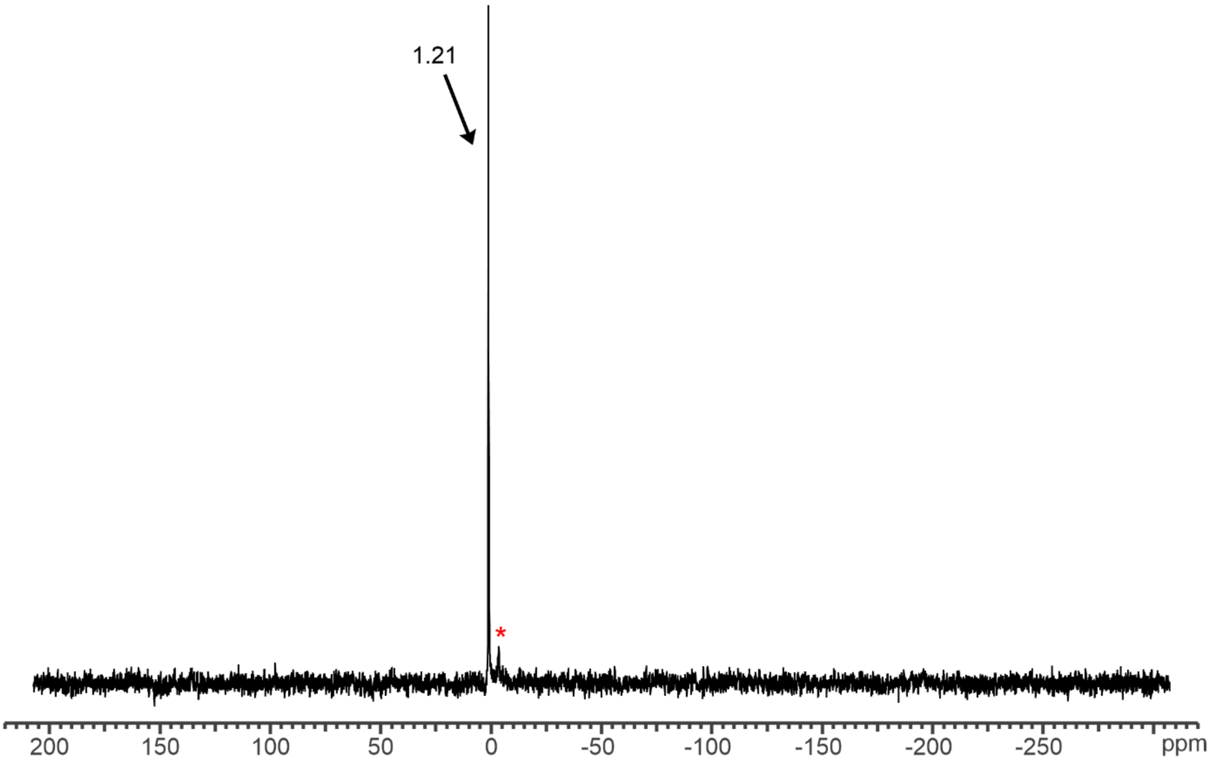

Figure S6: $^{31}P\{^{1}H\}$ NMR Spectrum of **2-La** (C_6D_6/C_4D_8O, 298 K, 162 MHz). * denotes an unidentified impurity (see also Figure S4A).

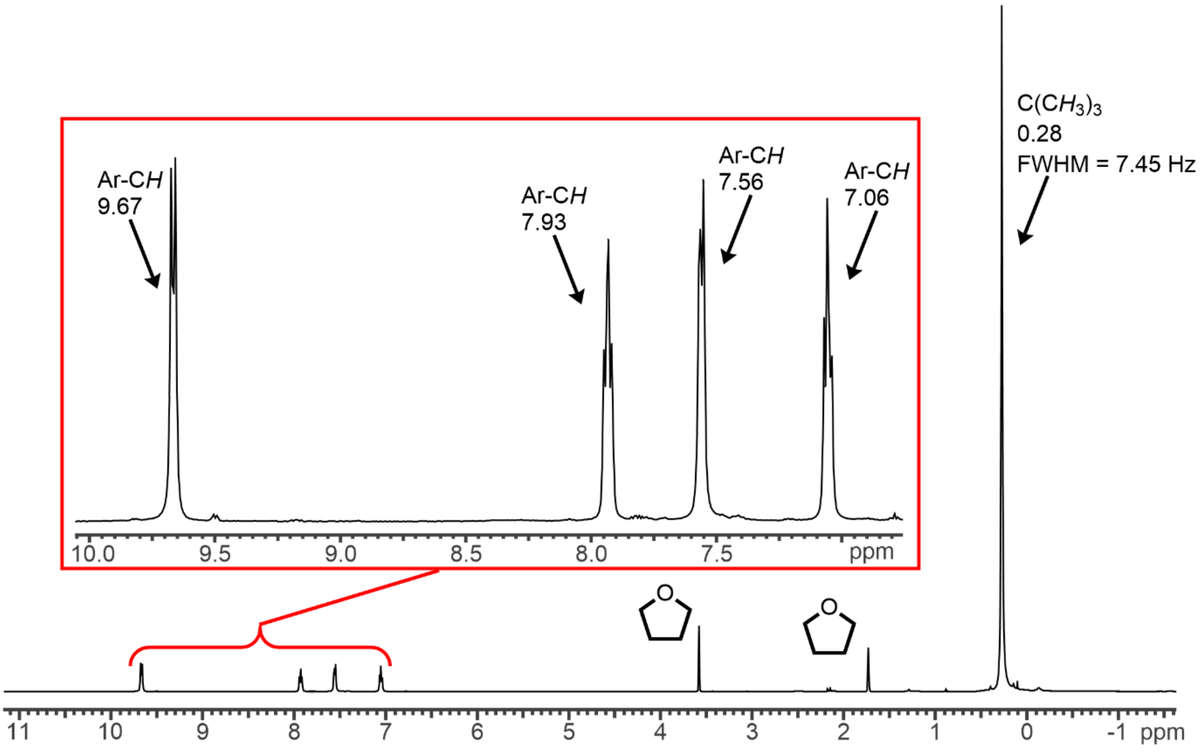

Figure S7: ^{1}H NMR spectrum of **1-Sm** (C_4D_8O, 298 K, 500 MHz). Inset shows aromatic region (7-10 ppm).

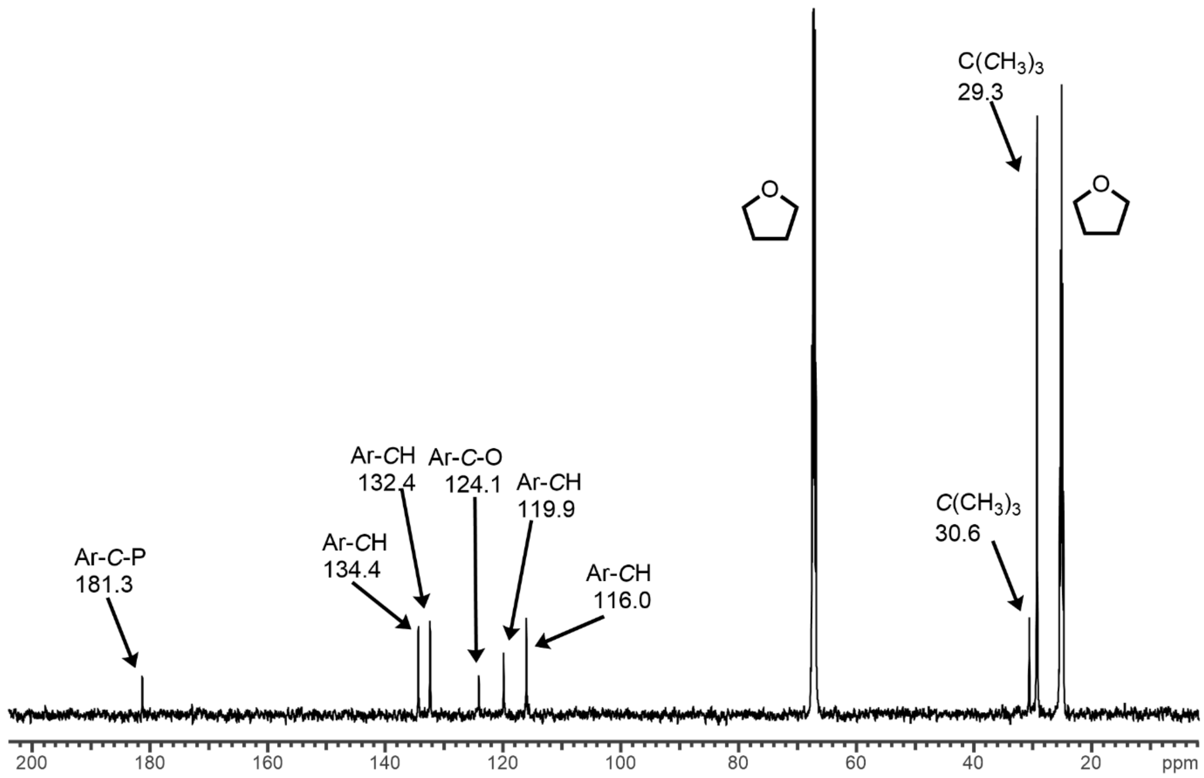

Figure S8: $^{13}C\{^{1}H\}$ NMR spectrum of **1-Sm** (C_4D_8O, 298 K, 125.78 MHz).

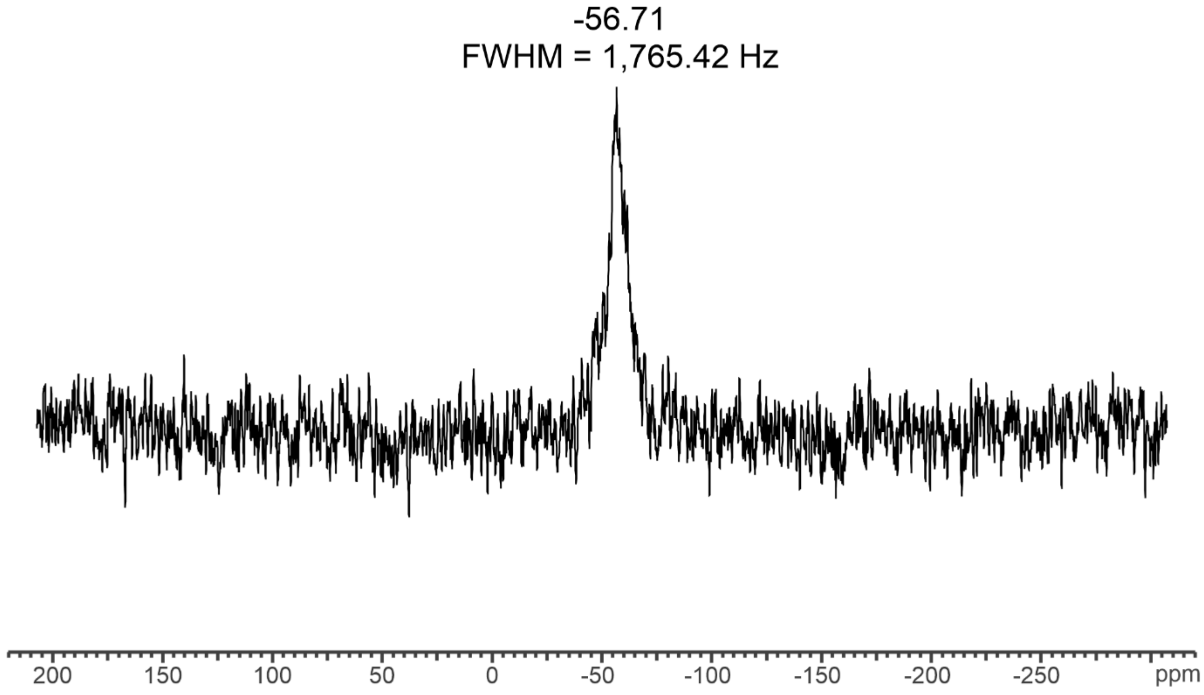

Figure S9: $^{31}P\{H\}$ NMR Spectrum of **1-Sm** (C_4H_8O, 298 K, 202.45 MHz).

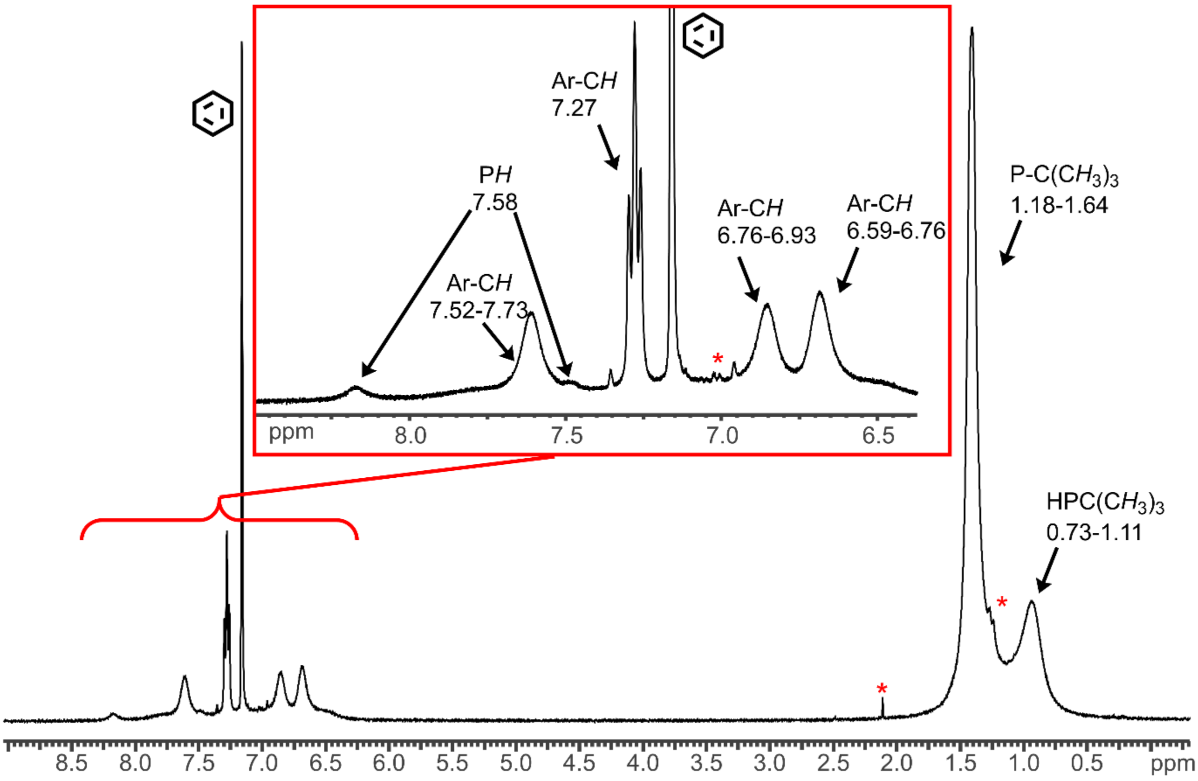

Figure S10: ^{1}H NMR Spectrum of **3-Y** (C_6D_6, 298 K, 400 MHz). * denotes solvent impurities (toluene, hexane).

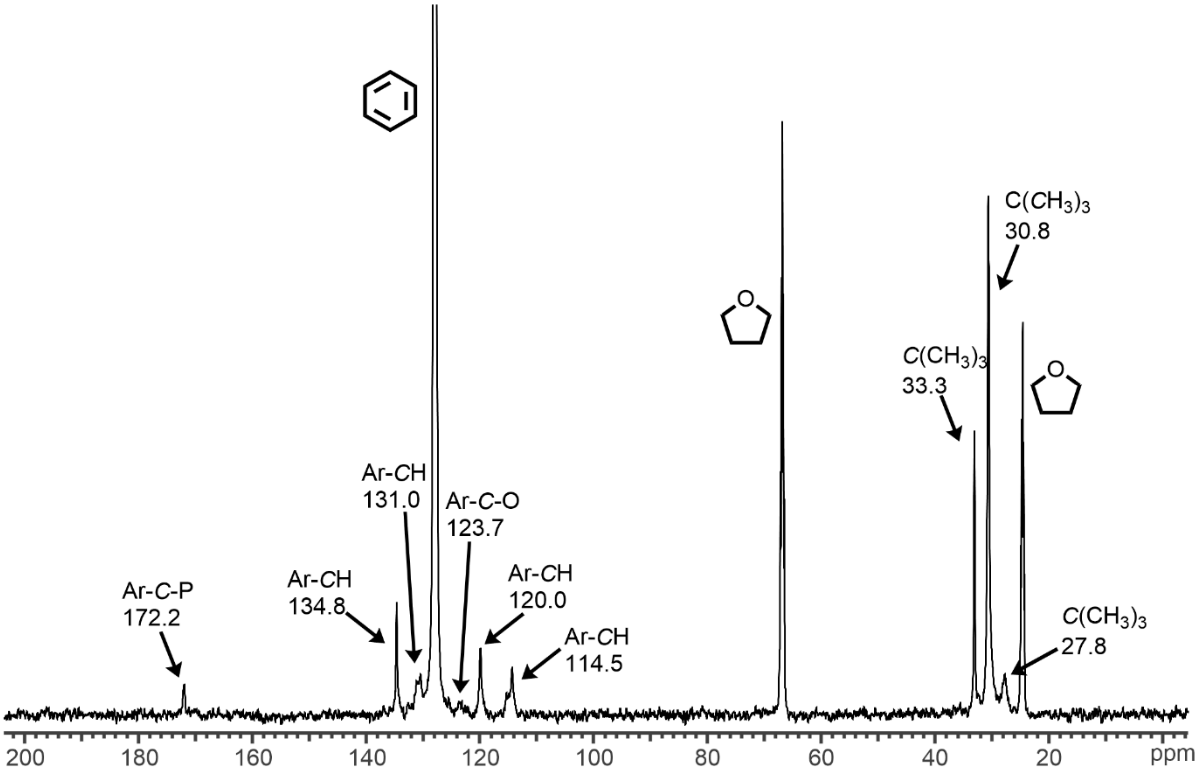

Figure S11: $^{13}C\{^{1}H\}$ NMR spectrum of **3-Y** (C_6D_6/C_4D_8O, 298 K, 125.78 MHz).

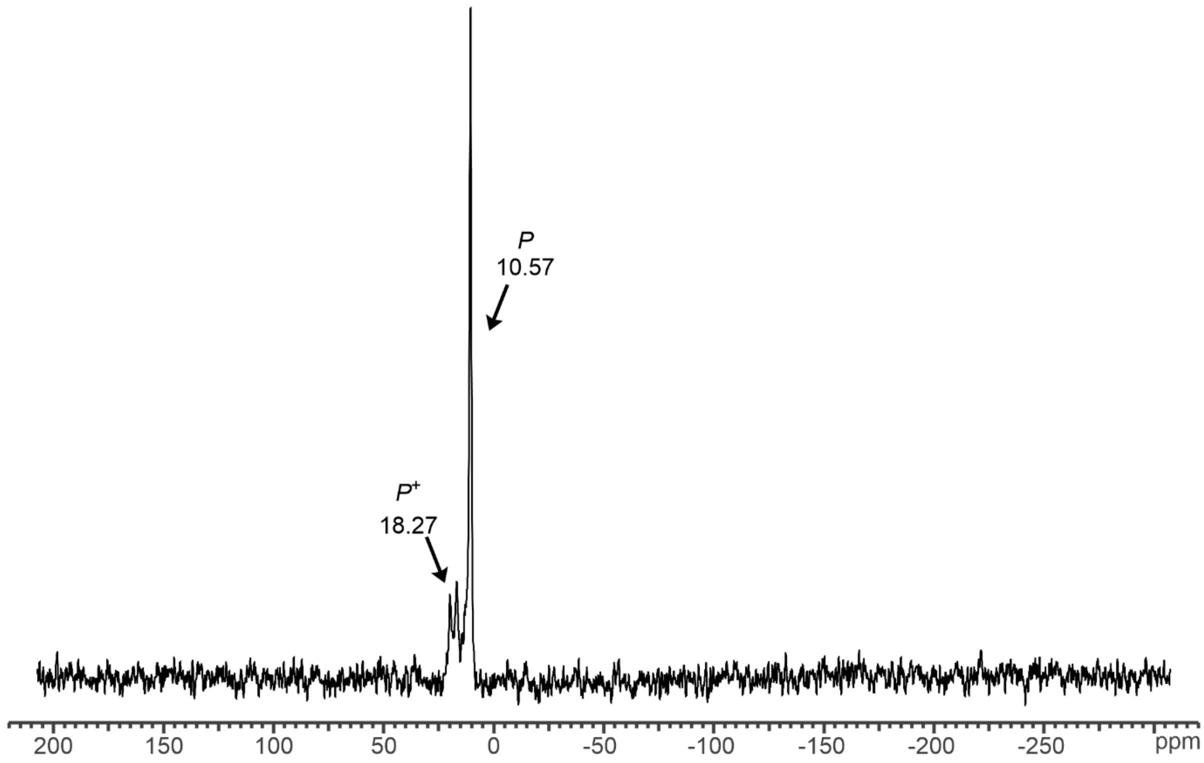

Figure S12: ^{31}P NMR spectrum of **3-Y** (C_6D_6, 298 K, 161.97 MHz).

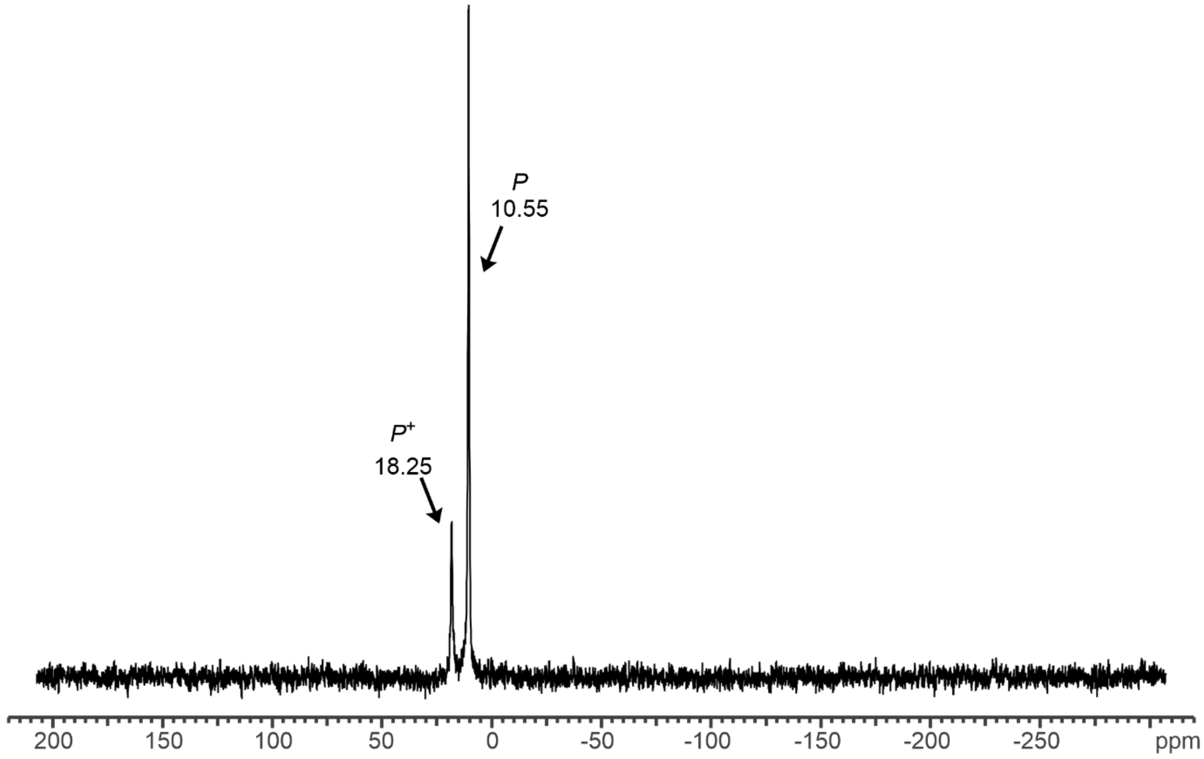

Figure S13: $^{31}P\{H\}$ NMR Spectrum of **3-Y** (C_6D_6, 298 K, 161.97 MHz).

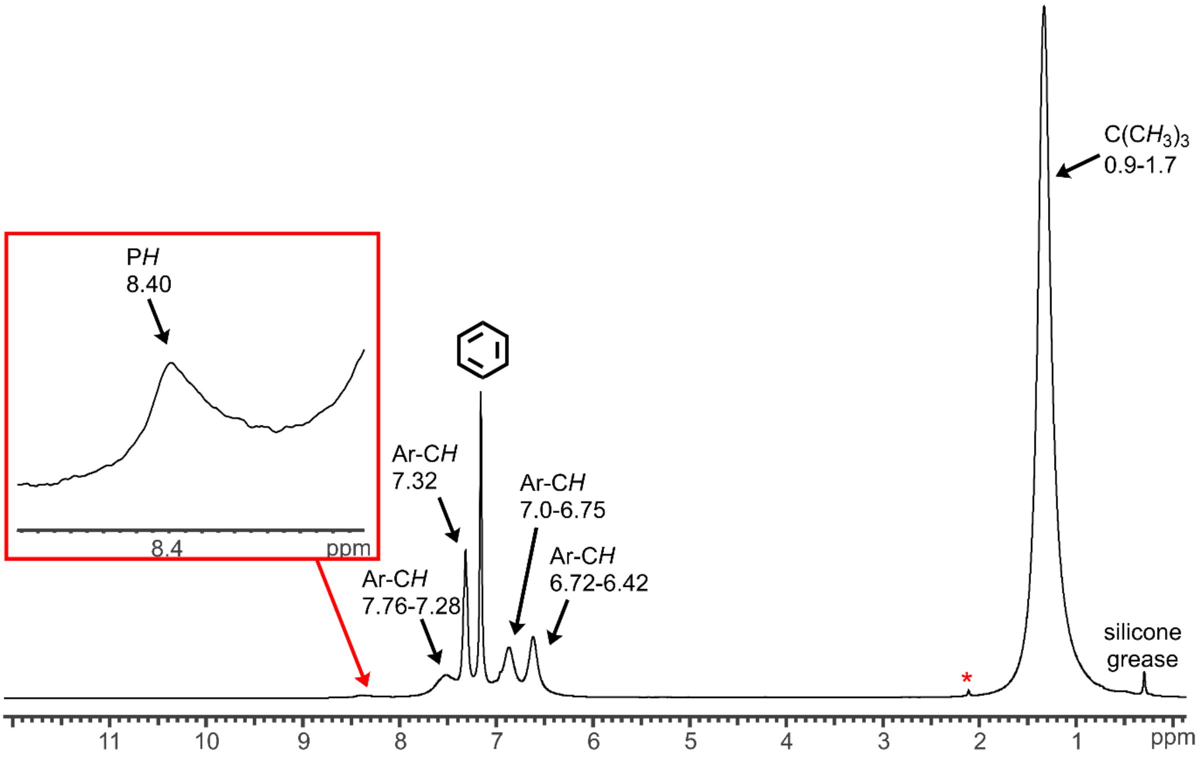

Figure S14: ^{1}H NMR spectrum of **3-La** (C_6D_6, 298 K, 400 MHz). Inset shows signal of phosphonium proton (~8.4 ppm). * denotes solvent impurities (toluene).

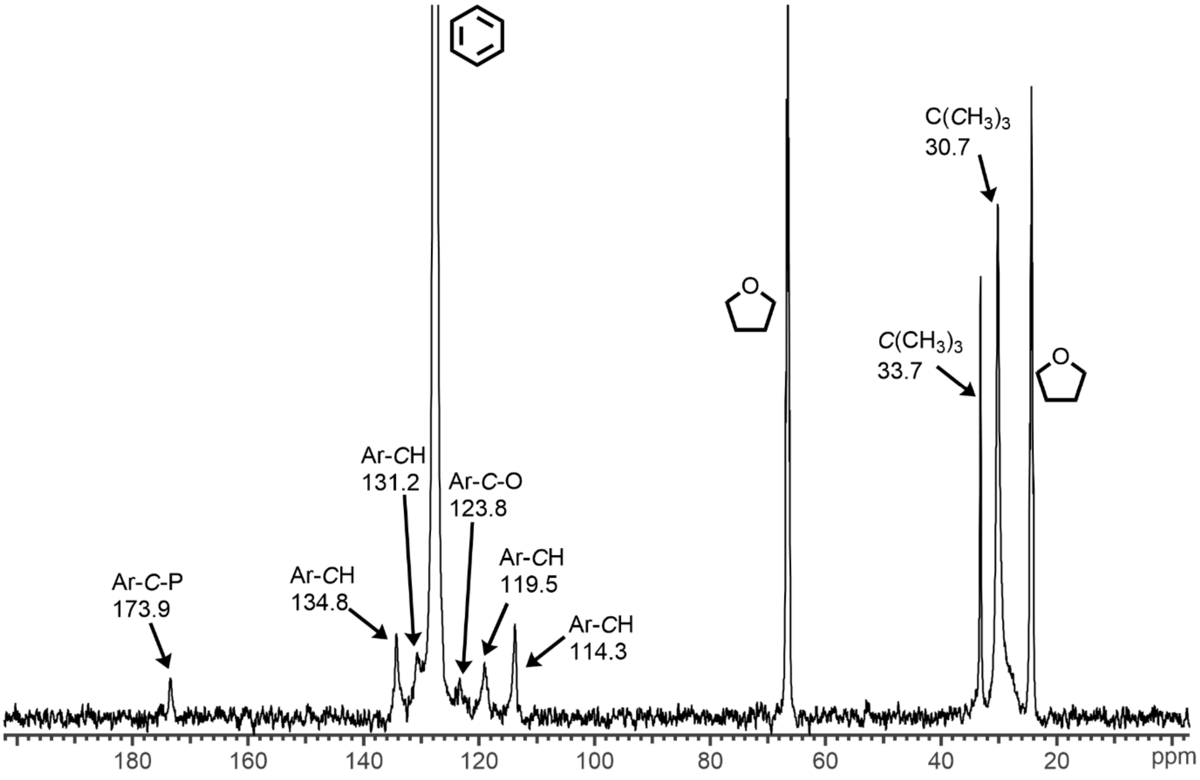

Figure S15 ^{13}C{H} NMR Spectrum of **3-La** (C_6D_6/C_4D_8O, 298 K, 125.78 MHz).

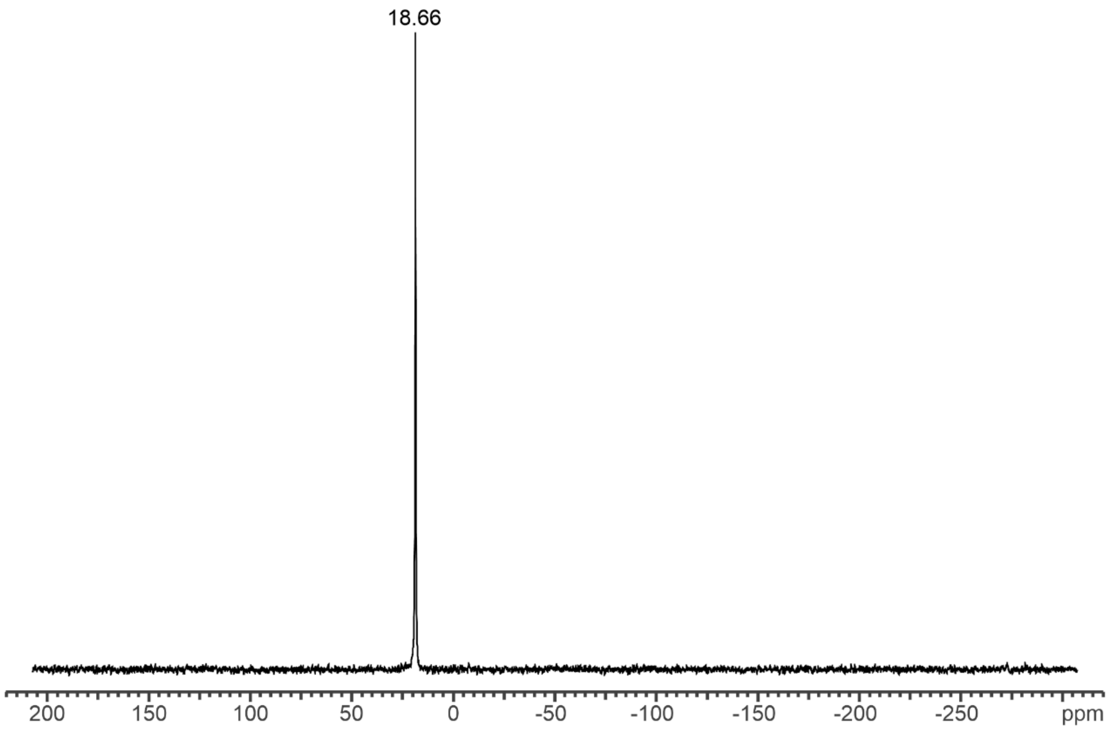

Figure S16: $^{31}P\{^1H\}$ NMR spectrum of **3-La** (C_6D_6, 298 K, 162 MHz).

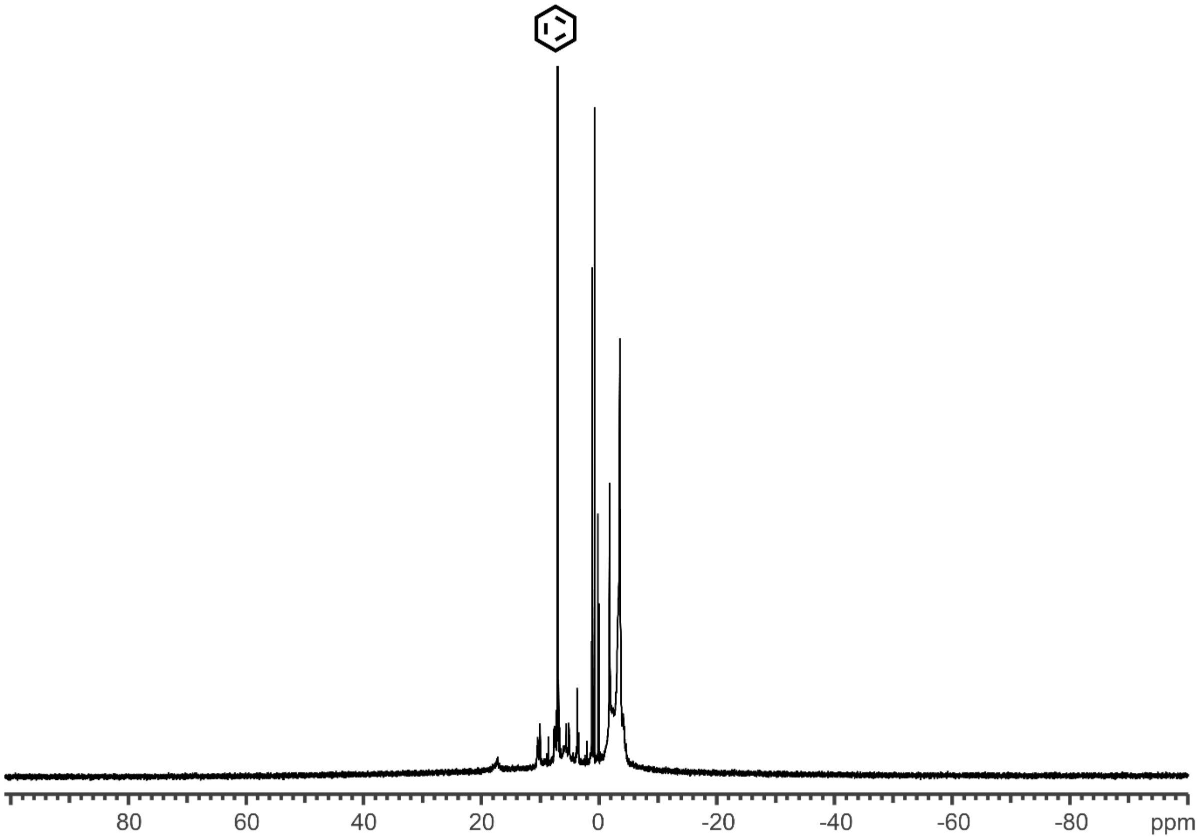

Figure S17A: ^1H NMR Spectrum of **3-Ce** (C_6D_6, 298 K, 400 MHz).

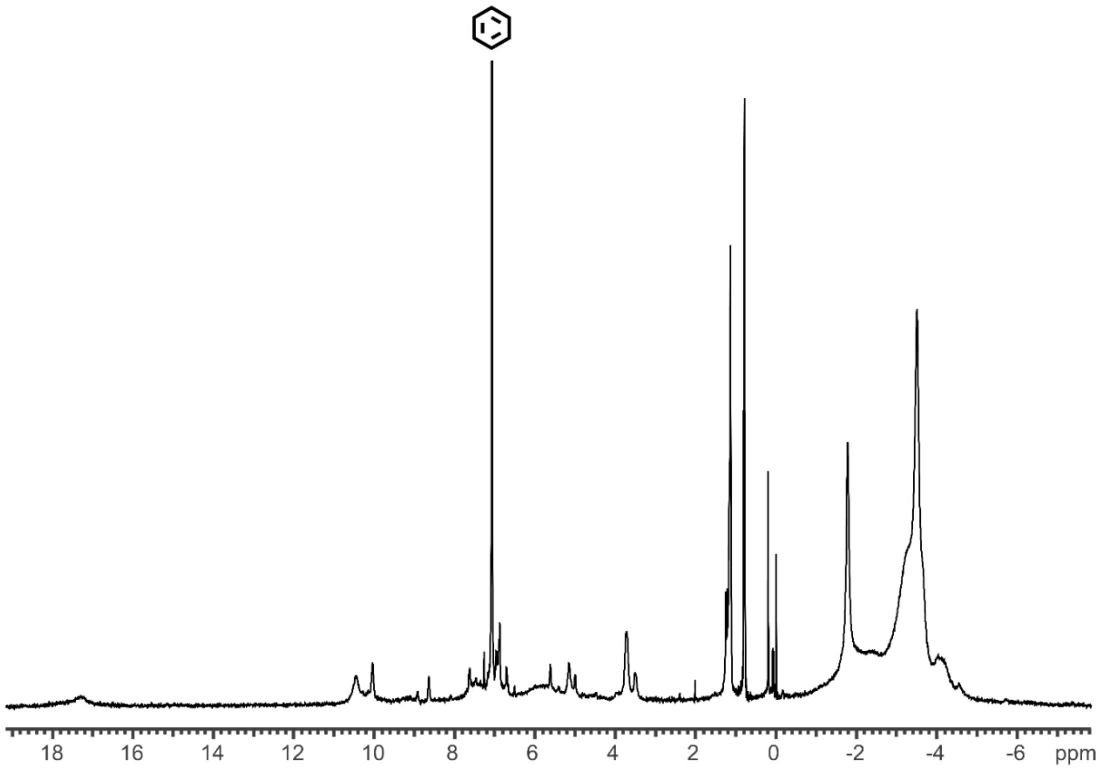

Figure S17B: ^{1}H NMR Spectrum of **3-Ce** (C_6D_6, 298 K, 400 MHz), showing region between -7 and 18 ppm.

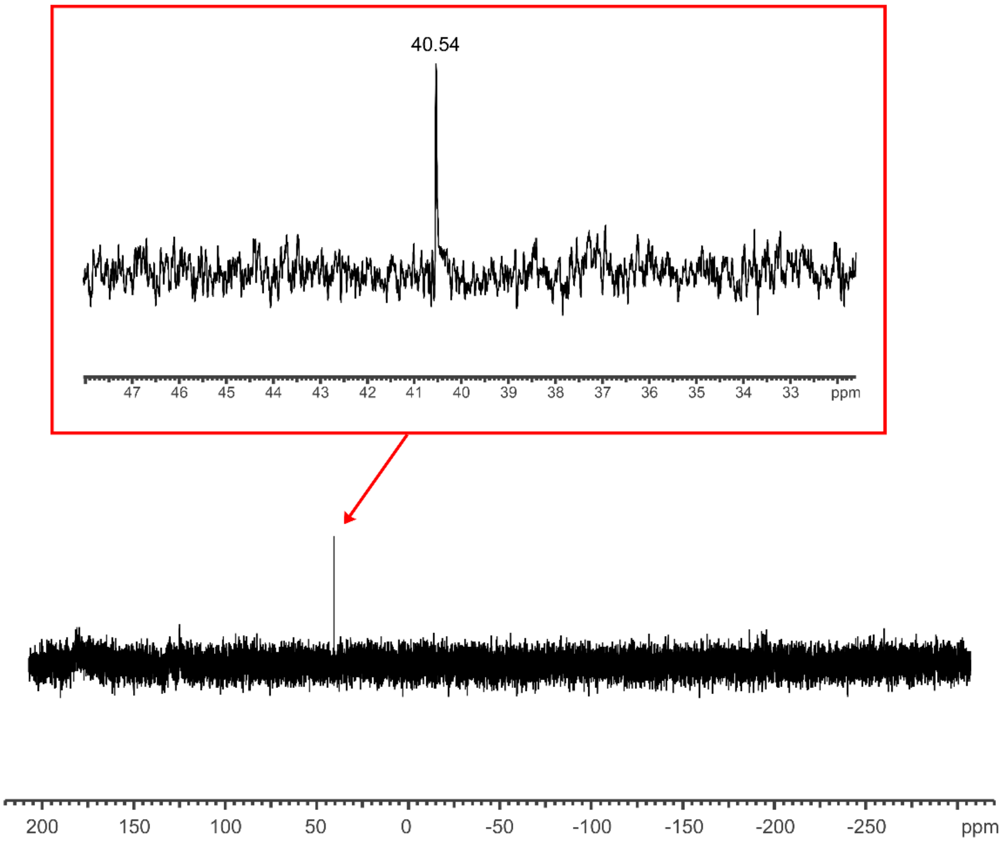

Figure S18: $^{31}P\{^{1}H\}$ NMR spectrum of **3-Ce** (C_6D_6, 298 K, 162 MHz). Inset shows region around signal at 40.54 ppm.

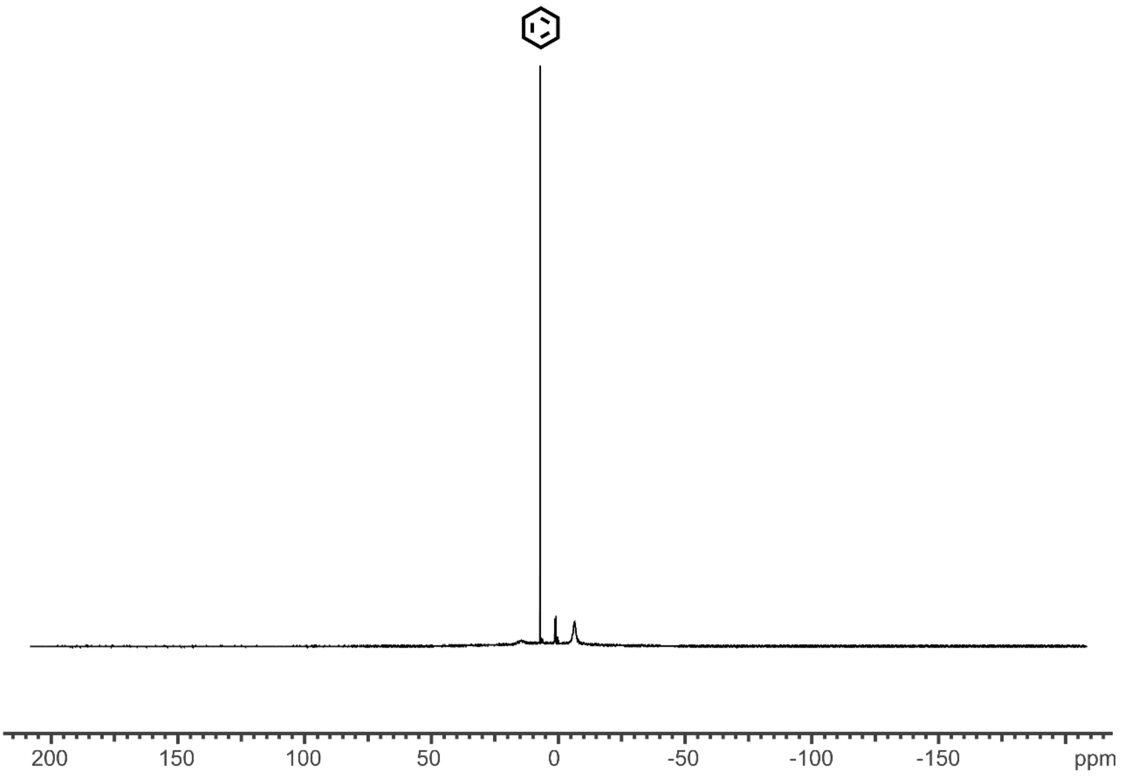

Figure S19A: ^{1}H NMR Spectrum of **3-Pr** (C_6D_6, 298 K, 400 MHz).

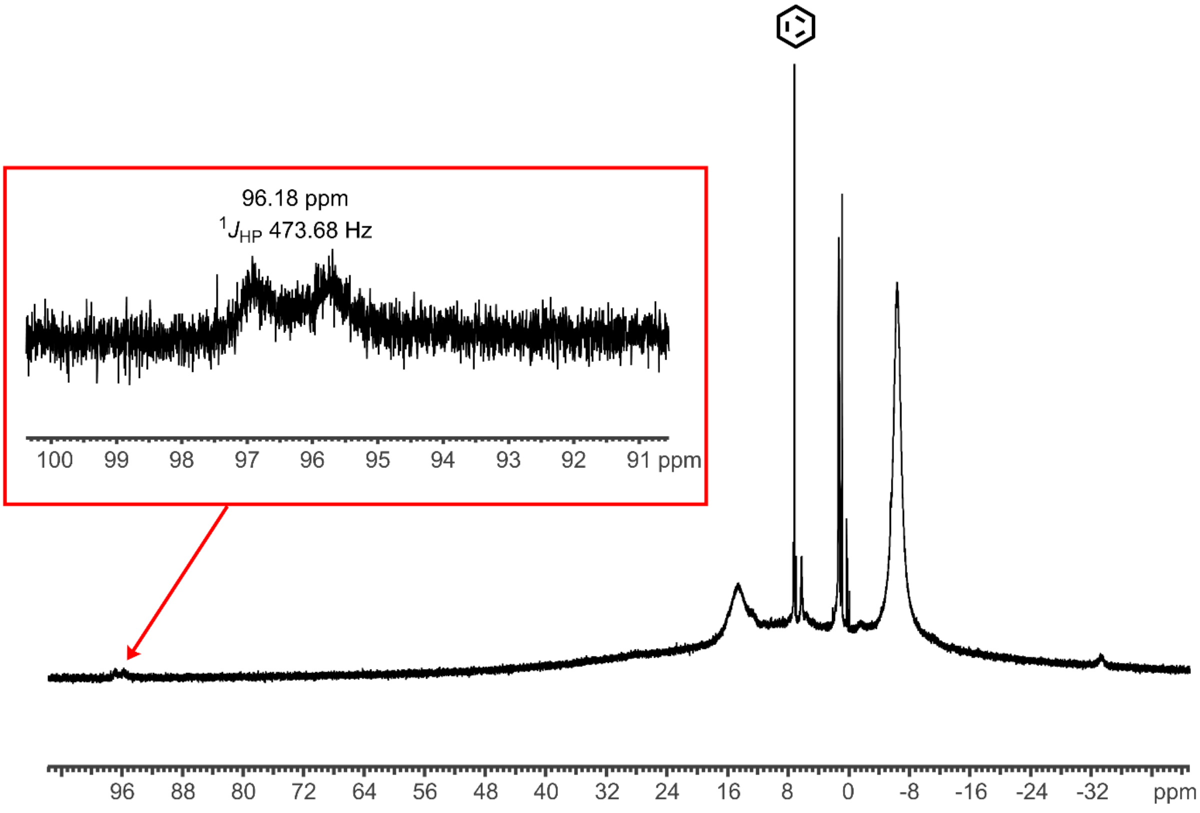

Figure S19B: ^{1}H NMR Spectrum of **3-Pr** (C_6D_6, 298 K, 400 MHz) zoomed in the region between -40 and 100 ppm. Inset shows broad doublet at 96.18 ppm.

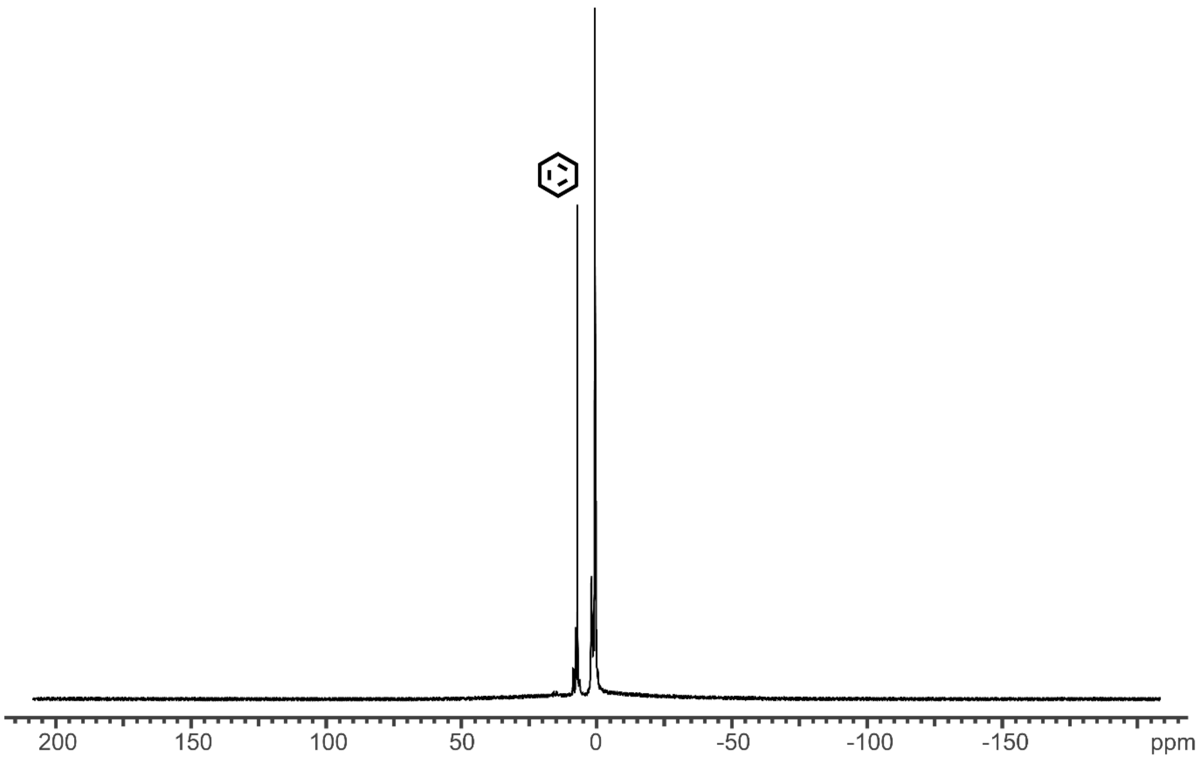

Figure S20A: ^{1}H NMR spectrum of **3-Sm** (C_6D_6, 298 K, 400 MHz).

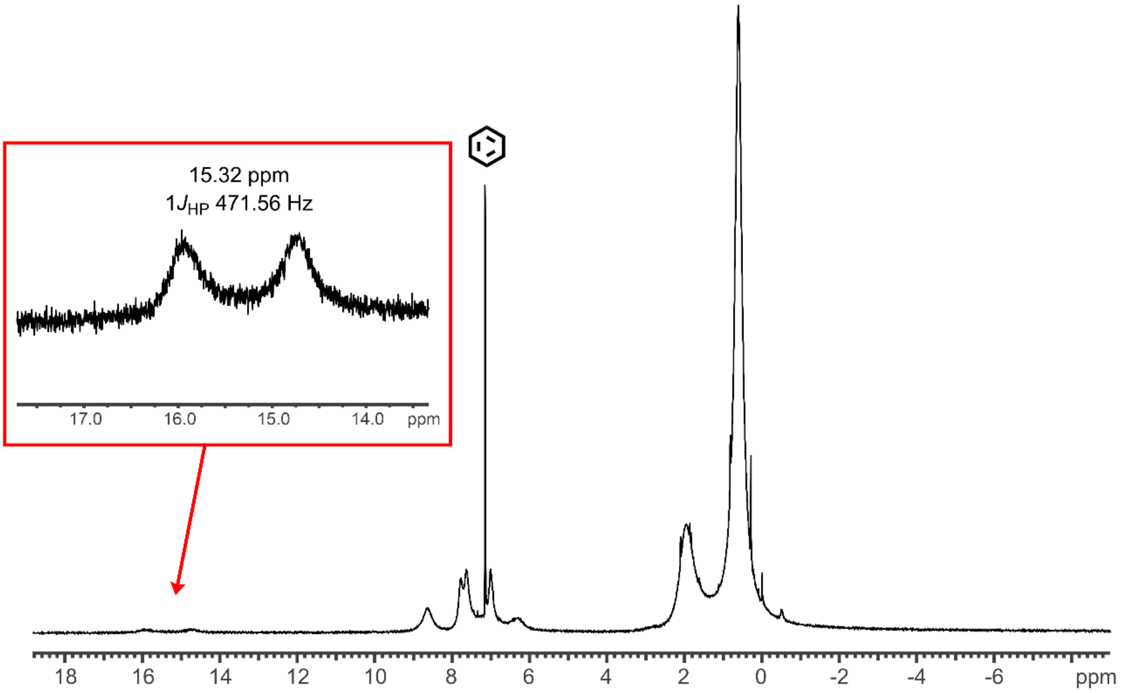

Figure S20B: ^{1}H NMR spectrum of **3-Sm** (C_6D_6, 298 K, 400 MHz) zoomed in the region between -8 and 18 ppm. Inset shows doublet at 15.32 ppm.

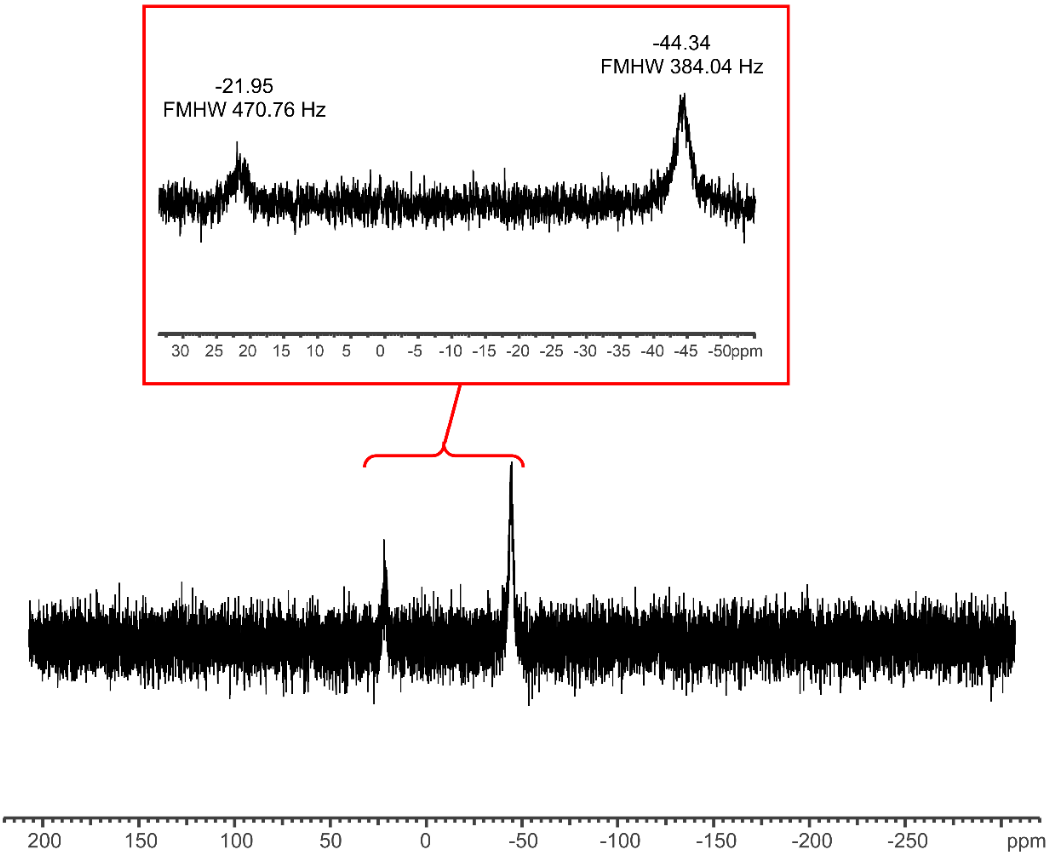

Figure S21: $^{31}P\{^{1}H\}$ NMR Spectrum of **3-Sm** (C_6D_6, 298 K, 162 MHz). Inset shows region between -50 and 30 ppm.

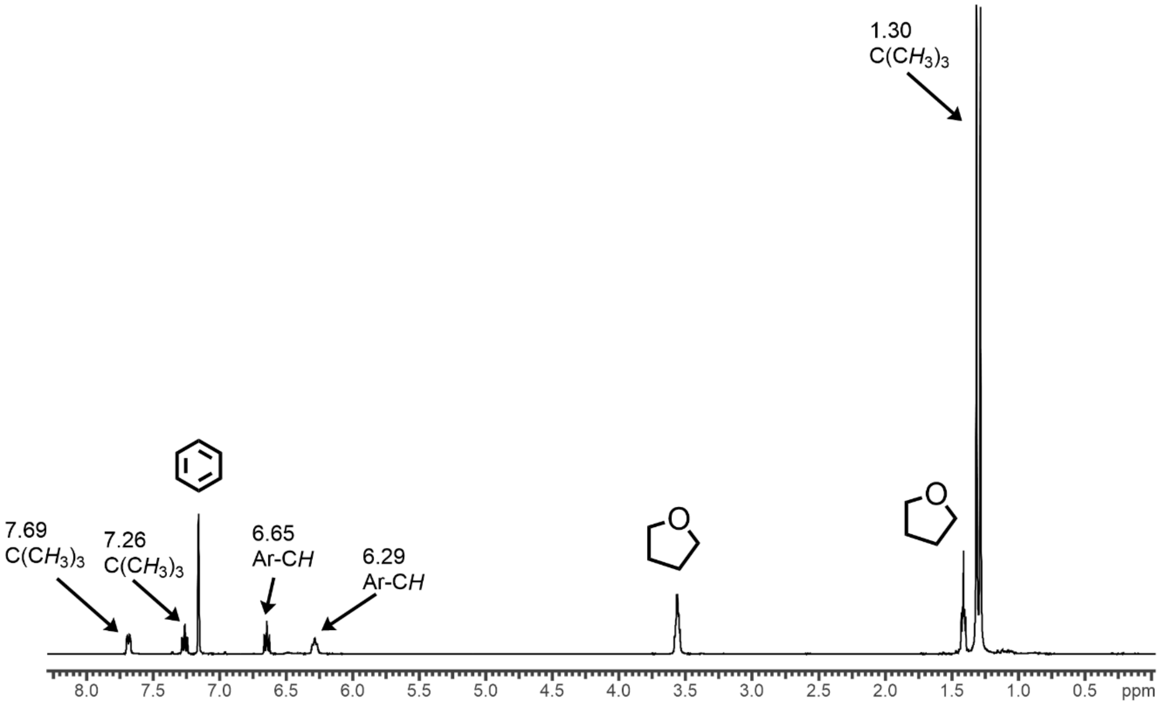

Figure S22: ^{1}H NMR spectrum of $K[^{t}Bu_2P(C_6H_4)O]$ (C_6D_6/C_4D_8O, 298 K, 400 MHz).

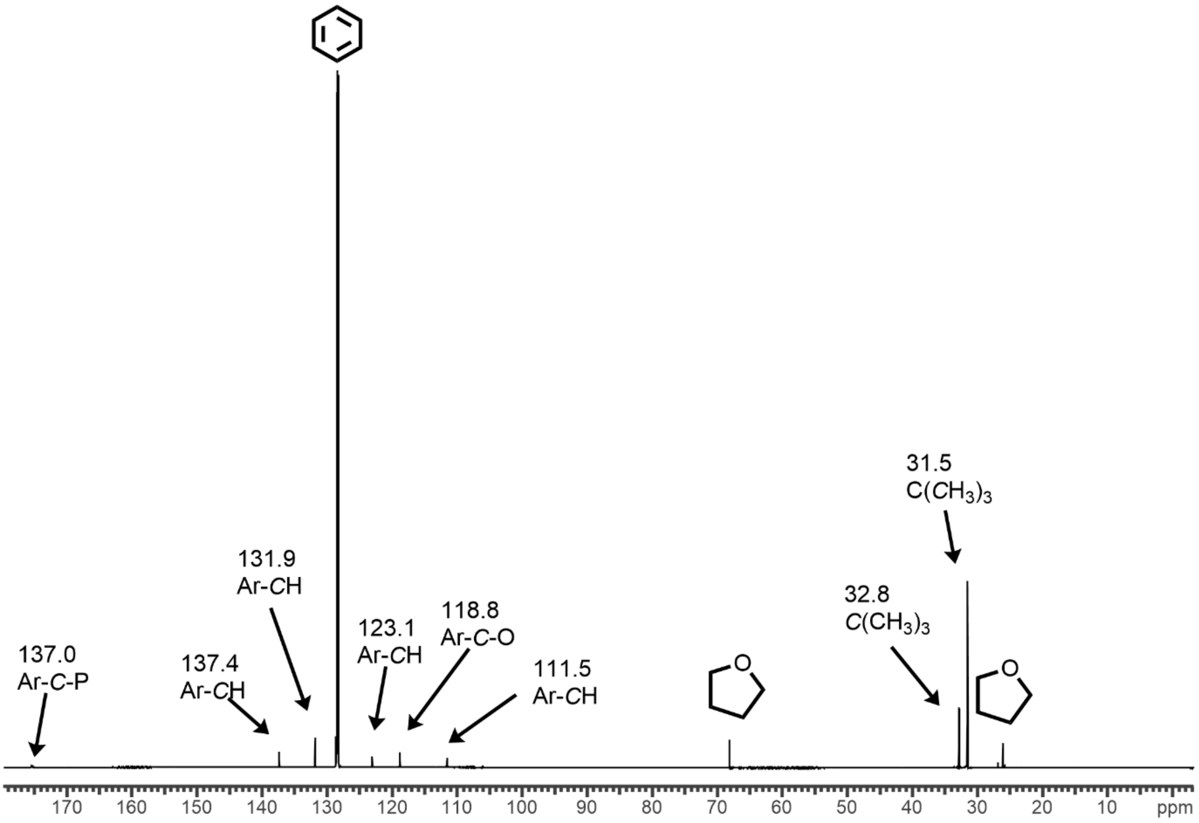

Figure S23: $^{13}C\{^1H\}$ NMR spectrum of $K[^tBu_2P(C_6H_4)O]$ (C_6D_6/C_4D_8O, 298 K, 201.21 MHz).

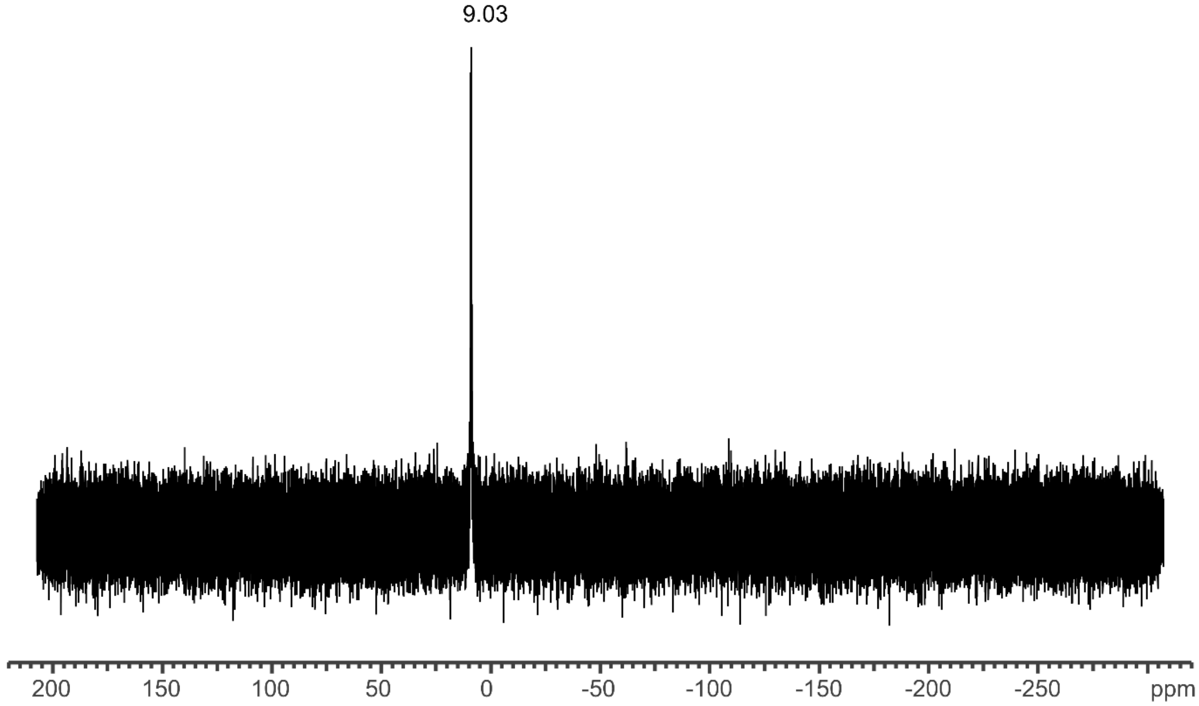

Figure S24: $^{31}P\{^1H\}$ NMR Spectrum of $K[^tBu_2P(C_6H_4)O]$ (C_6D_6, 298 K, 162 MHz).

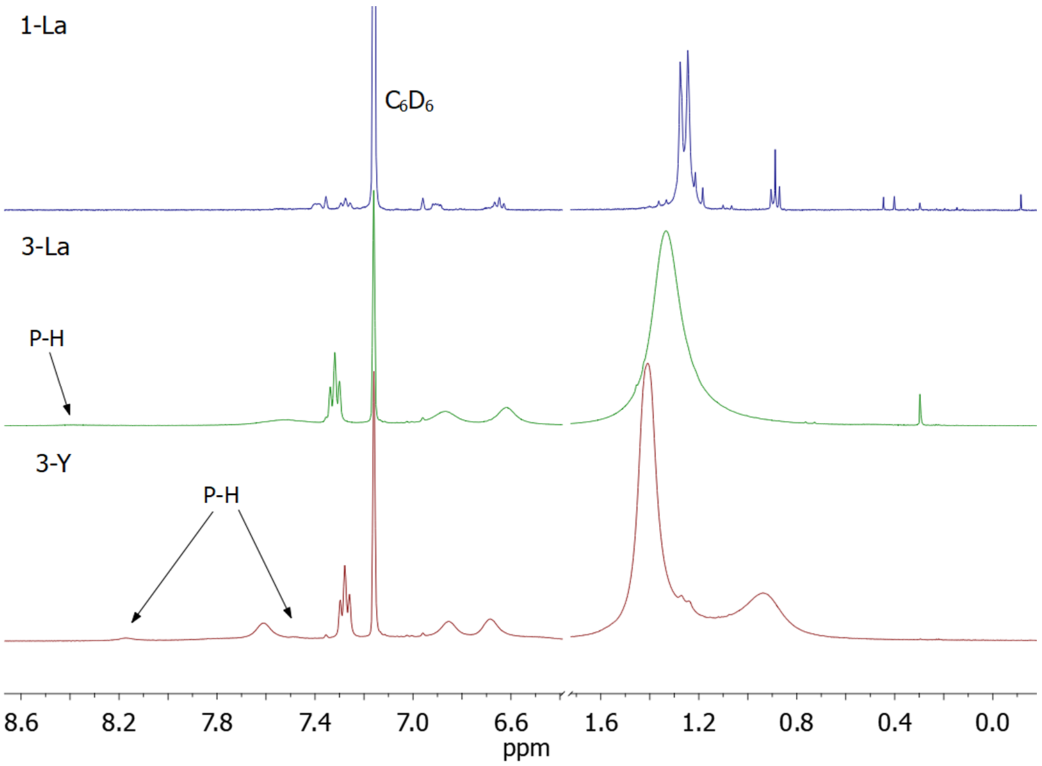

Figure S25: Stacked ^{1}H NMR spectra of **1-La**, **3-La** and **3-Y**, zoomed in the aromatic region (left) and aliphatic region (right).

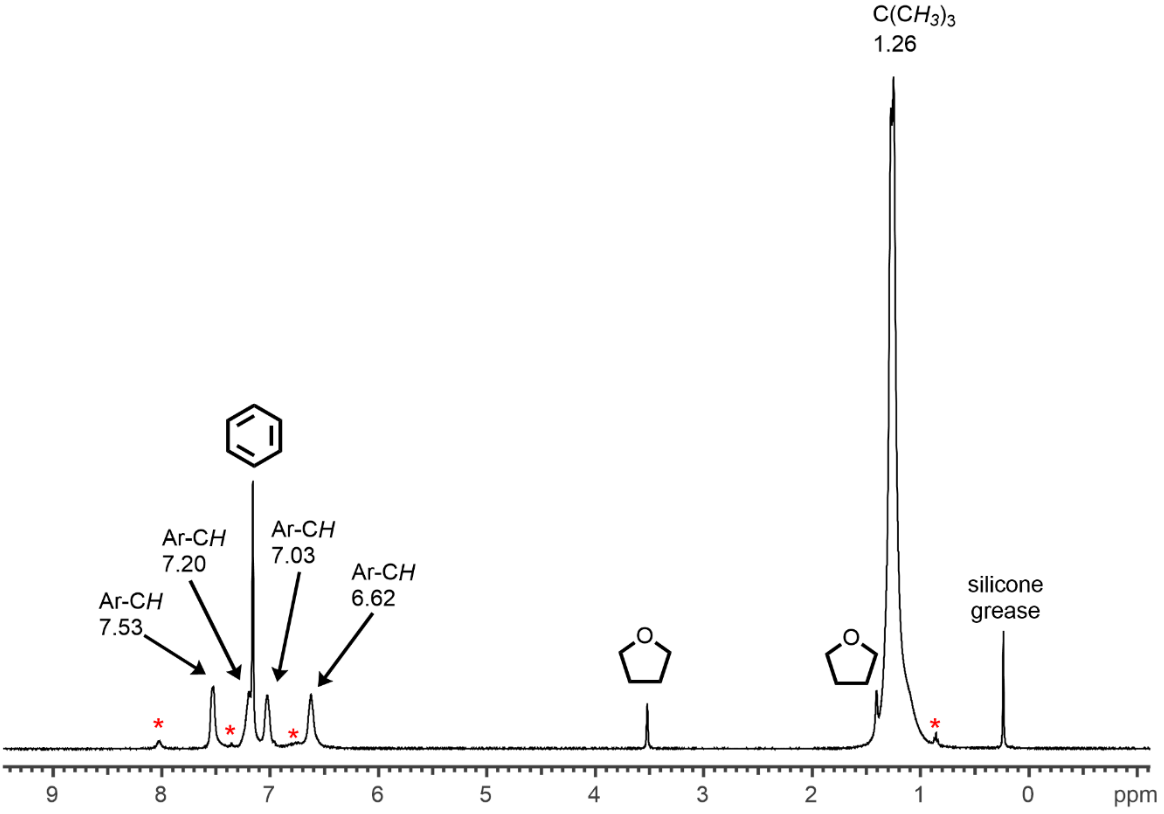

Figure S26: ^{1}H NMR Spectrum of **4** (C_6D_6/C_4D_8O, 298 K, 400 MHz). * denotes unknown impurities.

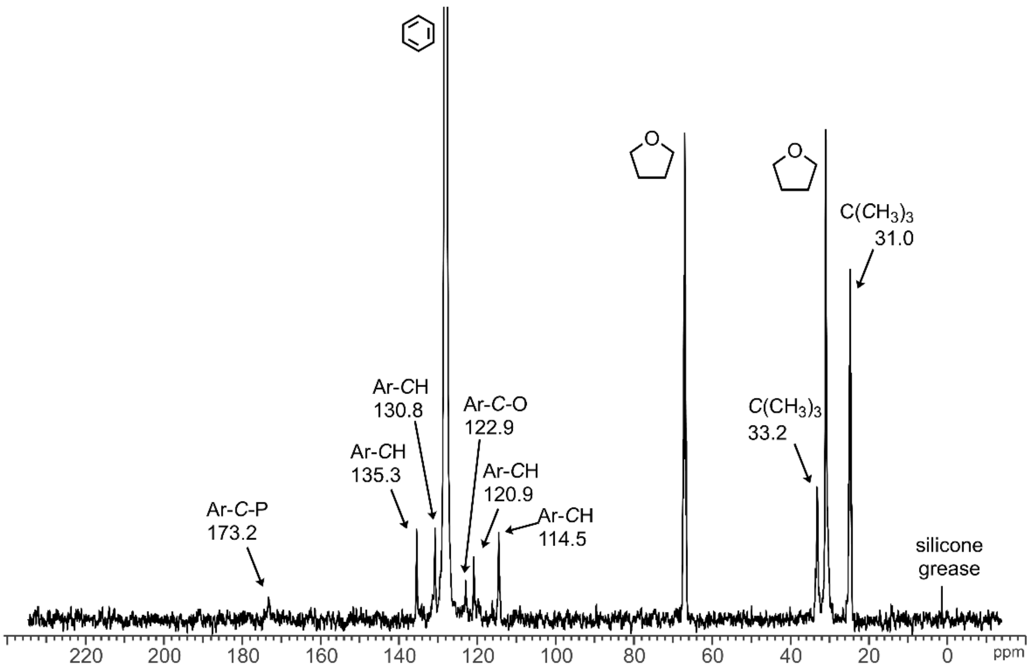

Figure S27: $^{13}C\{^{1}H\}$ NMR Spectrum of **4** (C_6D_6/C_4D_8O, 298 K, 100 MHz).

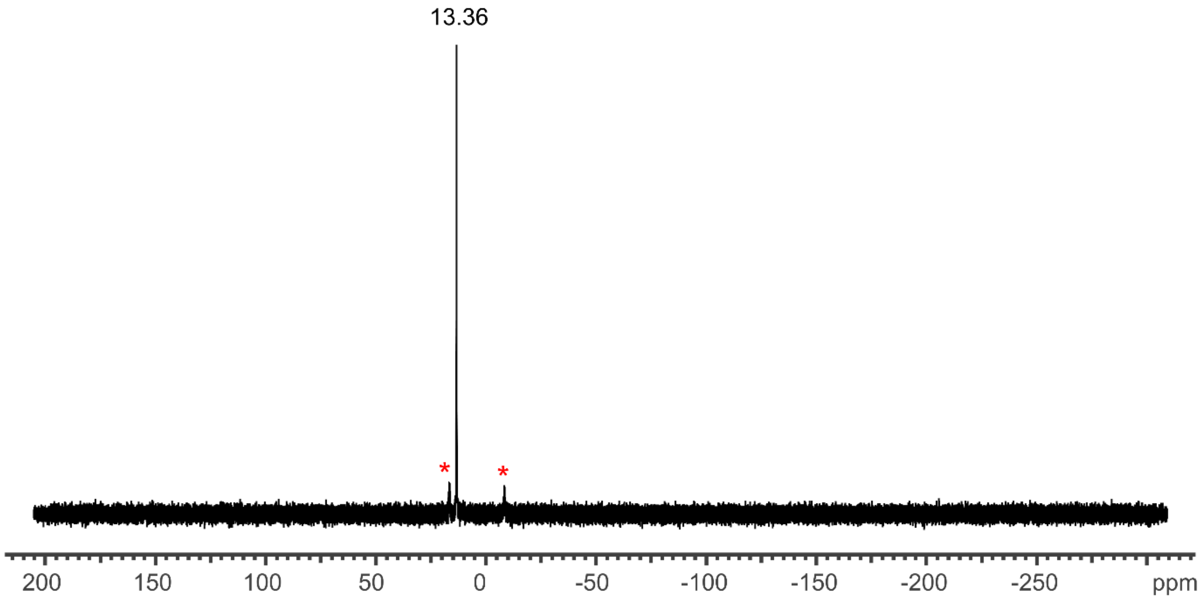

Figure S28: $^{31}P\{^{1}H\}$ NMR Spectrum of **4** (C_6D_6/C_4D_8O, 298 K, 162 MHz). * denotes unknown impurities.

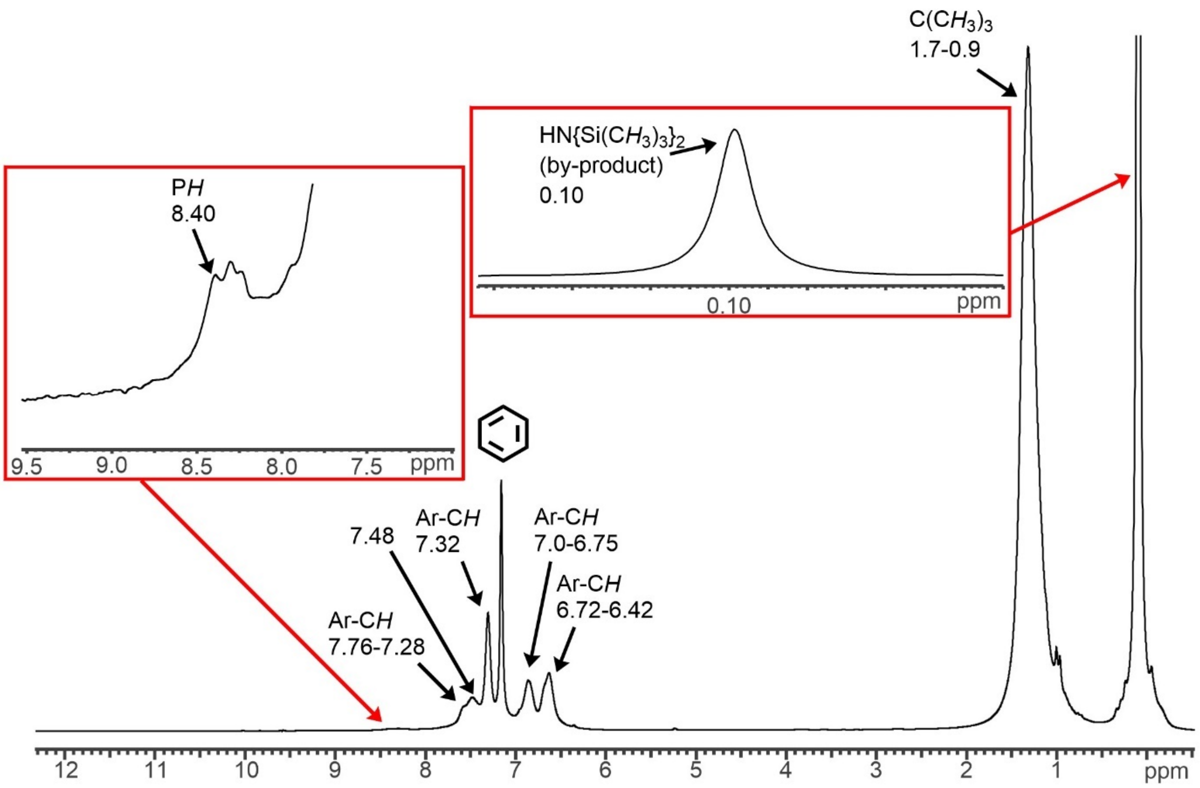

Figure S29: ^{1}H NMR spectrum of the reaction of [La{N(SiMe$_3$)$_2$}$_3$] with 4 equivalents of 2-di-tertbutylphosphinophenol (C_6D_6, 298 K, 400 MHz), showing **3-La** as the major product. Insets show the peak at 8.40 ppm and the full peak at 0.10 ppm.

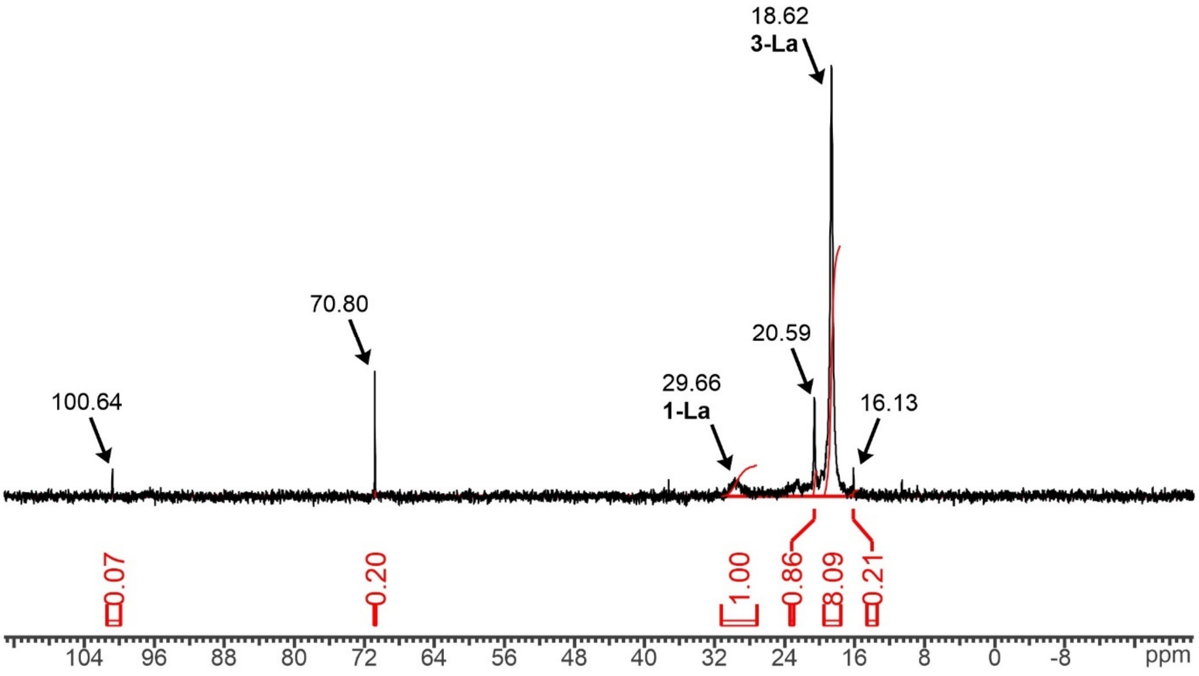

Figure S30: ^{31}P NMR spectrum of the reaction between [La{N(SiMe$_3$)$_2$}$_3$] with 4 equivalents of 2-di-tertbutylphosphinophenol (C_6D_6, 298 K, 162 MHz), showing **3-La** as the major product.

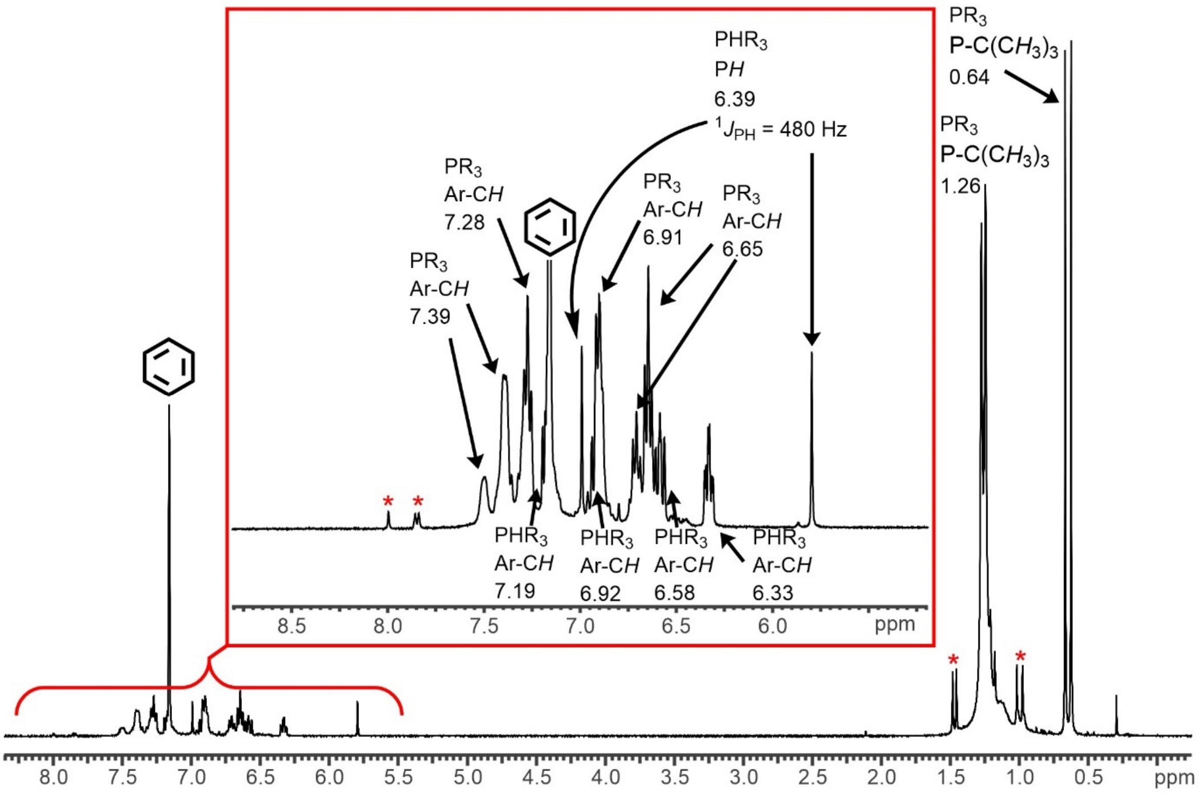

Figure S31: ^{1}H NMR spectrum of the reaction of **3-La** with 1 equivalent of $B(C_6F_5)_3$, showing the formation of **1-La** (labelled PR_3) and the phosphonium-borate zwitterionic adduct (labelled PHR_3) (C_6D_6, 298K, 400 MHz). Inset shows aromatic region (5.5 – 8.5 ppm). * denotes unknown impurities.

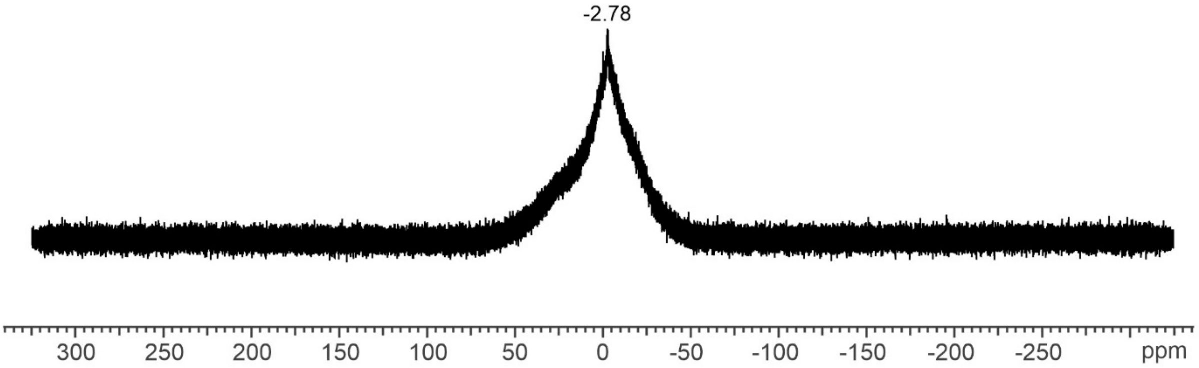

Figure S32: $^{11}B\{H\}$ NMR spectrum of the reaction of **3-La** with 1 equivalent of $B(C_6F_5)_3$ (C_6D_6, 298K, 128 MHz).

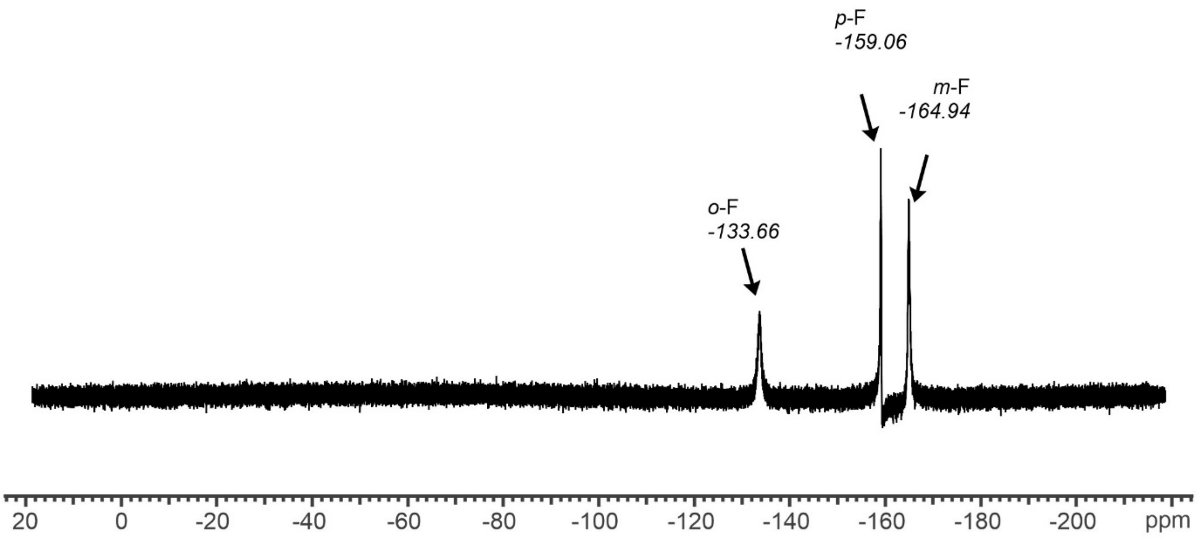

Figure S33: ^{19}F NMR spectrum of the reaction of **3-La** with 1 equivalent of $B(C_6F_5)_3$ (C_6D_6, 298 K, 376 MHz).

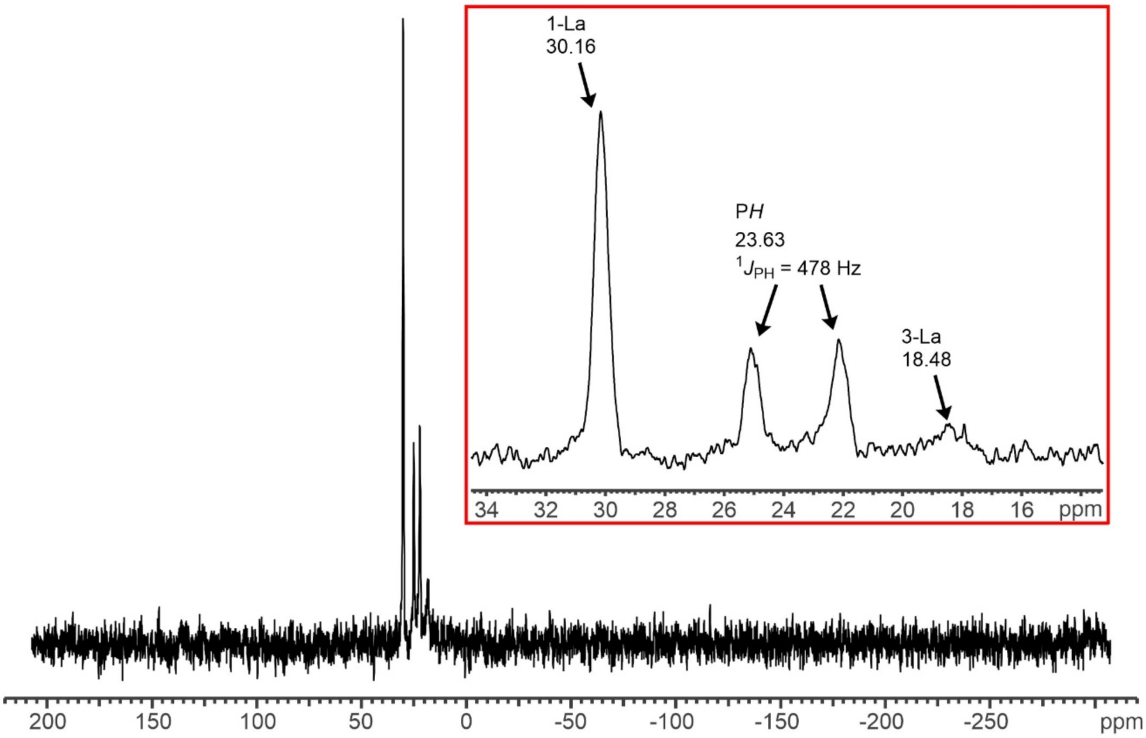

Figure S34: ^{31}P NMR spectrum of the reaction of **3-La** with 1 equivalent of $B(C_6F_5)_3$ (C_6D_6, 298 K, 162 MHz), showing a singlet at 30.16 ppm corresponding to **1-La**, a doublet corresponding to the phosphonium-borate zwitterionic adduct, and a weak, broad singlet corresponding to unreacted **3-La**.

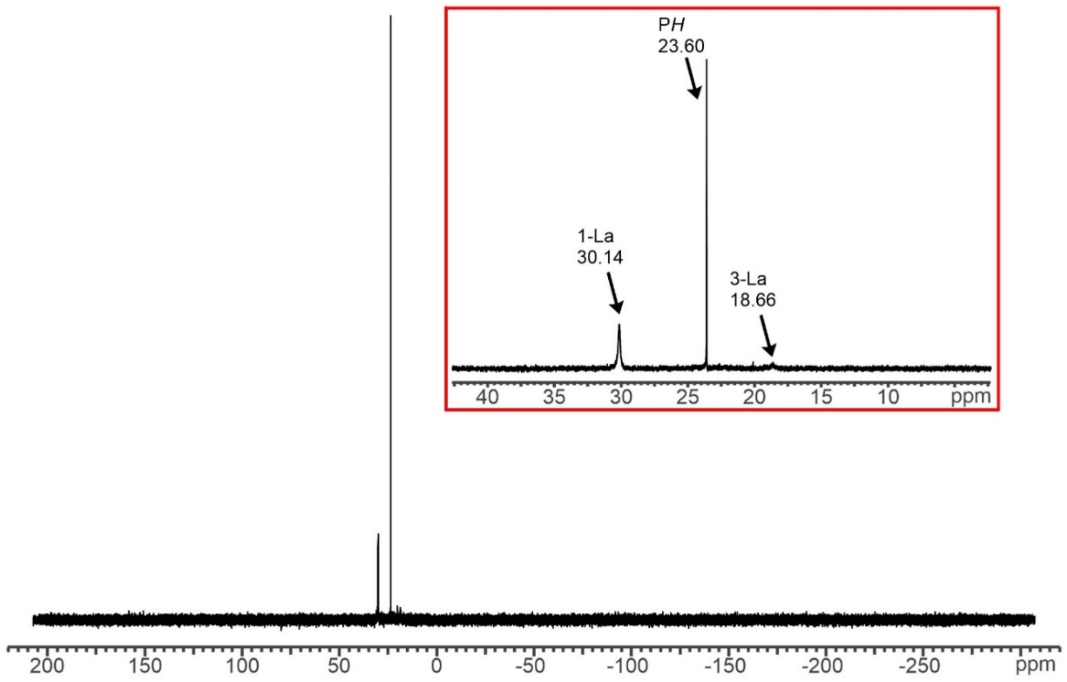

Figure S35: $^{31}P\{H\}$ NMR spectrum of the reaction of **3-La** with 1 equivalent of $B(C_6F_5)_3$ (C_6D_6, 298 K, 162 MHz), showing a singlet at 30.16 ppm corresponding to **1-La**, a singlet corresponding to the phosphonium-borate zwitterionic adduct, and a weak, broad singlet corresponding to unreacted **3-La**.

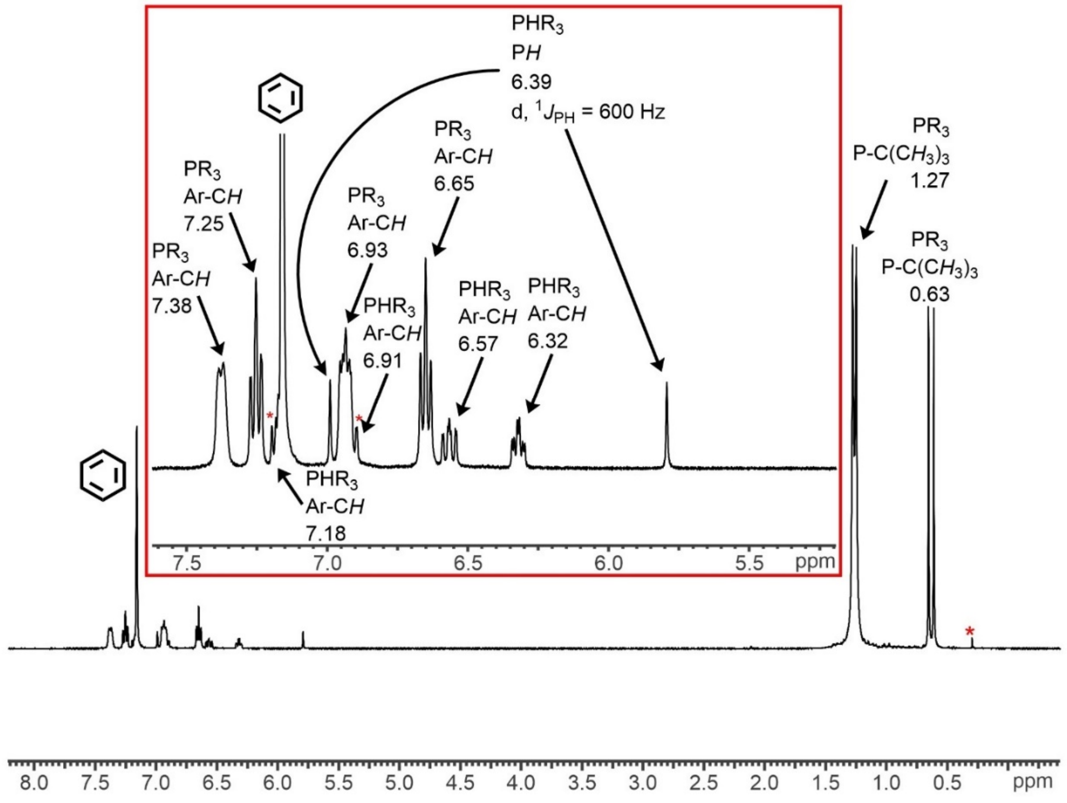

Figure S36: ^{1}H NMR spectrum of the reaction of **3-Y** with 1 equivalent of $B(C_6F_5)_3$ (C_6D_6, 298 K, 400 MHz), showing the formation of **1-Y** (labelled PR_3) and the phosphonium-borate zwitterionic adduct (labelled PHR_3). Inset shows aromatic region (5.5 – 7.5 ppm).

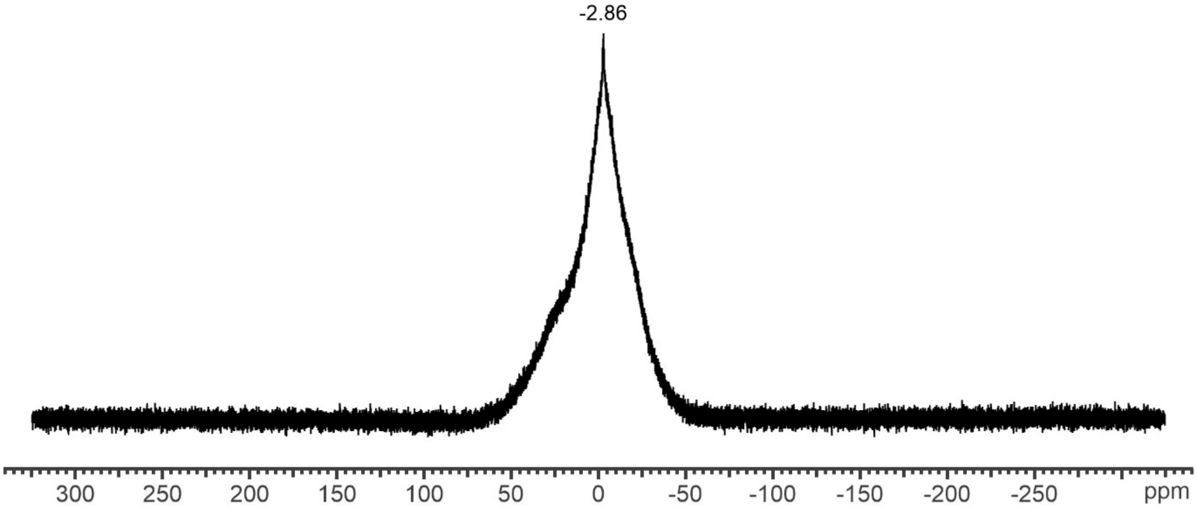

Figure S37: $^{11}B\{H\}$ NMR spectrum of the reaction of **3-Y** with 1 equivalent of $B(C_6F_5)_3$ (C_6D_6, 298 K, 128 MHz), showing a single peak at –2.86 ppm.

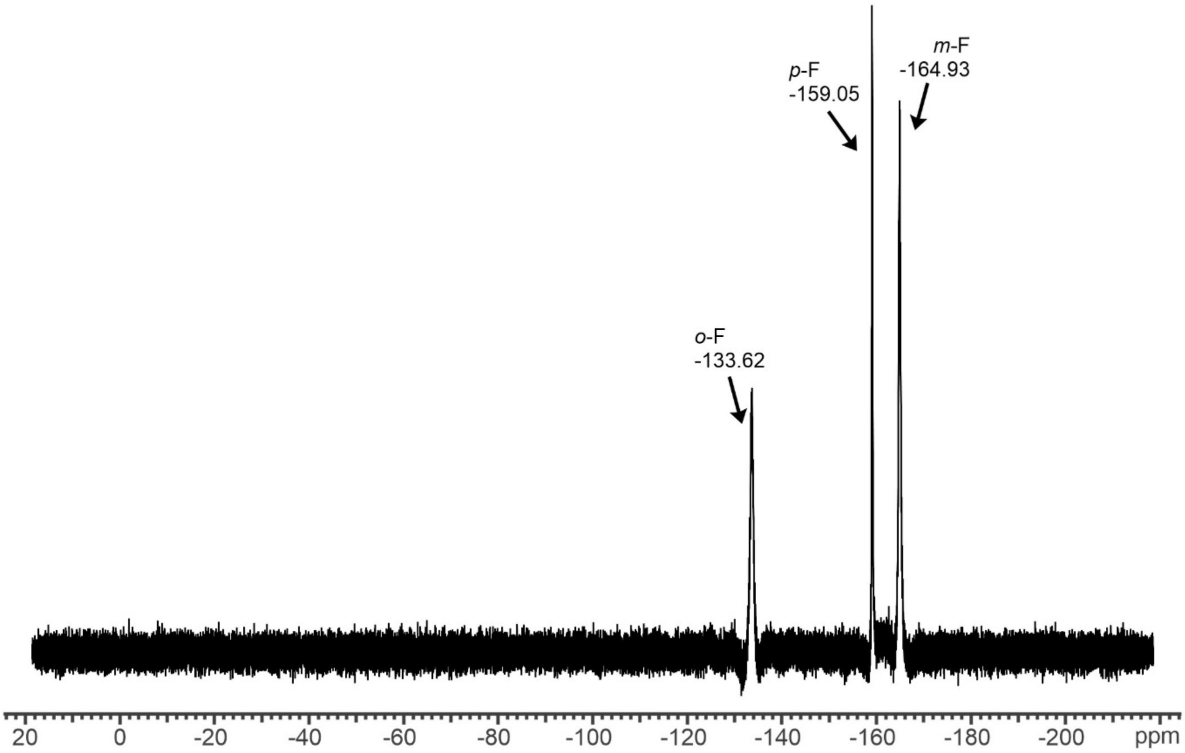

Figure S38: $^{19}F\{H\}$ NMR spectrum of the reaction of **3-Y** with 1 equivalent of $B(C_6F_5)_3$ (C_6D_6, 298 K, 376 MHz).

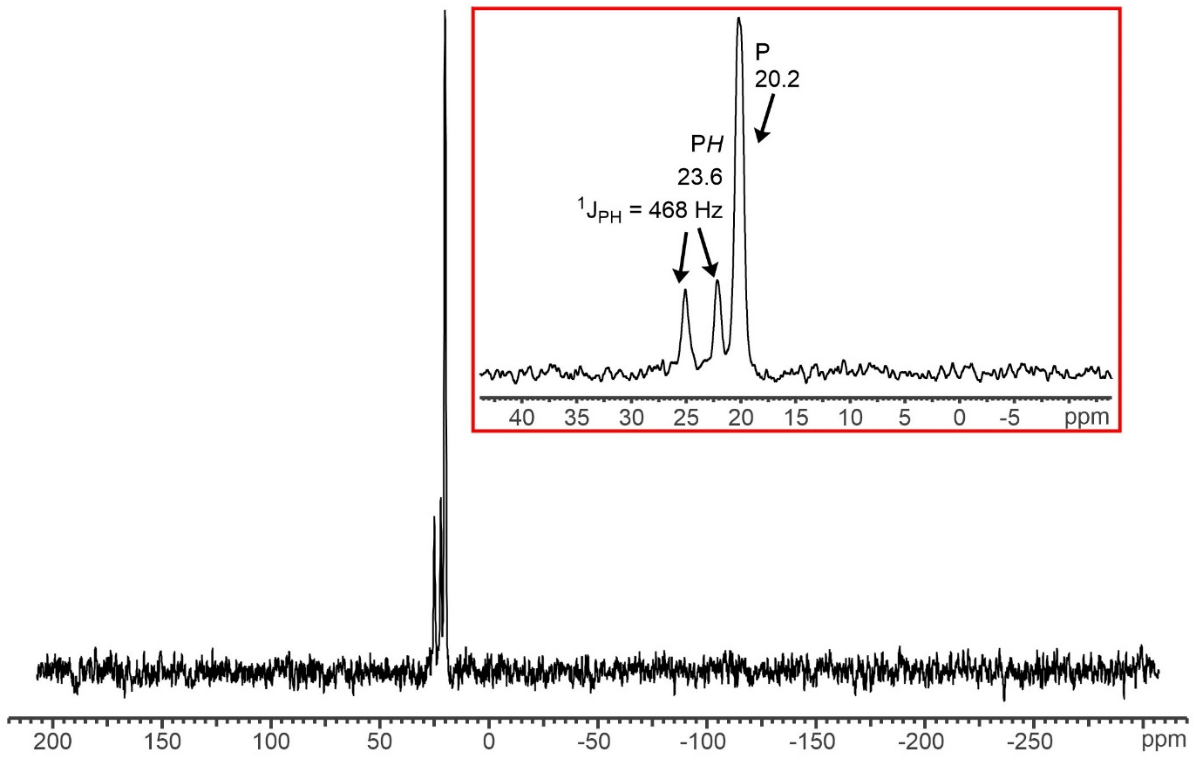

Figure S39: ^{31}P NMR spectrum of the reaction of **3-Y** with 1 equivalent of $B(C_6F_5)_3$, showing a singlet at 20.2 ppm, likely corresponding to **1-Y**, and a doublet corresponding to the phosphonium-borate zwitterionic adduct (C_6D_6, 298 K, 162 MHz). Inset shows region between -10 ppm and 40 ppm.

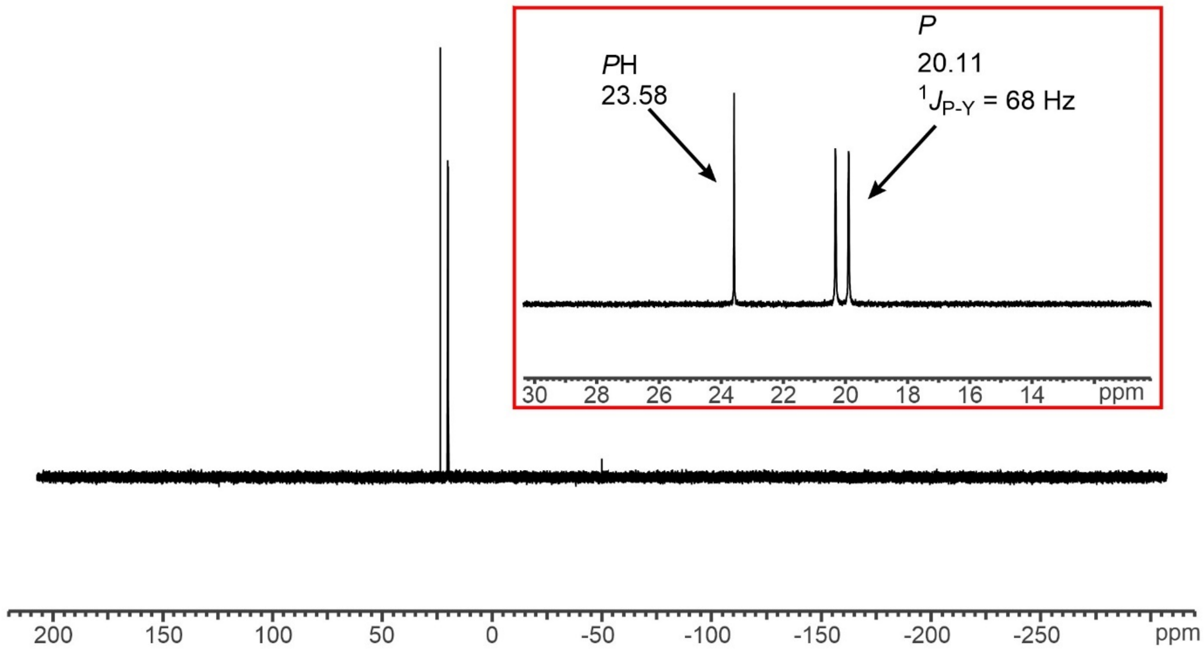

Figure S40: $^{31}P\{H\}$ NMR spectrum of the reaction of **3-Y** with 1 equivalent of $B(C_6F_5)_3$, showing a doublet at 20.2 ppm, likely corresponding to **1-Y**, and a doublet corresponding to the phosphonium-borate zwitterionic adduct (C_6D_6, 298 K, 162 MHz). Inset shows peaks between 12 ppm and 30 ppm.

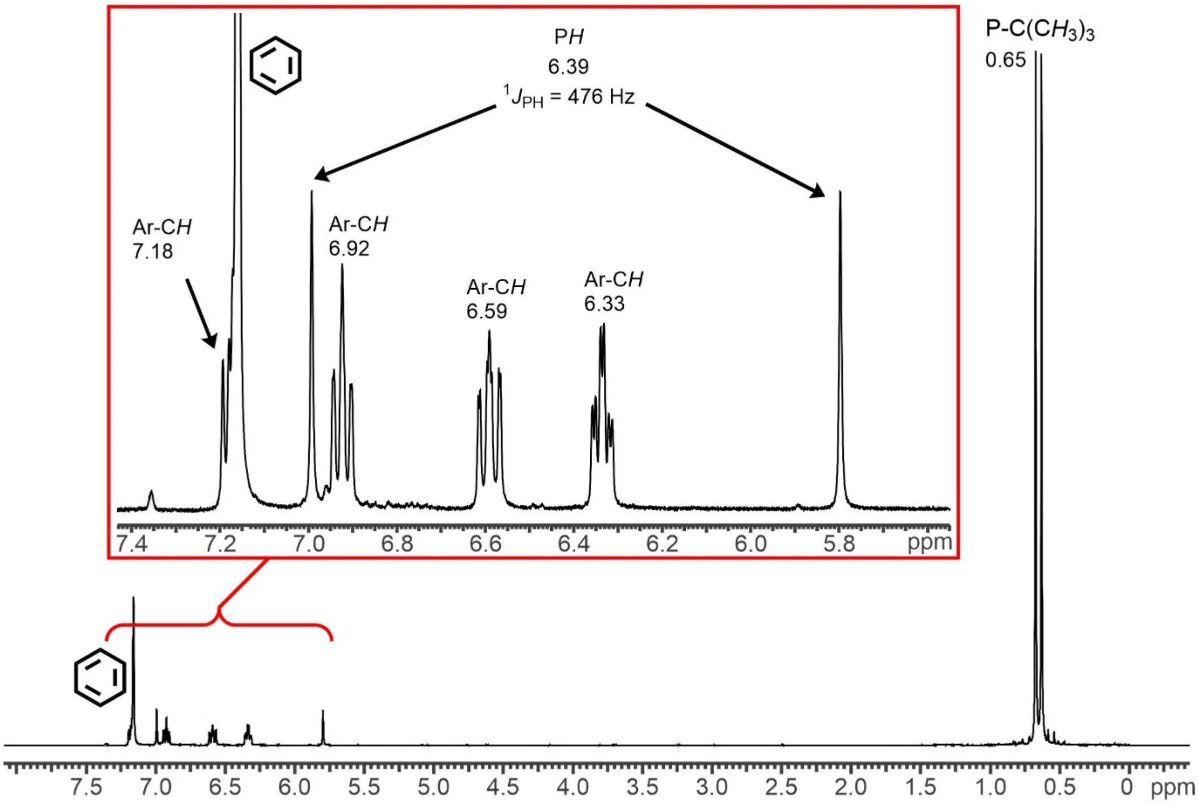

Figure S41: ^{1}H NMR spectrum of the reaction of 2-di-tert-butylphosphinophenol with 1 equivalent of $B(C_6F_5)_3$ (C_6D_6, 298 K, 400 MHz), showing the formation of the zwitterionic phosphino-borate adduct. Inset shows aromatic region (5.6 – 7.4 ppm).

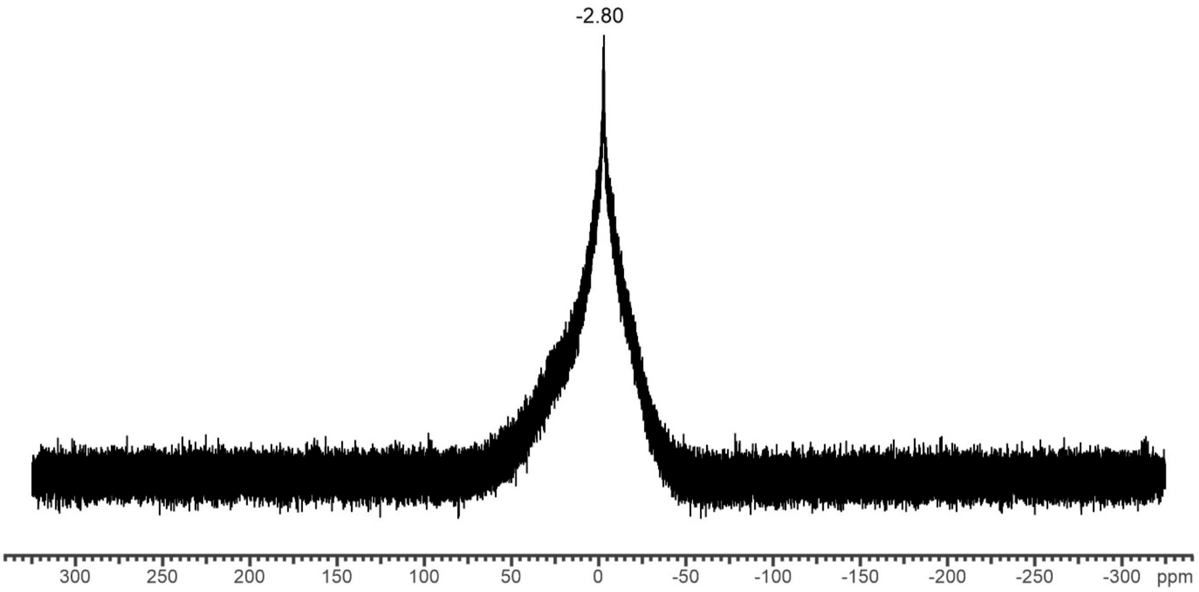

Figure S42: ^{11}B{H} NMR spectrum of the reaction of 2-di-tert-butylphosphinophenol with 1 equivalent of $B(C_6F_5)_3$ (C_6D_6, 298K, 128 MHz).

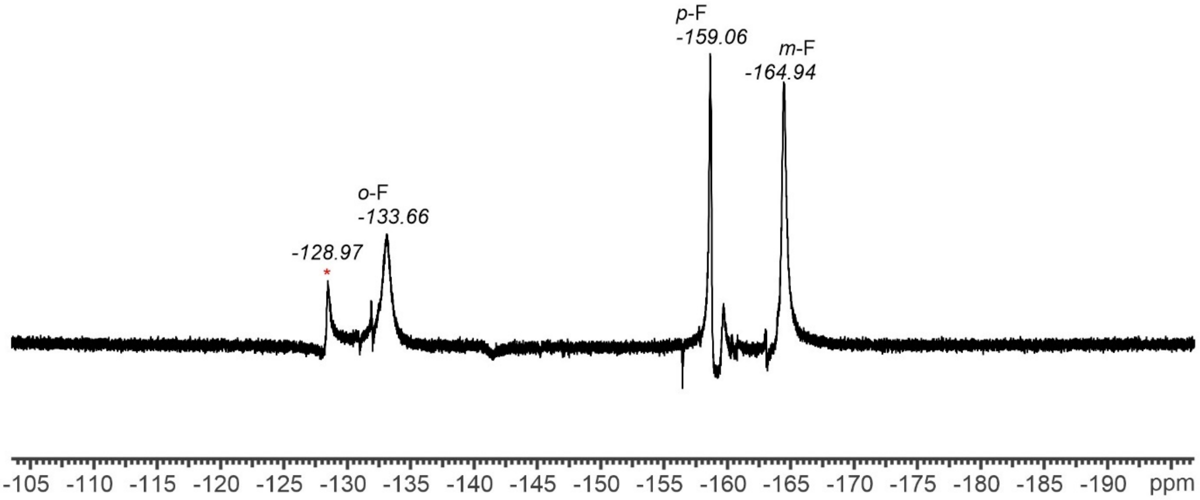

Figure S43: ^{19}F NMR spectrum of the reaction of 2-di-tert-butylphosphinophenol with 1 equivalent of $B(C_6F_5)_3$ (C_6D_6, 298 K, 376 MHz). * denotes unknown impurity.

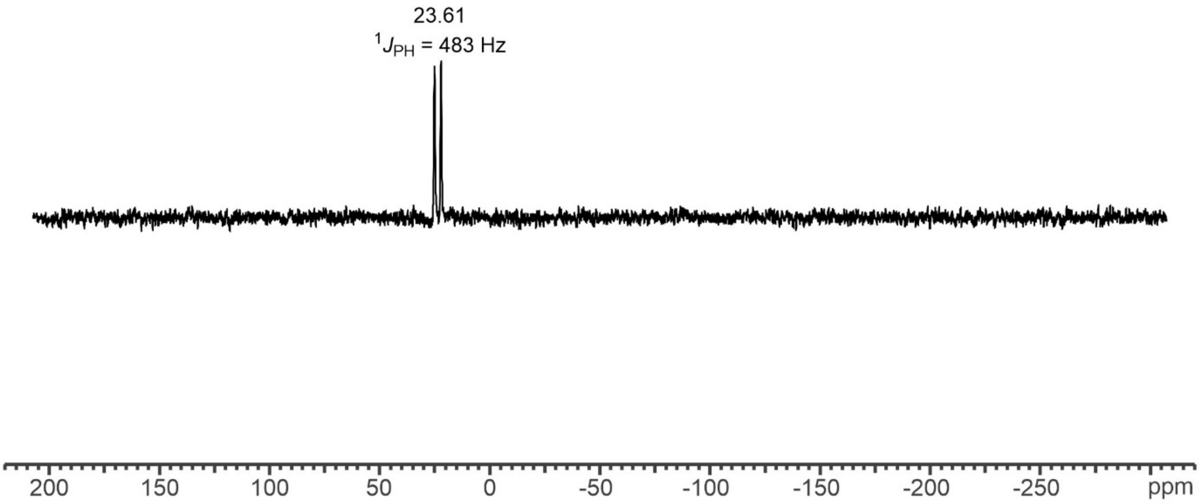

Figure S44: ^{31}P NMR spectrum of the reaction of 2-di-tert-butylphosphinophenol with 1 equivalent of $B(C_6F_5)_3$ (C_6D_6, 298 K, 162 MHz).

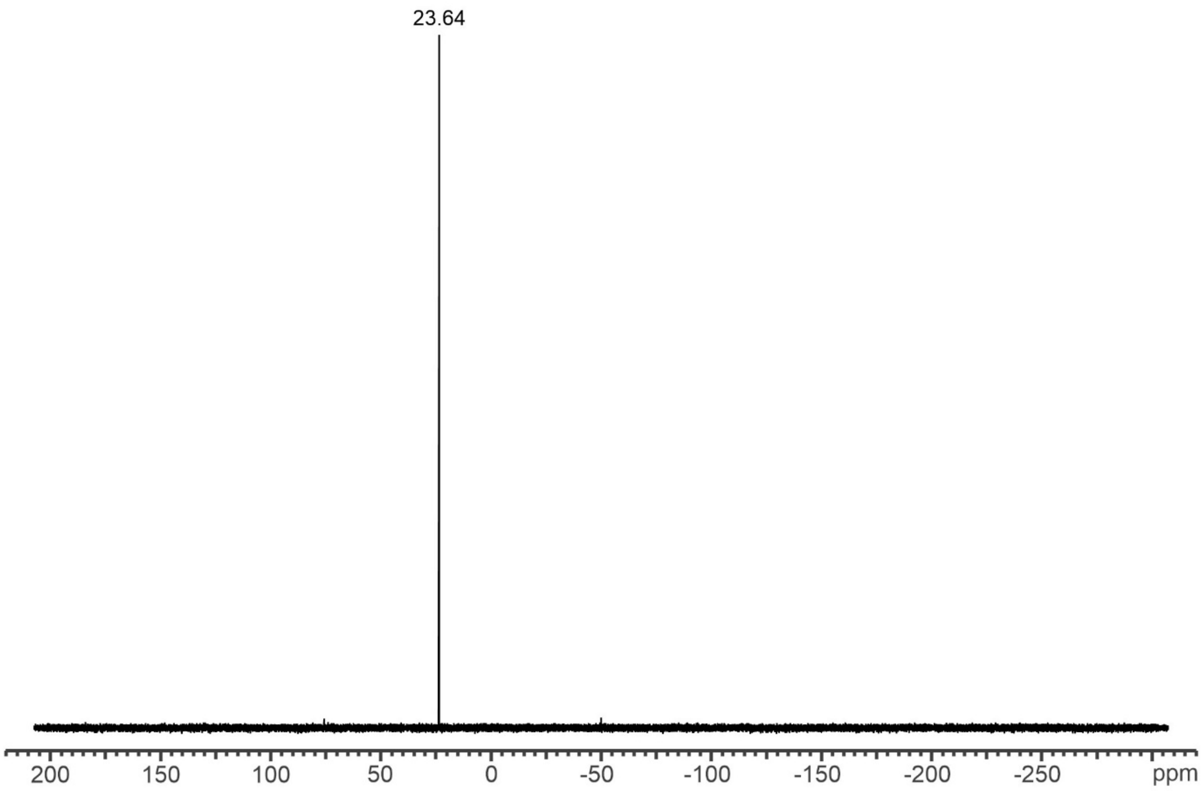

Figure S45: $^{31}P\{H\}$ NMR spectrum of the reaction of 2-di-tert-butylphosphinophenol with 1 equivalent of $B(C_6F_5)_3$ (C_6D_6, 298 K, 162 MHz).

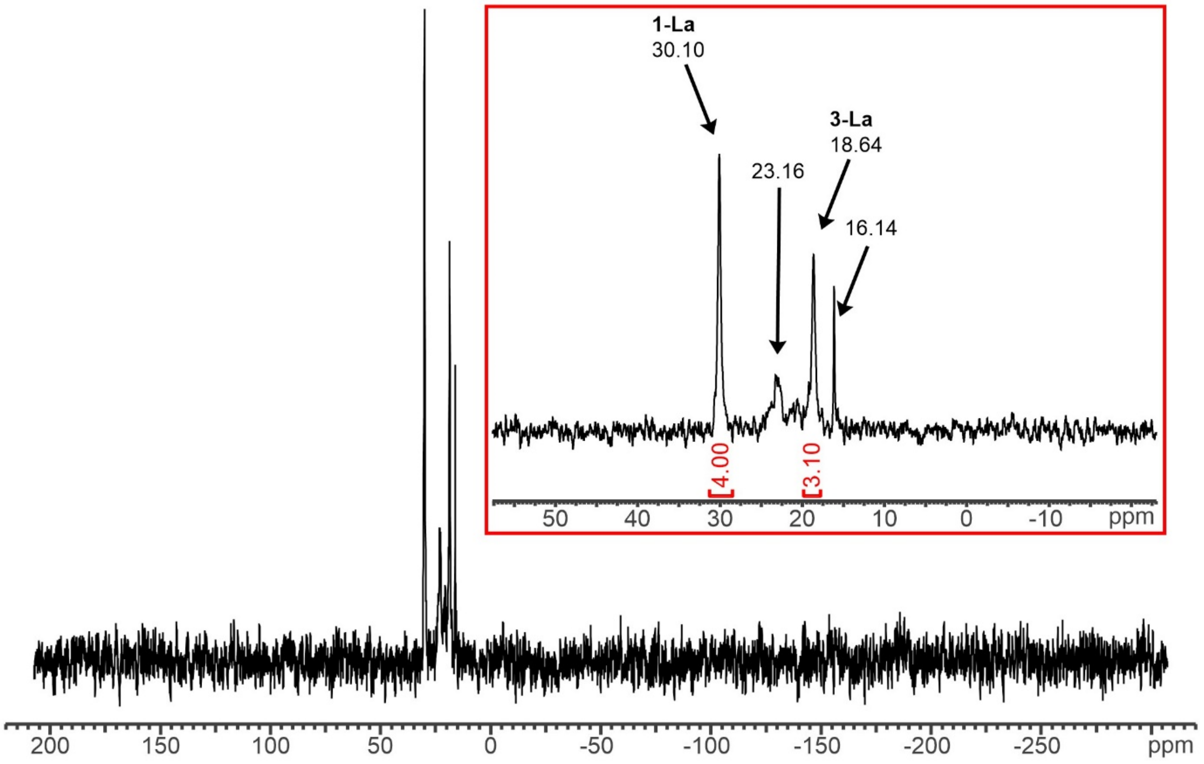

Figure S46: $^{31}P\{H\}$ NMR spectrum of the crude product from the reaction of $[La\{N(SiMe_3)_2\}_3]$ with 3 equivalents of 2-di-tert-butylphosphinophenol (C_6D_6, 298 K, 162 MHz). Inset shows region between 0 and 60 ppm.

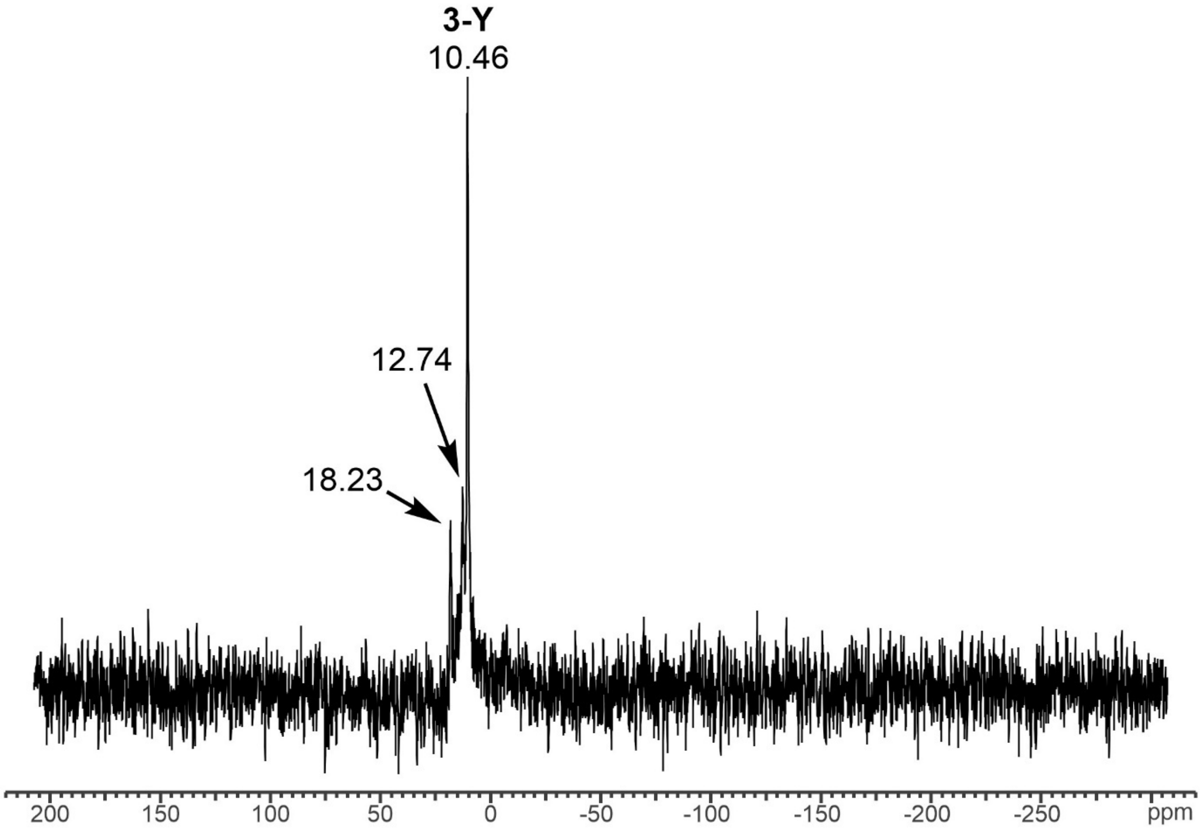

Figure S47: $^{31}P\{H\}$ NMR spectrum of the crude product from the reaction of $[Y\{N(SiMe_3)_2\}_3]$ with 3 equivalents of 2-di-tert-butylphosphinophenol (C_6D_6, 298 K, 162 MHz).

S2. IR data

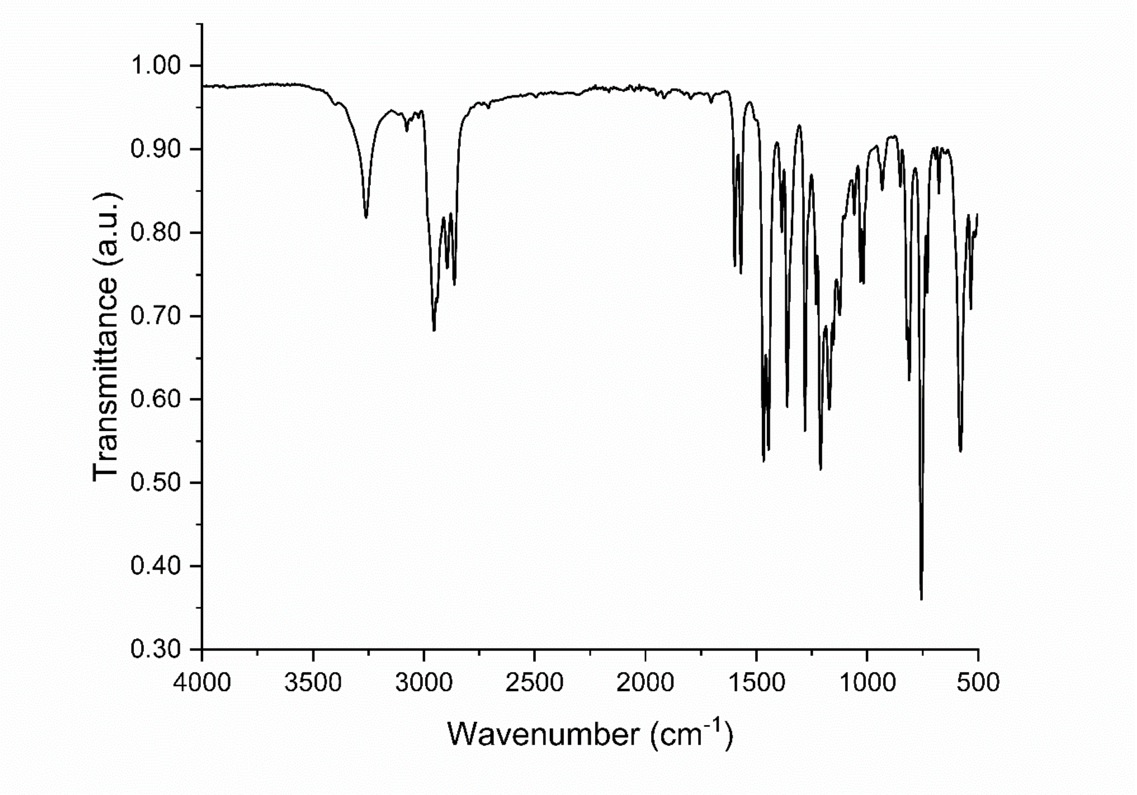

Figure S48: FT-IR Spectrum of 2-di-tert-butylphosphinophenol.

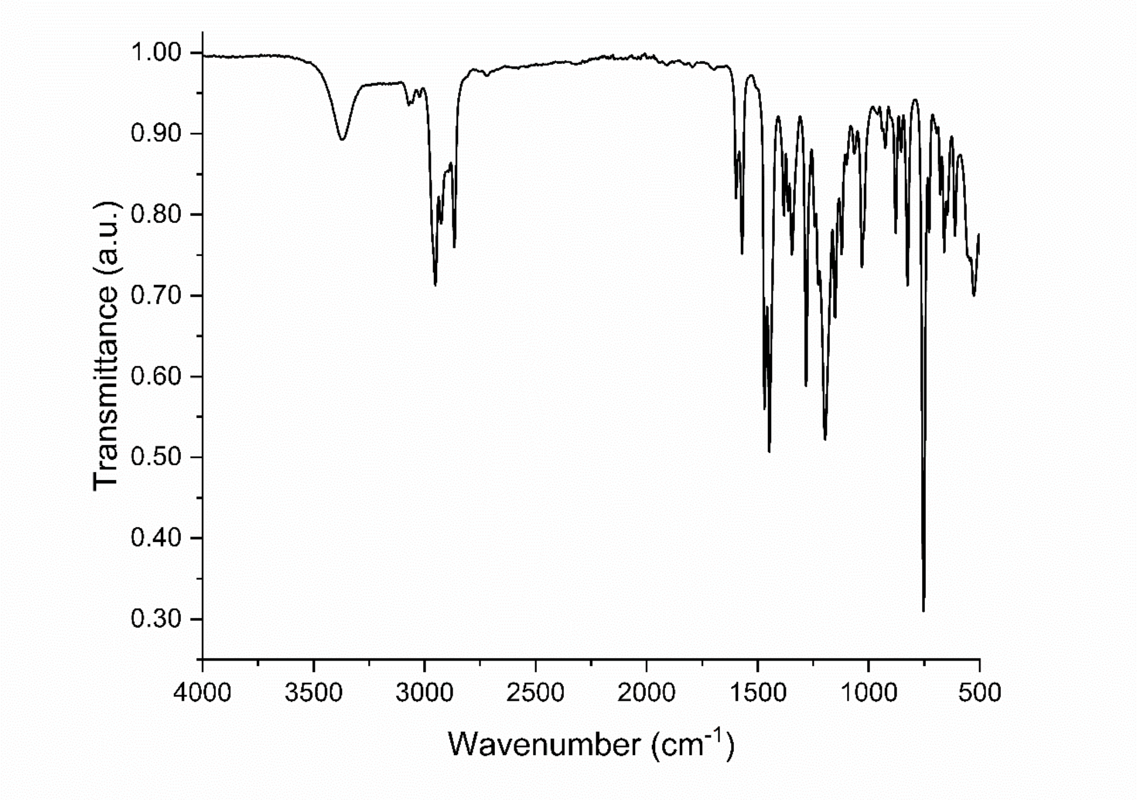

Figure S49: FT-IR Spectrum of 2-di-isopropylphosphinophenol.

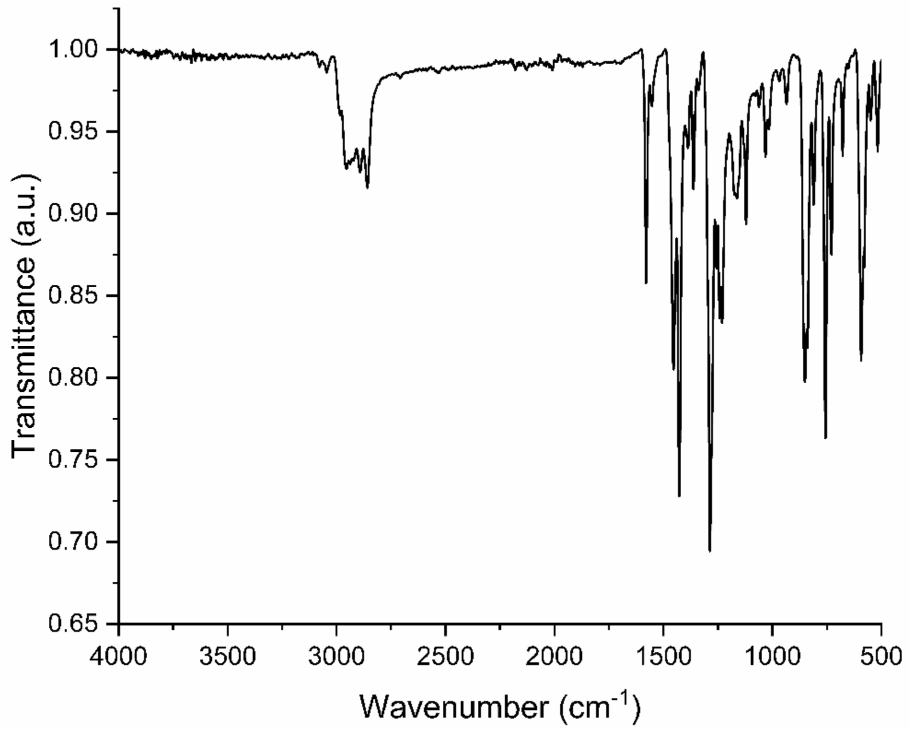

Figure S50: FT-IR Spectrum of **1-La**.

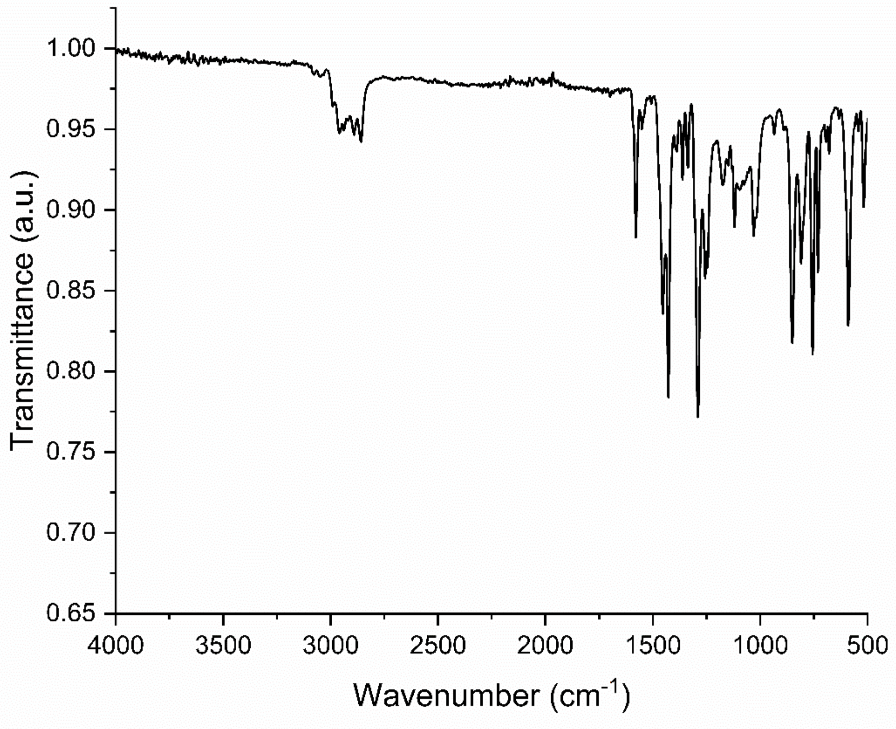

Figure S51: FT-IR Spectrum of **1-Sm**.

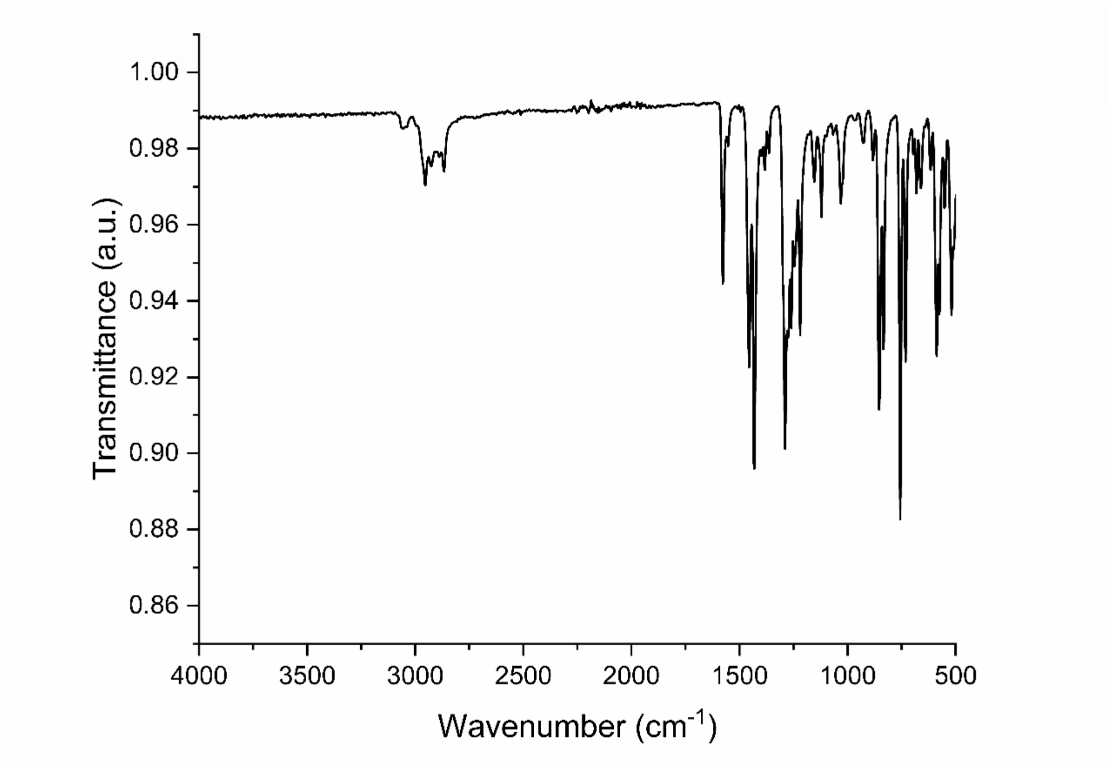

Figure S52: FT-IR Spectrum of **2-La**.

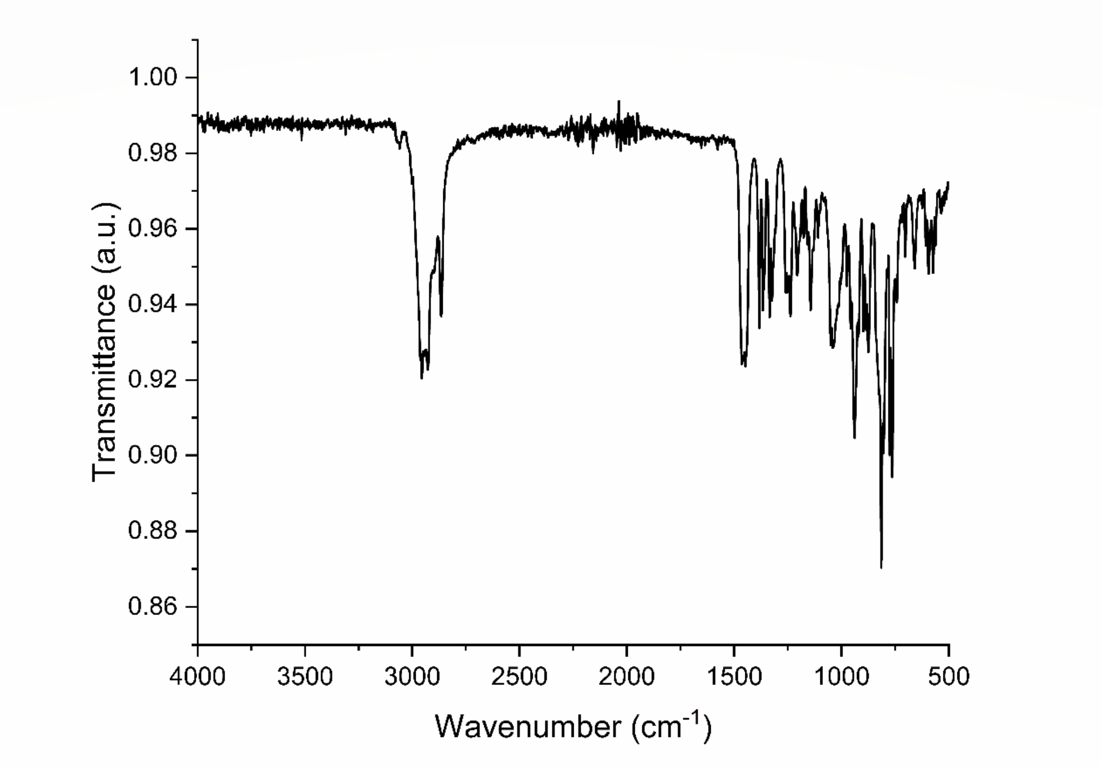

Figure S53: FT-IR Spectrum of **3-Y**.

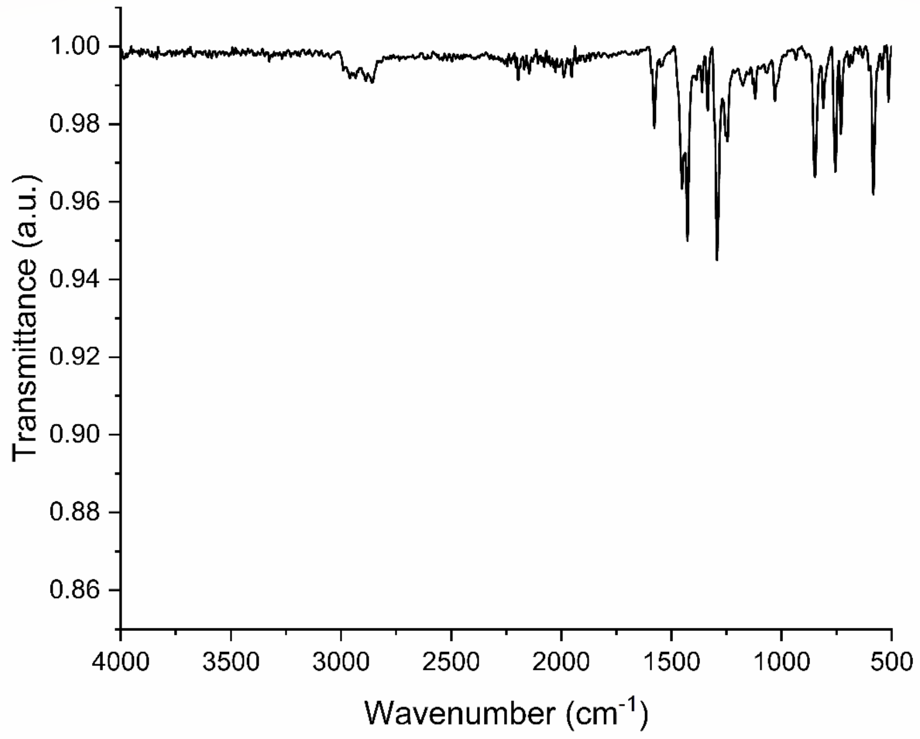

Figure S54: FT-IR Spectrum of **3-La.**

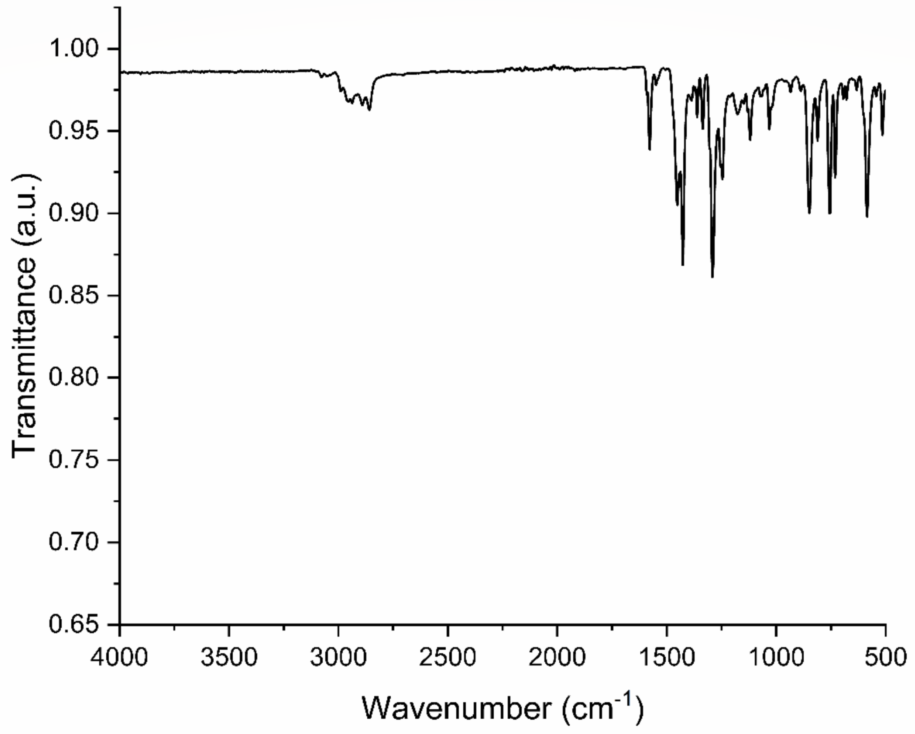

Figure S55: FT-IR Spectrum of **3-Pr**.

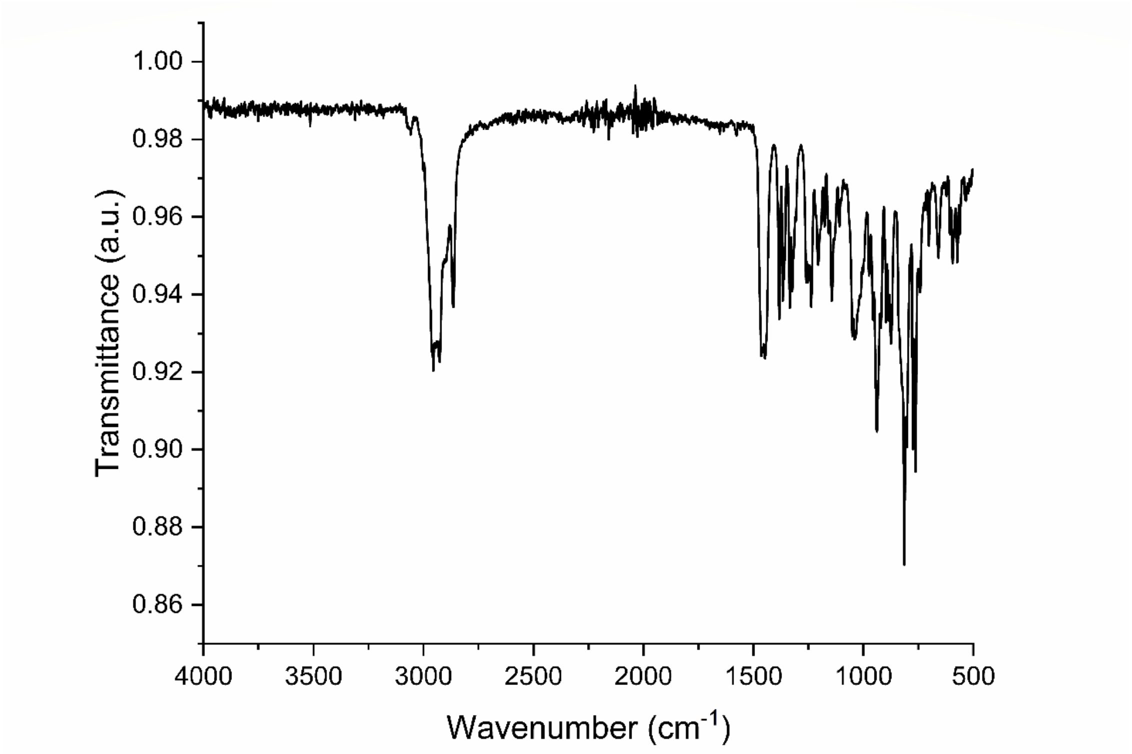

Figure S56: FT-IR Spectrum of **3-Sm**.

S3. Crystallography

Crystallography method

The crystal data for all compounds are compiled in Table S1-3. All crystals were examined using a Bruker D8 Quest diffractometer with a Photon III detector and a microfocus source with Cu-Kα radiation (λ = 1.54178 Å). Intensities were integrated from data recorded on 1° frames by ω or φ rotation. A multi-scan absorption correction method with a beam profile was applied.[1] The structures were solved using SHELXS[2] or SHELXT;[3] the datasets were refined by full-matrix least-squares on reflections with F2≥2σ(F2) values, with anisotropic displacement parameters for all non-hydrogen atoms, and with constrained riding hydrogen geometries;[4] Uiso(H) was set at 1.2 (1.5 for methyl groups) times Ueq of the parent atom. The largest features in final difference syntheses were close to heavy atoms and were of no chemical significance. SHELX[2,3] was employed through OLEX2[4] for structure solution and refinement. The structures have been deposited with the Cambridge Crystallographic Data Centre (CCDC 2394511-2394521 and 2394571). This information can be obtained free of charge from www.ccdc.cam.ac.uk/data_request/cif.

Table S1: Crystallographic data for **$^tBu_2P(C_6H_4)OH$, 1-La, 2-La** and **4**.

	**$^tBu_2P(C_6H_4)OH$**	**1-La**	**2-La**	**4**
Formula	$C_{14}H_{23}OP$	$C_{93}H_{153}La_2O_6P_6$	$C_{86}H_{124}La_2O_6P_6$	$C_{84}H_{131}K_3LaO_6P_6$
Formula Weight	238.29	1830.78	1717.48	1678.91
Crystal Size, mm	0.09×0.13×0.88	0.06×0.08×0.18	0.11×0.14×0.14	0.11×0.14×0.20
Crystal System	monoclinic	triclinic	triclinic	monoclinic
Space group	$P2_1/c$	P-1	P-1	$P2_1/n$
a, Å	12.4765(5)	13.3143(11)	13.0419(6)	17.482(5)
b, Å	10.0111(4)	15.351(2)	13.6428(6)	22.364(6)
c, Å	12.2071(4)	26.489(3)	14.3634(7)	23.567(6)
α, °	90	104.015(9)	64.110(3)	90
β, °	111.272(2)	94.387(6)	79.865(3)	102.640(6)
γ, °	90	110.588(6)	67.832(3)	90
V, Å^3	1420.83(9)	4839.0(9)	2129.0(2)	8991(4)
Z	4	2	1	4
Temperature, K	120(2)	120(2)	120(2)	120(2)
ρ_{calc}, g cm^{-3}	1.114	1.257	1.340	1.240
μ, mm^{-1}	1.536	8.025	9.090	6.284
F(*000*)	520	1926	892	3548
No. of reflections (unique)	17248 (2793)	77549 (19092)	45488 (8335)	57721 (8184)
S[a]	1.04	1.02	1.09	1.13
$R_1(wR_2)$ ($F^2 > 2\sigma(F^2)$)	0.0499 (0.1389)	0.0650 (0.1647)	0.0337 (0.0873)	0.1144 (0.2479)
R_{int}	0.046	0.140	0.053	0.137
Min./max. diff map, Å^{-3}	–0.32, 0.94	–2.07, 1.72	–1.06, 1.48	–0.84, 1.05

[a]Conventional $R = \Sigma||Fo| - |Fc||/\Sigma|Fo|$; $R\text{w} = [\Sigma \text{w}(Fo^2 - \text{Fc}^2)^2/\Sigma w(Fo^2)^2]^{1/2}$; $S = [\Sigma w(Fo^2 - Fc^2)^2/\text{no. data – no. params})]^{1/2}$ for all data.

Table S2: Crystallographic data for **1-Sm**, **3-Y** and **3-La**.

	**1-Sm**	**1-Sm·tol**	**3-Y**	**3-La**
Formula	$C_{42}H_{66}O_3P_3Sm$	$C_{45.5}H_{70}O_3P_3Sm$	$C_{56}H_{89}O_4P_4Y$	$C_{70}H_{105}LaO_4P_4$
Formula Weight	862.20	908.27	1039.06	1273.32
Crystal Size, mm	0.02×0.05×0.05	0.05×0.12×0.20	0.37×0.64×0.66	0.11×0.14×0.20
Crystal System	monoclinic	triclinic	monoclinic	triclinic
Space group	$P2_1/n$	*P*-1	*C*2/*c*	*P*-1
a, Å	10.1136(1)	11.6184(8)	63.711(4)	12.3417(3)
b, Å	18.0304(2)	13.1241(9)	11.8813(7)	15.7328(3)
c, Å	24.2334(2)	17.2669(12)	23.5145(14)	18.9830(4)
α, °	90	83.085(3)	90	86.423(1)
β, °	98.587(1)	73.247(3)	104.577(2)	81.658(1)
γ, °	90	65.722(3)	90	68.732(1)
V, Å^3	1796	2298.2(3)	17227(2)	3398.35(13)
Z	4	2	12	2
Temperature, K	120(2)	120(2)	120(2)	120(2)
ρ_{calc}, g cm^{-3}	1.311	1.313	1.202	1.244
μ, mm^{-1}	11.385	10.857	2.795	6.085
F(*000*)	1796	948	6672	1348
No. of reflections (unique)	98467 (9493)	77887 (9007)	122317 (16874)	64044 (13416)
S[a]	1.07	1.08	1.06	1.03
$R_1(wR_2)$ ($F^2 > 2\sigma(F^2)$)	0.0470 (0.1205)	0.0481 (0.1409)	0.0457 (0.1066)	0.0350 (0.0865)
R_{int}	0.071	0.086	0.041	0.042
Min./max. diff map, Å^{-3}	–0.76, 2.30	–2.30, 2.32	–0.73, 0.97	–1.71, 3.31

[a]Conventional $R = \Sigma||Fo| - |Fc||/\Sigma|Fo|$; Rw = $[\Sigma w(Fo^2 - Fc^2)^2/\Sigma w(Fo^2)^2]^{1/2}$; $S = [\Sigma w(Fo^2 - Fc^2)^2/\text{no. data} - \text{no. params})]^{1/2}$ for all data.

Table S3: Crystallographic data for **3-Ce**, **3-Pr** and **3-Sm**.

	**3-Ce**	**3-Pr**	**3-Sm**	**3-Sm·tol**
Formula	$C_{56}H_{89}CeO_4P_4$	$C_{56}H_{89}O_4P_4Pr$	$C_{56}H_{89}O_4P_4Sm$	$C_{70}H_{105}O_4P_4Sm$
Formula Weight	1090.27	1091.06	1100.50	1284.76
Crystal Size, mm	0.03×0.12×0.17	0.09×0.10×0.19	0.07×0.11×0.20	0.14×0.19×0.20
Crystal System	triclinic	triclinic	triclinic	monoclinic
Space group	*P*-1	*P*-1	*P*-1	$P2_1/n$
a, Å	11.405(5)	11.4811(3)	11.5391(8)	16.7184(6)
b, Å	13.149(9)	13.1643(5)	13.1434(9)	24.4547(9)
c, Å	20.917(15)	20.9850(7)	20.993(2)	16.8001(6)
α, °	85.025(15)	85.159(3)	85.410(4)	90
β, °	75.63(2)	75.596(2)	75.777(4)	90.881(2)
γ, °	68.180(15)	68.108(3)	67.979(3)	90
V, Å^3	2821(3)	2850.3(2)	2860.9(4)	6867.8(4)
Z	2	2	2	4
Temperature, K	120(2)	120(2)	120(2)	120(2)
ρ_{calc}, g cm^{-3}	1.284	1.271	1.278	1.109
μ, mm^{-1}	7.624	7.931	9.076	7.636
F(*000*)	1150	1152	1158	2416
No. of reflections (unique)	71220 (10882)	60556 (12203)	51747 (11259)	81124 (13568)
S[a]	1.04	1.07	1.03	1.02
$R_1(wR_2)$ ($F^2 > 2\sigma(F^2)$)	0.1027 (0.2932)	0.0740 (0.1880)	0.0741 (0.2030)	0.0511 (0.1351)
R_{int}	0.166	0.093	0.097	0.073
Min./max. diff map, Å^{-3}	–2.20, 2.42	–1.78, 3.64	–2.51, 2.72	–0.88, 1.97

[a]Conventional $R = \Sigma||Fo| - |Fc||/\Sigma|Fo|$; Rw = $[\Sigma w(Fo^2 - Fc^2)^2/\Sigma w(Fo^2)^2]^{1/2}$; $S = [\Sigma w(Fo^2 - Fc^2)^2/\text{no. data} - \text{no. params})]^{1/2}$ for all data.

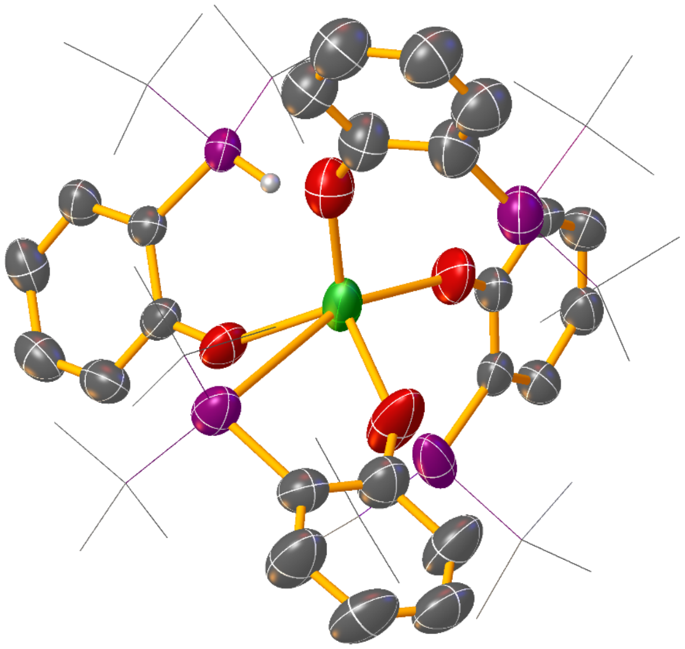

Figure S57: Crystal structure of **3-Ce**, with ellipsoids set at 30% probability level. Tert-butyl groups are displayed as wireframe, and hydrogen atoms have been omitted for clarity, with the exception of the phosphonium group.

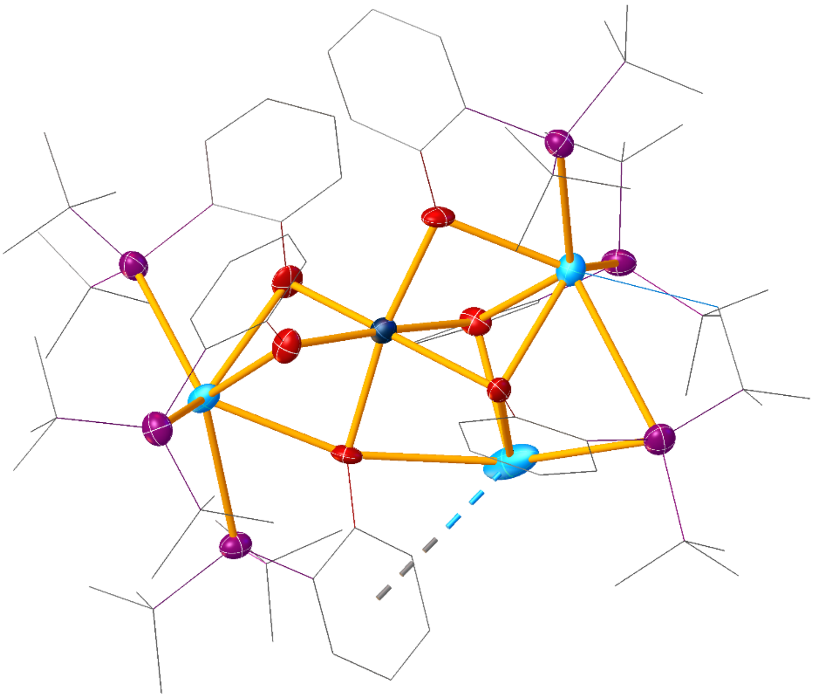

Figure S58: Crystal structure of **4**, with ellipsoids set at 30% probability level. Tert-butyl groups and phenyl rings are displayed as wireframe, and hydrogen atoms have been omitted for clarity.

S4. References

(1) Sheldrick, G. M. *Program for Area Detector Absorption Correction*, Institute for Inorganic Chemistry, University of Göttingen: Göttingen, Germany, 1996.

(2) Sheldrick, G. M. A Short History of SHELX. *Acta Cryst. A* **2008**, *64* (1), 112–122. https://doi.org/10.1107/S0108767307043930.

(3) Sheldrick, G. M. SHELXT – Integrated Space-Group and Crystal-Structure Determination. *Acta Cryst. A* **2015**, *71* (1), 3–8. https://doi.org/10.1107/S2053273314026370.

(4) Dolomanov, O. V.; Bourhis, L. J.; Gildea, R. J.; Howard, J. A. K.; Puschmann, H. OLEX2: A Complete Structure Solution, Refinement and Analysis Program. *J. Appl. Cryst.* **2009**, *42* (2), 339–341. https://doi.org/10.1107/S0021889808042726.
